# Squaramide-Catalyzed Asymmetric Michael Addition/Cyclization Reaction for the Synthesis of Chiral Bisspiro Barbituric Acid–Oxindole Derivatives

**DOI:** 10.3390/molecules30092000

**Published:** 2025-04-30

**Authors:** De-Jun Qiao, Da-Ming Du

**Affiliations:** Key Laboratory of Medicinal Molecule Science and Pharmaceutical Engineering, School of Chemistry and Chemical Engineering, Beijing Institute of Technology, Beijing 100081, China; 3220221413@bit.edu.cn

**Keywords:** organocatalysis, asymmetric catalysis, barbituric acid, oxindole, Michael addition

## Abstract

An efficient stereoselective strategy for the synthesis of chiral bisspiro barbituric acid–oxindole derivatives was developed. The asymmetric Michael addition/cyclization tandem reaction between benzylidene barbituric acids and oxindolylmalonitriles was catalyzed by squaramide catalyst, and the corresponding spirocyclic products were obtained in good-to-high yields (up to 97%) with excellent stereoselectivities (up to >99% ee, >20:1 dr). At the same time, the practicality of the reaction was verified by the gram-scale preparation reaction.

## 1. Introduction

**Figure 1:**
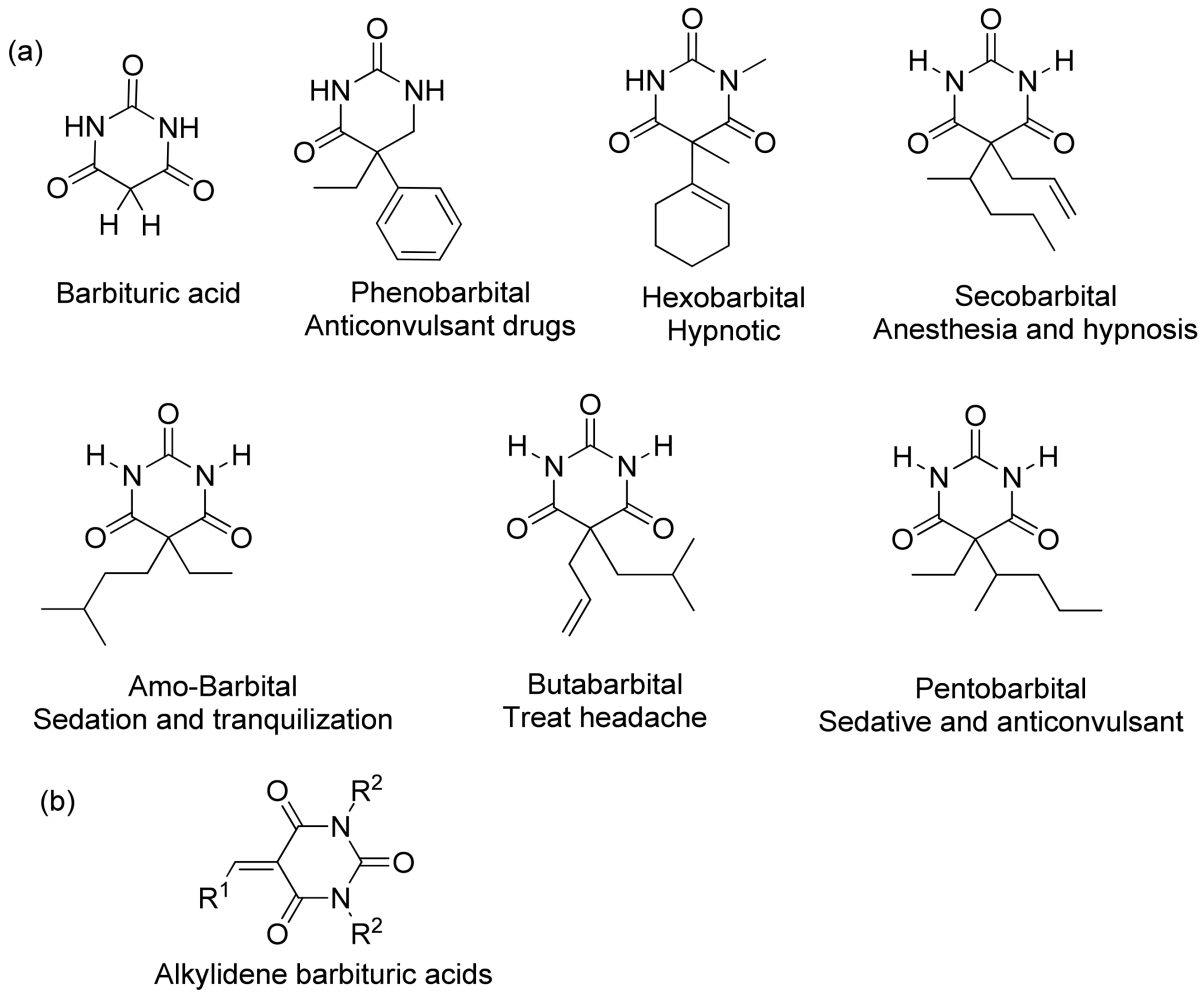
Examples of barbituric acid derivatives. (**a**) barbituric acid derivatives in pharmaceuticals, (**b**) alkylidene barbituric acids.

Barbituric acid was first discovered and named by German chemist Adolf von Baeyer in 1864 [1]. The methylene group at the C5 position of barbituric acid is highly reactive [pka_(DMSO)_ 8.4] due to the influence of two adjacent electron-withdrawing carbonyl groups, so many chemical reactions take place at this position, such as the common Michael addition reaction, substitution reaction, chelation reaction, and Knoevenagel condensation reaction [2]. The biological activity of a series of barbiturates has attracted the attention of many scientists in the field of medicinal chemistry. Barbituric acid derivatives are widely used as anesthetics [3] and sedatives [4] and have anti-convulsant [5], anti-diabetic [6], anti-bacterial [7], anti-cancer [8], and other properties (Figure 1a). When the two H atoms of methylene at 5-position of barbituric acid are replaced by hydrocarbon groups or heterocycles, they can also be used as drug intermediates, such as barbiturates involved in the treatment of certain types of epilepsy [9]. Meanwhile, alkylidene barbituric acids are good Michael acceptors (Figure 1b), which can be applied for constructing many other barbituric acid derivatives [10]. In addition to being biologically active, the photophysical properties of barbiturate derivatives [11] have also been used for colorimetric or thermal detection [12] and have provided some promising dyes or fluorescent probes [13,14]. These applications indicate that barbiturate derivatives have very broad potential value.

**Scheme 1:**
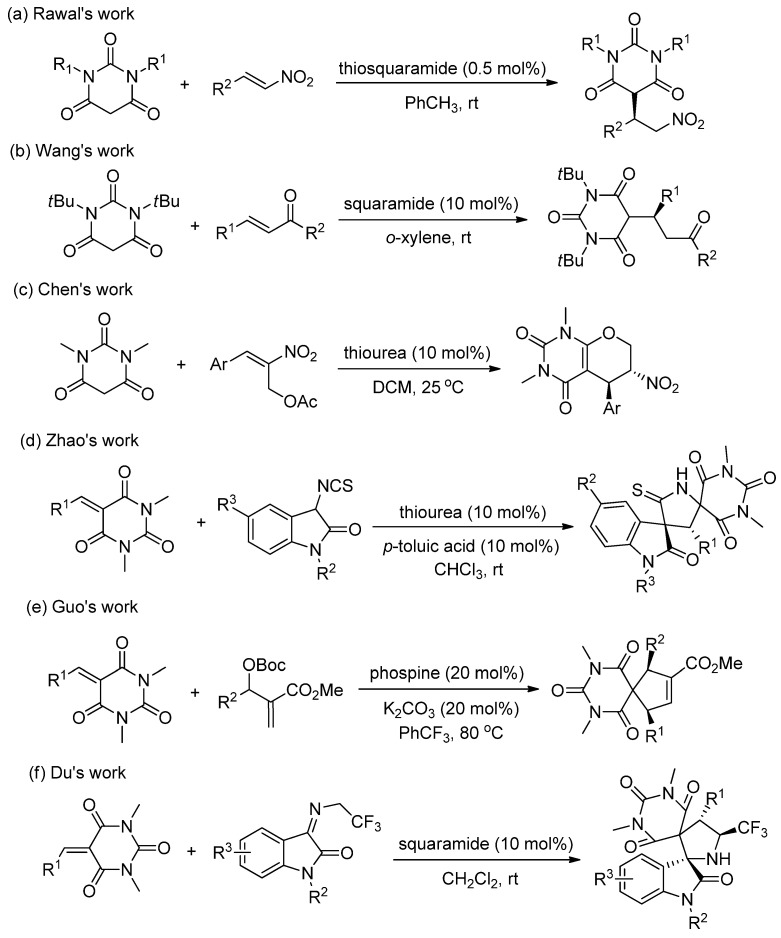
Examples for asymmetric synthesis of barbituric acid derivatives. (**a**) Rawal’s work [15], (**b**) Wang’s work [16], (**c**) Chen’s work [17], (**d**) Zhao’s work [19], (**e**) Guo’s work [20], and (**f**) Du’s work [21].

As mentioned above, barbituric acid derivatives are easily deprotonated owing to their rather low pKa value. Catalytic asymmetric transformations of barbituric acid derivatives have received much attention in recent years for synthesis of chiral barbituric acid derivatives [10]. For example, Rawal et al. reported an enantioselective Michael addition of *N*,*N*′-disubstituted barbituric acid derivatives to β-nitro olefins using chiral thiosquaramide as a bifunctional organocatalyst (Scheme 1a) [15]. The addition products were obtained in high yields with excellent enantioselectivity at catalyst loading as low as 0.5 mol% in toluene at room temperature. Wang and co-workers [16] developed an enantioselective organocatalytic Michael addition of *N*,*N*′-dialkylbarbituric acid derivatives to enones using 10 mol% quinine-derived squaramide catalyst, a series of Michael adducts were obtained in 44–99% yields with 91–99% ee in *o*-xylene at room temperature (Scheme 1b). Chen and co-workers [17] developed a tertiary amine-thiourea-catalyzed domino Michael-oxa-Michael addition reaction of *N*,*N*′-dimethyl barbituric acid and Morita–Baylis-Hillman (MBH) acetate of nitroalkene; the corresponding tetrahydropyrano bicycles were obtained up to 95% yields with dr > 19:1 and up to 95% ee in CH_2_Cl_2_ at 25 °C (Scheme 1c).

Alkylidene barbituric acids as reactive electron-poor alkene derivatives also attracted the attention of researchers in recent years; for example, Zhao’s group reported on a racemic [3 + 2] cycloaddition between alkylidene barbiturates and 3-isothiocyanato oxindoles catalyzed by Et_3_N [18]. In 2016, Zhao et al. developed an *epi*-quinine-thiourea-based thiourea-catalyzed enantioselective version in chloroform, and the corresponding spirobarbiturates were obtained in 80–99% yield with 9:1 to >20:1 dr and 18–99% ee [19]. Guo and co-worker an enantioselective phosphine-catalyzed [3 + 2] annulation of alkylidene barbiturates with MBH adducts, and spirobarbiturates were obtained in excellent diastereoselectivities (4;1–>20:1 dr) and high-to-excellent enantioselectivities (81–99% ee) (Scheme 1e) [20]. Our group developed a squaramide-catalyzed asymmetric Michael/Mannich [3 + 2] cycloaddition reaction of *N*-2,2,2-trifluoroethyl isatin ketimines and barbiturate-based olefins. The corresponding dispirobarbituric acid derivatives were obtained in excellent yields (up to 99% yield) and excellent stereoselectivities (up to 99:1 dr and >99% ee) (Scheme 1f) [21].

In the above-mentioned reports [19,21], the oxindole skeleton also played a crucial role, as these reagents are prone to undergo the tandem reaction with electron-deficient alkenes to construct spirooxindole derivatives. The catalytic asymmetric synthesis of chiral spirooxindoles has also received wide attention in recent years [22]. Continuing on our research project for squaramide-catalytic asymmetric reactions [23], herein, the asymmetric Michael addition/cyclization tandem reaction between barbituric acid derivatives and oxindole derivatives was developed using chiral squaramide as catalyst in order to obtain chiral bisspiro barbituric acid–oxindole derivatives, which may provide potential candidates for future drug design and biological activity research.

## 2. Results and Discussion

### 2.1. Optimization of Reaction Conditions

**Figure 2:**
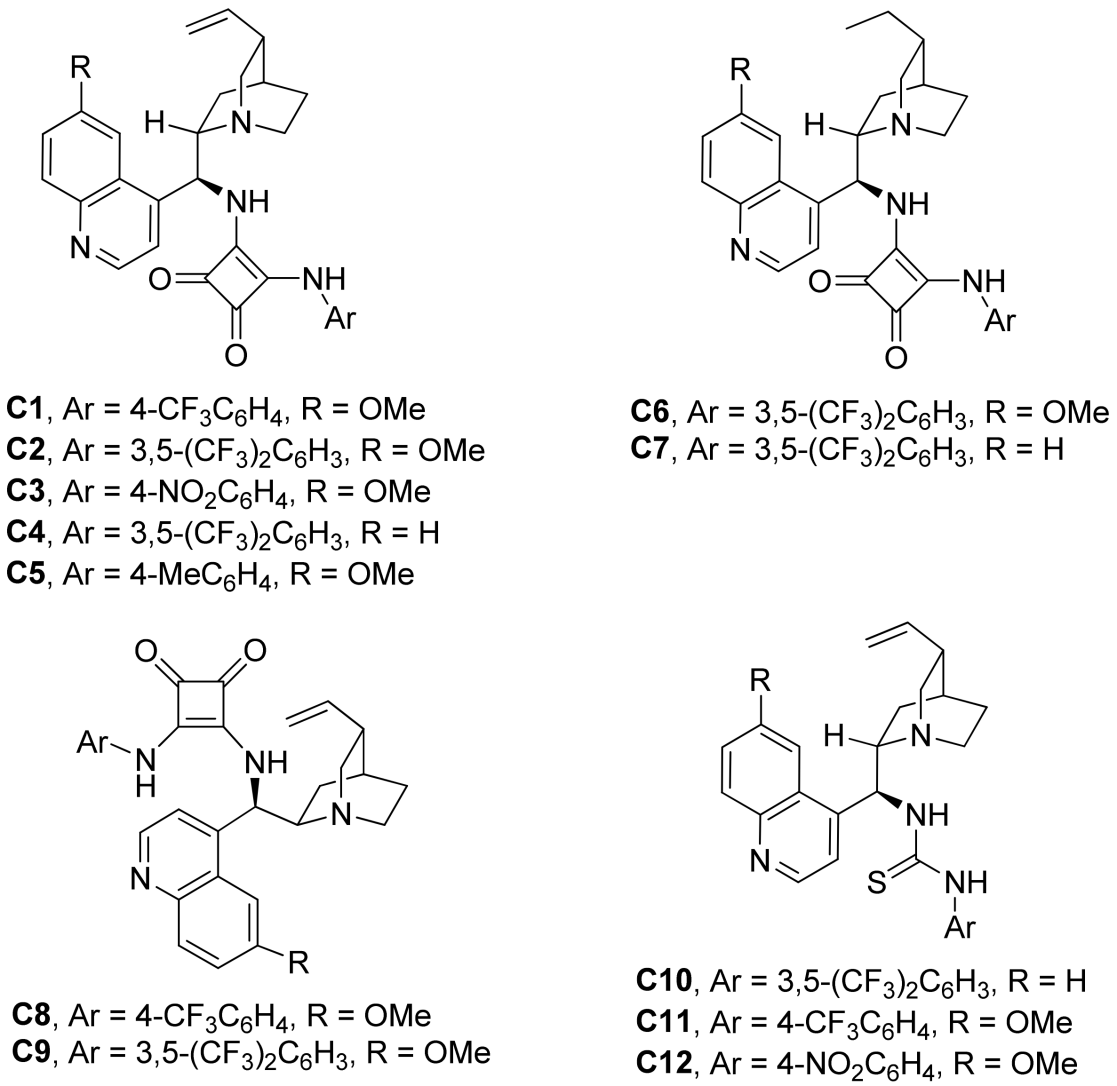
The screened squaramide and thiourea organocatalysts.

To verify our hypothesis, the asymmetric Michael/cyclization reaction of substrates **1a** and **2a** in the presence of quinine-derived squaramide **C1** was selected as the model reaction. We were pleased to find that the asymmetric Michael/cyclization reaction could be completed within 8 h in the presence of 10 mol% **C1** at room temperature and obtained the desired product **3aa** in 88% yield with excellent stereoselectivity (>20:1 dr, 86% ee) (Table 1, entry 1). Encouraged by these excellent results, we screened several organocatalysts with different frameworks for this asymmetric Michael/cyclization reaction (Figure 2) (Table 1, entries 2–12). However, the catalytic yields using catalysts **C2**, **C3**, and **C9** were low (Figure 2) (Table 1, entries 2–12), and the remaining catalysts achieved high yields (>80%) and stereoselectivity (>20:1 dr, >75% ee) (Table 1, entry 2–12). Considering yield and stereoselectivity, **C5** has the best catalytic effect (Table 1, entry 5).

In order to further improve the reaction efficiency, squaramide **C5** was used as a catalyst to optimize other reaction conditions. The effects of solvent, catalyst loading, and reaction temperature on the reaction were evaluated in detail (Table 1, entries 13−19). Solvents play an integral role in the reaction, so we evaluated five other organic solvents, acetonitrile, toluene, THF, chloroform, and methyl tert-butyl ether (MTBE) (Table 1, entries 13−17). However, a series of results show that dichloromethane is still the best solvent. Then, we studied the effect of catalyst loading on the reaction. As the amount of catalyst was halved, the yield and stereoselectivity of the product unfortunately decreased (Table 1, entry 18). As the reaction temperature decreased, the yield of the product did not increase, and its enantioselectivity gradually decreased (Table 1, entry 19). By comparison, the optimal conditions were determined to be benzylidenebarbituric acid and oxindolylmalonitrile as raw materials in the molar ratio of 1.2:1, with 10 mol% **C5** as catalyst, in CH_2_Cl_2_ solvent, at room temperature for 8 h.

### 2.2. Substrate Scope

**Scheme 2:**
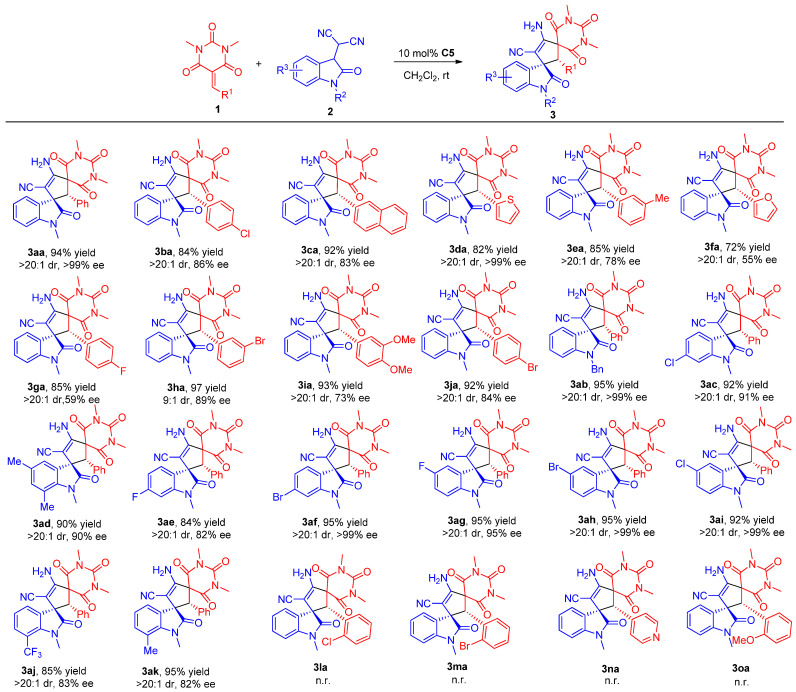
The substrate scope for benzylidene barbituric acids and oxindoles. Unless otherwise noted, the reaction was performed in CH_2_Cl_2_ (1.5 mL) with **1** (0.24 mmol), **2** (0.20 mmol), and catalyst **C5** (0.02 mmol) at room temperature under air for 8 h. The dr values were determined by ^1^H NMR, and the ee values were determined by HPLC.

After the optimum reaction conditions were obtained, the applicability of different substrates to the asymmetric Michael/cyclization reaction was investigated. The result is shown in Scheme 2. Firstly, the effect of benzylidene barbituric acid substrate on the reaction was studied, and the effect of different substitution groups on the reaction was explored. The results show that steric hindrance had a great effect on the reaction. When benzene rings in benzylidenes were meta-substituted or para-substituted, the reaction occurred normally (**3aa**–**ja**), but when benzene rings in benzylidenes were ortho-substituted, the corresponding substrates did not react with **2a** (**3la**–**oa**). The electron effect of substituents also affected the course of the reaction. For electron-withdrawing groups, the substituted substrates bearing bromo- or chloro-substituents showed good yield and stereoselectivity. The substrate with a para-substituted fluoro group was an exception, and the stereoselectivity of the corresponding product was relatively poor, which was considered to be due to the strong electron-withdrawing withdrawing of fluorine. For electron-donating groups, methyl- or methoxy-substituted substrates behaved generally similarly as compared to the bromo-substituted substrate. In addition, the comparison of the yield and enantioselectivity of thienyl-, furanyl-, and pyridinyl-substituted substrates is very interesting. The reaction yield and enantioselectivity of thienyl-substituted substrate were very good (**3da**), the yield and enantioselectivity of furanyl-substituted substrate were moderate (**3fa**), and the reaction of pyridinyl-substituted substrate did not occur (**3na**). This may be ascribed to the electronic effect of these three different heterocycles.

After studying the effect of benzylidene substituents of benzylidene barbituric acids on the reaction, the effect of the *N*-protecting group of oxindolylmalonitriles on the reaction was studied. When R^1^ was a benzyl group or a methyl group, the reaction maintained high selectivity and high efficiency (**3aa** and **3ab**). Subsequently, the different substituents on the phenyl rings of oxindolylmalonitriles were studied. We found that the stereoselectivity of substrates bearing electron-withdrawing substituted indole-phenyl rings was generally better than that of substrates bearing electron-donating substituents. Among the electron-withdrawing groups as substituents (**3ac**, **3ae**–**aj**), all of them maintained excellent yields and stereoselectivities except for the 2-fluoro-substituent. As for the electron-donating groups, the dimethyl-substituted substrate performed better than the monomethyl-substituted substrate (**3ad**, **3ak**).

### 2.3. Scaled-Up Synthesis

**Scheme 3:**
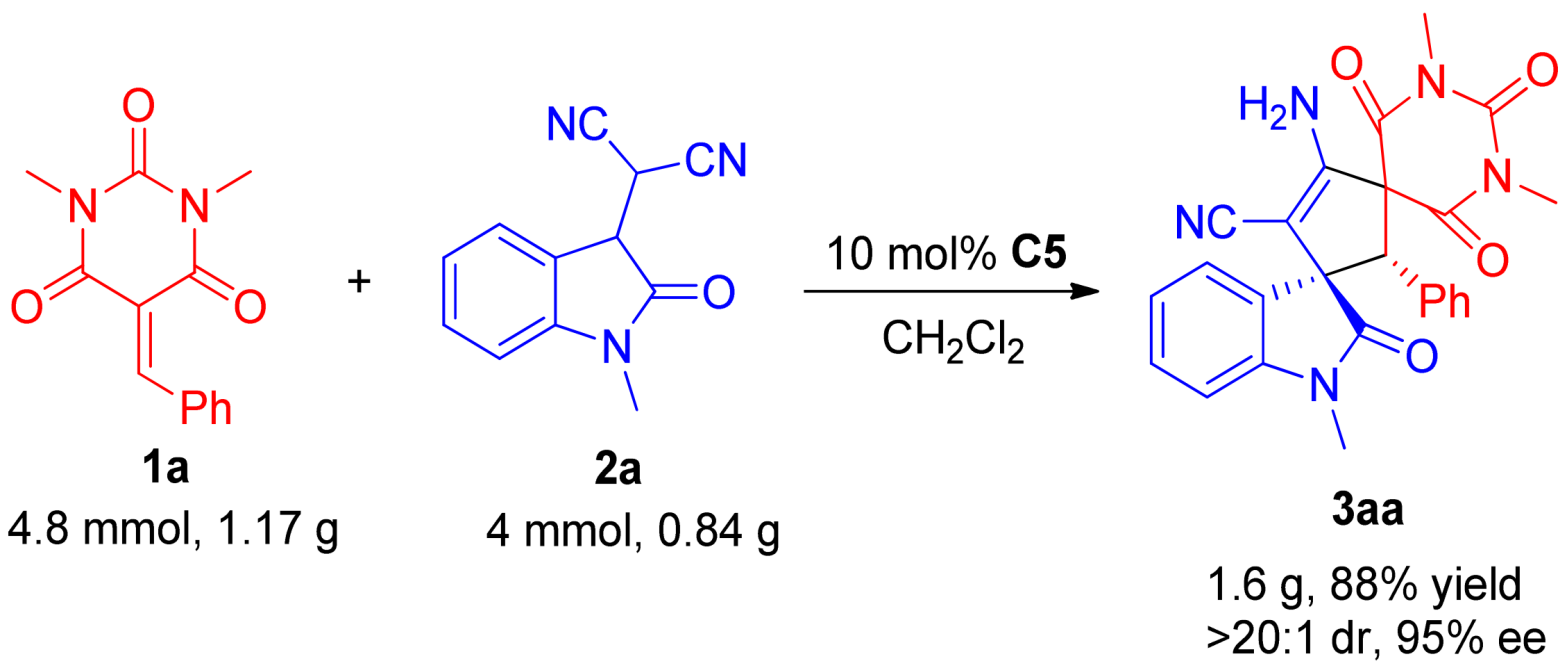
Gram-scale synthesis of 3aa.

In order to further demonstrate the application value of this synthetic method, the gram-scale experiment was conducted under optimized conditions. As shown in Scheme 3, the gram-scale asymmetric Michael/cyclization reaction of **1a** and **2a** proceeded smoothly, and the product **3aa** was obtained in 88% yield with excellent stereoselectivity (>20:1 dr, 95% ee).

### 2.4. Absolute Configuration

**Figure 3:**
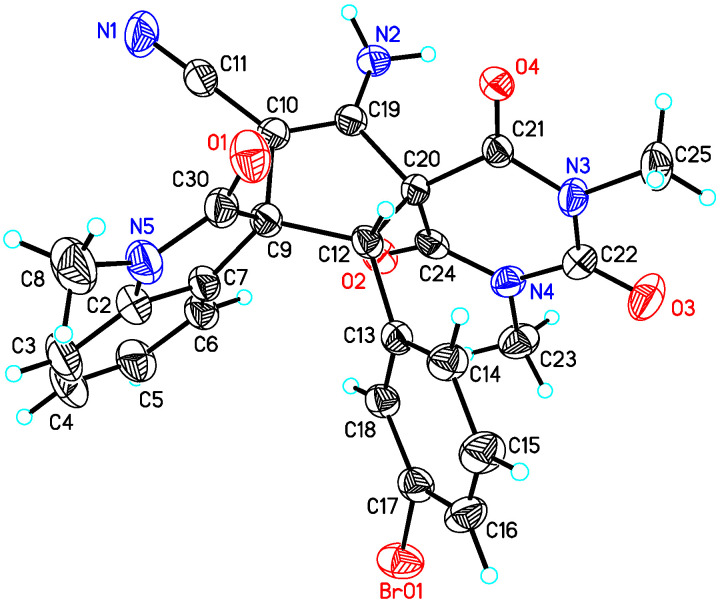
X-ray crystal structure of **3ha** (Displacement ellipsoids are drawn at the 50% probability level; the included solvent molecules were omitted for clarity).

The absolute configuration of the chiral product **3ha** was determined by X-ray diffraction analysis and was found to be (2′*R*,3*S*) (CCDC 2431390) (Figure 3). The absolute configurations of other chiral products were assigned by analogy.

### 2.5. Reaction Mechanism

**Scheme 4:**
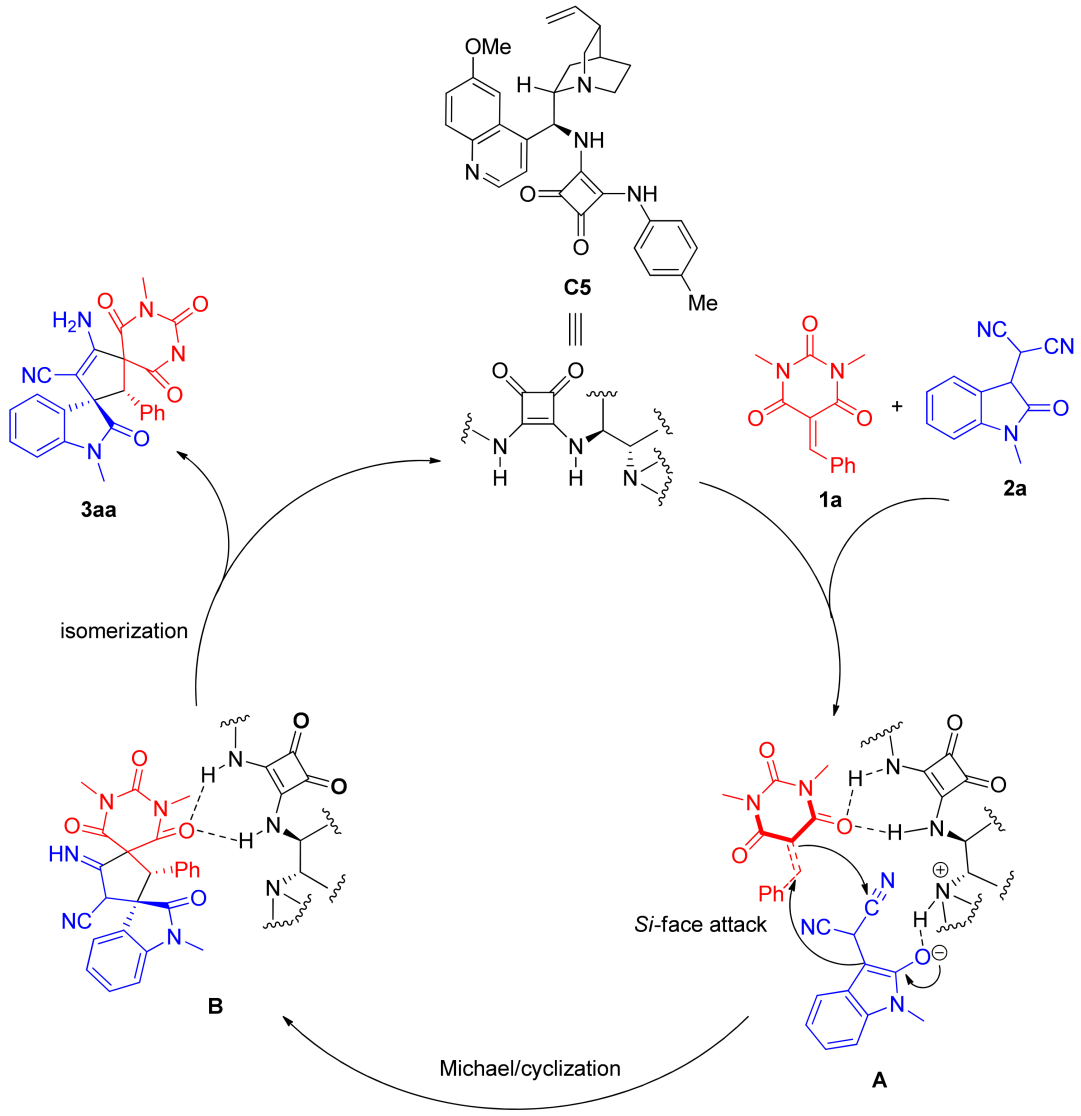
Proposed reaction mechanism.

According to the absolute configuration of the tandem product **3ha** and the catalytic mode of the chiral bifunctional squaramide for a similar reaction [24], a possible transition state model of the catalytic reaction was proposed (Scheme 4). On the one hand, oxindolylmalonitrile **2a** is partially deprotonated by the tertiary amine of catalyst **C5**. On the other hand, benzylidene barbituric acid 1a is activated by forming two hydrogen bonds in the N-H of the squaramide part. Subsequently, the deprotonated-activated oxindolylmalonitrile attacks the *Si*-face of the electron-deficient unsaturated barbituric acid **1a** through the transition state **A**, and the intermolecular Michael addition reaction occurs. At the same time, the resulting Michael addition intermediates undergo further intramolecular cyclization reaction to obtain **B**. Finally, the molecular isomerization reaction occurs to obtain the desired bisspirocyclic product **3aa**, and the bifunctional squaramide catalyst **C5** is regenerated to enter the next catalytic cycle of reaction.

## 3. Materials and Methods

### 3.1. General Information

Commercially available compounds were used without further purification. Solvents were dried according to standard procedures. Column chromatography was performed with silica gel (200−300 mesh). Melting points were determined with a WRX-4 melting-point apparatus and were uncorrected. ^1^H NMR spectra were measured with Bruker Ascend 400 MHz spectrometer and Bruker Ascend 700 MHz spectrometer (Bruker, Karlsurhe, Germany); chemical shifts were reported in δ (ppm) units relative to tetramethylsilane (TMS) as internal standard. ^13^C NMR spectra were measured at 101 MHz with 400 MHz spectrometer and measured at 176 MHz with 700 MHz spectrometer; chemical shifts are reported in ppm relative to tetramethylsilane and referenced to solvent peak (CDCl_3_, δC = 77.00; DMSO, δC = 39.43). High-resolution mass spectra (Electron spray ionization) were measured with an Agilent 6520 Accurate-Mass Q-TOF MS system (Agilent, Santa Clara, CA, USA) equipped with an electrospray ionization (ESI) source. Optical rotations were measured with a Krüss P8000 polarimeter (Krüss, Hamburg, Germany). Optical rotations at the indicated concentration with the units of g/100 mL. Enantiomeric excesses were determined by chiral HPLC analysis using an Agilent 1200 LC instrument (Agilent, Santa Clara, CA, USA) with a Daicel Chiralpak ADH, IA, or IC column (Daicel, Beijing, China).

### 3.2. Experimental Materials for Tandem Reactions

First, **1a**–**1j** were prepared according to literature reported by Neumann and co-workers [25]. Then, **2a**–**2k** were prepared according to literature reported by Lin and co-workers [24]. The chiral organocatalysts were prepared by following the reported procedures [26,27,28,29].

### 3.3. Procedure for the Synthesis of Racemates of ***3***

To a dried small vial, benzylidene barbituric acid **1** (0.24 mmol), oxindolylmalonitrile **2** (0.2 mmol), Et_3_N (1.0 mg, 0.01 mmol, 0.05 equiv.), and CH_2_Cl_2_ (1 mL) were added. After stirring at room temperature under air without gas protection for 8 h, the reaction mixture was concentrated and directly purified by silica gel column chromatography to afford the racemates of **3**.

### 3.4. Procedure for the Asymmetric Michael/Cyclization Reaction

To a dried small vial, barbituric acid **1** (0.24 mmol), oxindolylmalonitrile **2** (0.2 mmol), chiral organocatalyst **C5** (5.08 mg, 0.01 mmol, 0.05 equiv), and CH_2_Cl_2_ (1.0 mL) were added. After stirring at room temperature under air without gas protection for 8 h, the reaction mixture was concentrated and directly purified by silica gel column chromatography (200–300 mesh) using ethyl acetate/petroleum ether (1:2) as eluent to afford the desired products **3**. 

(2′*R*,3*S*)-4′-Amino-1,1″,3″-trimethyl-2,2″,4″,6″-tetraoxo-2′-phenyl-1″,3″,4″,6″-tetrahydro-2″*H*-dispiro[indoline-3,1′-cyclopentane-3′,5″-pyrimidin]-4′-ene-5′-carbonitrile (**3aa**). According to the general procedure from **1a** (58.6 mg, 0.24 mmol) and **2a** (42.2 mg, 0.2 mmol) to obtain 85.6 mg (94% yield) compound **3aa** as a yellow solid, m.p. 192−195 °C. HPLC (Daicel Chiralpak ADH, *n*-hexane/2-propanol = 70:30, flow rate 1.0 mL/min, detection at 254 nm): *t*_R_ = 9.2 min (major), >99% ee. [α]_D_^25^ = +9.9 (*c* = 0.5, CH_2_Cl_2_). ^1^H NMR (700 MHz, DMSO-d_6_): *δ* 8.05 (dd, *J*_1_ = 7.7 Hz, *J*_2_ = 0.7 Hz, 1H), 7.28 (td, *J*_1_ = 7.7 Hz, *J*_2_ = 1.4 Hz 1H), 7.15–7.18 (m, 4H), 7.06 (t, *J* = 8.0 Hz, 2H), 6.86 (d, *J* = 7.7 Hz, 1H), 6.76 (d, *J* = 7.0 Hz, 2H), 4.24 (s, 1H), 3.07 (s, 3H), 2.99 (s, 3H), 2.87 (s, 3H) ppm. ^13^C NMR (176 MHz, DMSO-d_6_): *δ* 176.5, 168.6, 167.8, 158.4, 149.8, 143.0, 131.1, 129.4, 129.3, 129.0, 128.3, 128.0, 127.3, 122.3, 116.3, 108.7, 78.5, 68.1, 64.8, 63.4, 28.5, 28.3, 26.4 ppm. (see Supplementary Materials) HRMS (ESI): *m*/*z* calcd. for C_25_H_22_N_5_O_4_ [M + H]^+^ 456.1666, found 456.1686.

(2′*R*,3*S*)-4′-Amino-2′-(4-chlorophenyl)-1,1″,3″-trimethyl-2,2″,4″,6″-tetraoxo-1″,3″,4″,6″-tetrahydro-2″*H*-dispiro[indoline-3,1′-cyclopentane-3′,5″-pyrimidin]-4′-ene-5′-carbonitrile (**3ba**). According to the general procedure from **1b** (66.7 mg, 0.24 mmol) and **2a** (42.2 mg, 0.2 mmol) to obtain 82.2 mg (84% yield) compound **3ba** as a yellow solid, m.p. 183–185 °C. HPLC (Daicel Chiralpak IC, *n*-hexane/2-propanol = 70:30, flow rate 1.0 mL/min, detection at 254 nm): *t*_R_ = 6.8 min (minor), *t*_R_ = 9.4 min (major), 86% ee. [α]_D_^25^ = +12.2 (*c* = 0.34, CH_2_Cl_2_). ^1^H NMR (400 MHz, CDCl_3_): *δ* 8.12 (d, *J* = 7.2 Hz, 1H), 7.29–7.25 (m, 1H), 7.17 (td, *J*_1_ = 7.6 Hz, *J*_2_ = 0.8 Hz, 1H), 7.01 (d, *J* = 8.4 Hz, 2H), 6.78 (d, *J* = 8.8 Hz, 2H), 6.67 (d, *J* = 7.6 Hz, 1H), 5.65 (s, 2H), 4.37 (s, 1H), 3.20 (s, 3H), 3.03 (s, 3H), 2.98 (s, 3H) ppm. ^13^C NMR (101 MHz, CDCl_3_): *δ* 176.8, 168.6, 167.1, 158.0, 149.8, 143.9, 135.6, 131.1, 129.8, 129.3, 128.5, 128.2, 127.3, 123.2, 115.3, 108.7, 83.2, 68.6, 64.6, 63.9, 29.3, 28.9, 26.8 ppm. HRMS (ESI): *m*/*z* calcd. for C_25_H_21_ClN_5_O_4_ [M + H]^+^ 490.1277, found 490.1301.

(2′*R*,3*S*)-4′-Amino-1,1″,3″-trimethyl-2′-(naphthalen-2-yl)-2,2″,4″,6″-tetraoxo-1″,3″,4″,6″-tetrahydro-2″*H*-dispiro[indoline-3,1′-cyclopentane-3′,5″-pyrimidin]-4′-ene-5′-carbonitrile (**3ca**). According to the general procedure from **1c** (70.6 mg, 0.24 mmol) and **2a** (42.2 mg, 0.2 mmol) to obtain 93.0 mg (92% yield) compound **3ca** as a yellow solid, m.p. 201–203 °C. HPLC (Daicel Chiralpak IC, *n*-hexane/2-propanol = 70:30, flow rate 1.0 mL/min, detection at 254 nm): *t*_R_ = 13.0 min (minor), *t*_R_ = 16.7 min (major), 83% ee. [α]_D_^25^ = +16.5 (*c* = 0.5, CH_2_Cl_2_). ^1^H NMR (400 MHz, DMSO-d_6_): *δ* 8.18 (d, *J* = 7.6 Hz, 1H), 7.85 (d, *J* = 8.0 Hz, 1H), 7.76 (t, *J* = 9.4 Hz, 2H), 7.61 (td, *J*_1_ = 7.6 Hz, *J*_2_ = 1.0 Hz, 1H), 7.50 (t, *J* = 7.4 Hz, 1H), 7.32–7.22 (m, 4H), 7.12 (t, *J* = 7.8 Hz, 1H), 7.01 (d, *J* = 7.2 Hz, 1H), 6.83 (d, *J* = 7.6 Hz, 1H), 5.49 (s, 1H), 2.88 (s, 3H), 2.81 (s, 3H), 2.60 (s, 3H) ppm. ^13^C NMR (101 MHz, DMSO-d_6_): *δ* 176.5, 168.7, 168.0, 158.5, 149.4, 143.1, 133.0, 131.9, 129.4, 129.3, 129.1, 128.5, 127.5, 127.2, 126.7, 125.9, 123.8, 122.5, 120.7, 116.3, 108.9, 79.1, 68.3, 63.6, 55.9, 28.3, 26.4 ppm. HRMS (ESI): *m*/*z* calcd. for C_29_H_24_N_5_O_4_ [M + H]^+^ 506.1823, found 506.1818.

(2′*R*,3*S*)-4′-Amino-1,1″,3″-trimethyl-2,2″,4″,6″-tetraoxo-2′-(thiophen-2-yl)-1″,3″,4″,6″-tetrahydro-2″*H*-dispiro[indoline-3,1′-cyclopentane-3′,5″-pyrimidin]-4′-ene-5′-carbonitrile (**3da**). According to the general procedure from **1d** (60.0 mg, 0.24 mmol) and **2a** (42.2 mg, 0.2 mmol) to obtain 75.6 mg (82% yield) compound **3da** as a brown solid, m.p. 214–215 °C. HPLC (Daicel Chiralpak IA, *n*-hexane/2-propanol/ethyl acetate = 80:10:10, flow rate 1.0 mL/min, detection at 254 nm): *t*_R_ = 19.3 min (minor), *t*_R_ = 26.4 min (major); >99% ee. [α]_D_^25^ = +18.5 (*c* = 0.4, CH_2_Cl_2_). ^1^H NMR (700 MHz, DMSO-d_6_): *δ* 8.06 (d, *J* = 7.7 Hz, 1H), 7.36–7.32 (m, 2H), 7.20–7.16 (m, 3H), 6.94 (d, *J* = 7.7 Hz, 1H), 6.78 (dd, *J*_1_ = 4.9 Hz, *J*_2_ = 4.2 Hz, 1H), 6.68 (d, *J* = 3.5 Hz, 1H), 4.62 (s, 1H), 3.18 (s, 3H), 3.05 (s, 3H), 2.85 (s, 3H) ppm. ^13^C NMR (176 MHz, DMSO-d_6_): *δ* 176.2, 168.3, 167.3, 158.0, 150.0, 143.4, 132.5, 129.8, 129.4, 128.2, 128.0, 127.8, 126.3, 122.4, 116.2, 108.7, 78.5, 68.4, 63.0, 59.7, 28.7, 28.6, 26.5 ppm. HRMS (ESI): *m*/*z* calcd. for C_23_H_20_N_5_O_4_S [M + H]^+^ 462.1231, found 462.1253.

(2′*R*,3*S*)-4′-Amino-1,1″,3″-trimethyl-2,2″,4″,6″-tetraoxo-2′-(m-tolyl)-1″,3″,4″,6″-tetrahydro-2″*H*-dispiro[indoline-3,1′-cyclopentane-3′,5″-pyrimidin]-4′-ene-5′-carbonitrile (**3ea**). According to the general procedure from **1e** (61.92 mg, 0.24 mmol) and **2a** (42.2 mg, 0.2 mmol) to obtain 79.7 mg (85% yield) compound **3ea** as a white solid, m.p. 194–196 °C. HPLC (Daicel Chiralpak ADH, *n*-hexane/2-propanol = 70:30, flow rate 1.0 mL/min, detection at 254 nm): *t*_R_ = 6.5 min (minor), *t*_R_ = 11.5 min (major), 78% ee. [α]_D_^25^ = +14.8 (*c* = 0.5, CH_2_Cl_2_). ^1^H NMR (400 MHz, DMSO-d_6_): *δ* 8.06 (dd, *J*_1_ = 7.6 Hz, *J*_2_ = 0.8 Hz, 1H), 7.28 (td, *J* = 7.6 Hz, *J*_2_ = 1.2 Hz, 1H), 7.17 (t, *J* = 7.2 Hz, 3H), 6.98–6.91 (m, 2H), 6.85 (d, *J* = 7.6 Hz, 1H), 6.58 (s, 1H), 6.54 (d, *J* = 7.2 Hz, 1H), 4.20 (s, 1H), 3.08 (s, 3H), 2.98 (s, 3H), 2.86 (s, 3H), 2.03 (s, 3H) ppm. ^13^C NMR (101 MHz, DMSO-d_6_): *δ* 176.5, 168.6, 167.8, 158.4, 149.8, 143.0, 137.2, 131.1, 130.1, 129.5, 129.2, 128.4, 127.8, 127.3, 126.4, 122.1, 116.3, 108.7, 78.5, 68.1, 64.7, 63.4, 28.5, 28.3, 26.4, 20.5. ppm. HRMS (ESI): *m*/*z* calcd. for C_26_H_24_N_5_O_4_ [M + H]^+^ 470.1823, found 470.1812.

(2′*R*,3*S*)-4′-Amino-2′-(furan-2-yl)-1,1″,3″-trimethyl-2,2″,4″,6″-tetraoxo-1″,3″,4″,6″-tetrahydro-2″*H*-dispiro[indoline-3,1′-cyclopentane-3′,5″-pyrimidin]-4′-ene-5′-carbonitrile (**3fa**). According to the general procedure from **1f** (56.2 mg, 0.24 mmol) and **2a** (42.2 mg, 0.2 mmol) to obtain 64.1 mg (72% yield) compound **3fa** as a brown solid, m.p. 174–176 °C. HPLC (Daicel Chiralpak IA, *n*-hexane/2-propanol = 70:30, flow rate 1.0 mL/min, detection at 254 nm): *t*_R_ = 6.6 min (minor), *t*_R_ = 8.2 min (major), 55% ee. [α]_D_^25^ = −2.0 (*c* = 0.33, CH_2_Cl_2_). ^1^H NMR (700 MHz, DMSO-d_6_): *δ* 7.93 (dd, *J* = 7.7 Hz, 1H), 7.38 (d, *J* = 1.4 Hz, 1H), 7.32 (td, *J*_1_ = 7.7 Hz, *J*_2_ = 0.7 Hz, 1H), 7.17–7.12 (m, 3H), 6.97 (d, *J* = 7.7 Hz, 1H), 6.15 (dd, *J*_1_ = 3.2 Hz, *J*_2_ = 1.8 Hz, 1H), 5.74 (d, *J* = 2.8 Hz, 1H), 4.41 (s, 1H), 3.19 (s, 3H), 3.10 (s, 3H), 2.94 (s, 3H) ppm. ^13^C NMR (176 MHz, DMSO-d_6_): *δ* 176.0, 168.2, 167.2, 157.7, 150.2, 145.9, 144.0, 142.9, 129.3, 128.2, 127.4, 122.3, 116.0, 110.7, 110.4, 108.6, 78.8, 66.9, 61.7, 56.5, 28.7, 28.6, 26.5 ppm. HRMS (ESI): *m*/*z* calcd. for C_23_H_20_N_5_O_5_ [M + H]^+^ 446.1459, found 446.1472.

(2′*R*,3*S*)-4′-Amino-2′-(4-fluorophenyl)-1,1″,3″-trimethyl-2,2″,4″,6″-tetraoxo-1″,3″,4″,6″-tetrahydro-2″*H*-dispiro[indoline-3,1′-cyclopentane-3′,5″-pyrimidin]-4′-ene-5′-carbonitrile (**3ga**). According to the general procedure from **1g** (62.9 mg, 0.24 mmol) and **2a** (42.2 mg, 0.2 mmol) to obtain 80.4 mg (85% yield) compound **3ga** as a pink solid, m.p. 178–179 °C. HPLC (Daicel Chiralpak IA, *n*-hexane/2-propanol/ethyl acetate = 80:15:5, flow rate 1.0 mL/min, detection at 254 nm): *t*_R_ = 13.4 min (minor), *t*_R_ = 21.9 min (major); 59% ee. [α]_D_^25^ = +9.0 (*c* = 0.8, CH_2_Cl_2_). ^1^H NMR (700 MHz, DMSO-d_6_): *δ* 8.03 (d, *J* = 7.0 Hz, 1H), 7.30 (t, *J* = 7.7 Hz, 1H), 7.20–7.16 (m, 3H), 6.94–6.88 (m, 3H), 6.83–6.80 (m, 2H), 4.25 (s, 1H), 3.09 (s, 3H), 3.01 (s, 3H), 2.91 (s, 3H) ppm. ^13^C NMR (176 MHz, DMSO-d_6_): *δ* 176.4, 168.5, 167.7, 162.1 (^1^*J*_C–F_ = 246.9 Hz), 158.3, 149.9, 143.0, 131.5 (^3^*J*_C–F_ = 8.3 Hz), 129.4, 128.1, 127.3 (^4^*J*_C–F_ = 2.6 Hz), 127.2, 122.4, 116.2, 115.0 (^2^*J*_C–F_ = 21.5 Hz), 108.8, 78.3, 67.9, 63.7, 63.4, 28.6, 28.4, 26.4 ppm. ^19^F NMR (659 MHz, DMSO-d_6_): δ −112.1. HRMS (ESI): *m*/*z* calcd. for C_25_H_21_FN_5_O_4_ [M + H]^+^ 474.1572, found 474.1587.

(2′*R*,3*S*)-4′-Amino-2′-(3-bromophenyl)-1,1″,3″-trimethyl-2,2″,4″,6″-tetraoxo-1″,3″,4″,6″-tetrahydro-2″*H*-dispiro[indoline-3,1′-cyclopentane-3′,5″-pyrimidin]-4′-ene-5′-carbonitrile (**3ha**). According to the general procedure from **1h** (77.3 mg, 0.24 mmol) and **2a** (42.2 mg, 0.2 mmol) to obtain 103.4 mg (97% yield) compound **3ha** as a pink solid, m.p. 194–195 °C. HPLC (Daicel Chiralpak ADH, *n*-hexane/2-propanol = 75:25, flow rate 1.0 mL/min, detection at 254 nm): *t*_R_ = 11.8 min (minor), *t*_R_ = 14.5 min (major); 89% ee. [α]_D_^25^ = +14.9 (*c* = 0.5, CH_2_Cl_2_). ^1^H NMR (700 MHz, DMSO-d_6_): *δ* 8.03 (d, *J* = 7.7 Hz, 1H), 7.39 (d, *J* = 8.4 Hz, 1H), 7.32 (t, *J* = 7.7 Hz, 1H), 7.23–7.18 (m, 3H), 7.07 (t, *J* = 8.0 Hz, 1H), 6.93 (s, 1H), 6.90 (d, *J* = 7.7 Hz, 1H), 6.85 (d, *J* = 7.7 Hz, 1H), 4.20 (s, 1H), 3.10 (s, 3H), 3.01 (s, 3H), 2.92 (s, 3H) ppm. ^13^C NMR (176 MHz, DMSO-d_6_): *δ* 176.2, 168.2, 167.7, 158.2, 149.8, 143.0, 133.5, 131.9, 131.6, 130.3, 129.5, 129.1, 127.9, 127.1, 122.3, 121.0, 116.2, 109.0, 78.2, 67.9, 63.6, 63.3, 28.6, 28.3, 26.4 ppm. HRMS (ESI): *m*/*z* calcd. for C_25_H_21_^79^BrN_5_O_4_ [M + H]^+^ 534.0771, found 534.0786; calcd. for C_25_H_21_^81^BrN_5_O_4_ [M + H]^+^ 536.0751, found 536.0770.

(2′*R*,3*S*)-4′-Amino-2′-(3,4-dimethoxyphenyl)-1,1″,3″-trimethyl-2,2″,4″,6″-tetraoxo-1″,3″,4″,6″-tetrahydro-2″*H*-dispiro[indoline-3,1′-cyclopentane-3′,5″-pyrimidin]-4′-ene-5′-carbonitrile (**3ia**). According to the general procedure from **1i** (73.0 mg, 0.24 mmol) and **2a** (42.2 mg, 0.2 mmol) to obtain 90.2 mg (93% yield) compound **3ia** as a yellow solid, m.p. 203–205 °C. HPLC (Daicel Chiralpak ADH, *n*-hexane/2-propanol = 80:20, flow rate 1.0 mL/min, detection at 254 nm): *t*_R_ = 19.0 min (minor), *t*_R_ = 21.3 min (major), 73% ee. [α]_D_^25^ = +49.1 (*c* = 0.7, CH_2_Cl_2_). ^1^H NMR (700 MHz, DMSO-d_6_): *δ* 8.10 (d, *J* = 7.7 Hz, 1H), 7.31 (t, *J* = 7.7 Hz, 1H), 7.18 (t, *J* = 7.7 Hz, 3H), 6.90 (d, *J* = 7.7 Hz, 1H), 6.65 (d, *J* = 8.4 Hz, 1H), 6.37 (dd, *J*_1_ = 8.0 Hz, *J*_2_ = 1.4 Hz, 1H), 6.21 (s, 1H), 4.17 (s, 1H), 3.61 (s, 3H), 3.33 (s, 3H), 3.11 (s, 3H), 3.00 (s, 3H), 2.87 (s, 3H) ppm. ^13^C NMR (176 MHz, DMSO-d_6_): *δ* 176.6, 168.7, 167.9, 158.5, 150.0, 148.9, 147.5, 143.2, 129.2, 128.8, 127.2, 123.0, 122.8, 122.1, 116.4, 111.5, 110.7, 108.9, 78.3, 68.2, 64.5, 63.5, 55.1, 55.0, 28.6, 28.4, 26.4 ppm. HRMS (ESI): *m*/*z* calcd. for C_27_H_26_N_5_O_6_ [M + H]^+^ 516.1878, found 516.1895.

(2′*R*,3*S*)-4′-Amino-2′-(4-bromophenyl)-1,1″,3″-trimethyl-2,2″,4″,6″-tetraoxo-1″,3″,4″,6″-tetrahydro-2″*H*-dispiro[indoline-3,1′-cyclopentane-3′,5″-pyrimidin]-4′-ene-5′-carbonitrile (**3ja**). According to the general procedure from **1j** (77.3 mg, 0.24 mmol) and **2a** (42.2 mg, 0.2 mmol) to obtain 98.1 mg (92% yield) compound **3ja** as a pink solid, m.p. 217–219 °C. HPLC (Daicel Chiralpak IA, *n*-hexane/2-propanol = 70:30, flow rate 1.0 mL/min, detection at 254 nm): *t*_R_ = 7.5 min (minor), *t*_R_ = 12.4 min (major); 84% ee. [α]_D_^25^ = +27.4 (*c* = 0.9, CH_2_Cl_2_). ^1^H NMR (400 MHz, DMSO-d_6_): *δ* 8.01 (dd, *J*_1_ = 7.6 Hz, *J*_2_ = 0.8 Hz, 1H), 7.32–7.27 (m, 3H), 7.20–7.15 (m, 3H), 6.88 (d, *J* = 8.0 Hz, 1H), 6.72 (d, *J* = 8.8 Hz, 2H), 4.22 (s, 1H), 3.09 (s, 3H), 3.01 (s, 3H), 2.93 (s, 3H) ppm. ^13^C NMR (176 MHz, DMSO-d_6_): *δ* 176.4, 168.5, 167.8, 158.4, 149.9, 143.0, 131.5, 131.1, 130.5, 129.5, 128.0, 127.3, 122.6, 122.5, 116.3, 108.9, 78.4, 67.8, 63.9, 63.4, 28.7, 28.5, 26.5 ppm. HRMS (ESI): *m*/*z* calcd. for C_25_H_21_^79^BrN_5_O_4_ [M + H]^+^ 534.0771, found 534.0793; calcd. for C_25_H_21_^81^BrN_5_O_4_ [M + H]^+^ 536.0751, found 536.0777.

(2′*R*,3*S*)-4′-Amino-1-benzyl-1″,3″-dimethyl-2,2″,4″,6″-tetraoxo-2′-phenyl-1″,3″,4″,6″-tetrahydro-2″*H*-dispiro[indoline-3,1′-cyclopentane-3′,5″-pyrimidin]-4′-ene-5′-carbonitrile (**3ab**). According to the general procedure from **1a** (58.6 mg, 0.24 mmol) and **2b** (57.4 mg, 0.2 mmol) to obtain 100.9 mg (95% yield) compound **3ab** as a yellow solid, m.p. 165–167 °C. HPLC (Daicel Chiralpak IC, *n*-hexane/2-propanol = 70:30, flow rate 1.0 mL/min, detection at 254 nm): *t*_R_ = 40.3 min (minor), *t*_R_ = 24.9 min (major), >99% ee. [α]_D_^25^ = −11.4 (*c* = 0.4, CH_2_Cl_2_). ^1^H NMR (400 MHz, CDCl_3_): *δ* 8.17 (dd, *J*_1_ = 6.4 Hz, *J*_2_ = 1.4 Hz, 1H), 7.24–7.20 (m, 1H), 7.17–7.00 (m, 7H), 6.82 (d, *J* = 7.6 Hz, 2H), 6.61 (d, *J* = 7.6 Hz, 2H), 6.41 (d, *J* = 7.2 Hz, 1H), 5.60 (s, 2H), 5.02 (d, *J* = 16.4 Hz, 1H), 4.49 (d, *J* = 16.4 Hz, 1H), 4.44 (s, 1H), 3.13 (s, 3H), 2.93 (s, 3H) ppm. ^13^C NMR (101 MHz, CDCl_3_): *δ* 177.2, 168.6, 167.2, 158.2, 149.8, 142.3, 134.4, 130.5, 123.0, 129.55, 129.52, 128.5, 128.30, 128.27, 127.7, 127.2, 126.1, 123.1, 115.5, 109.5, 83.3, 68.8, 66.1, 64.1, 43.8, 29.1, 28.8 ppm. HRMS (ESI): *m*/*z* calcd. for C_31_H_26_N_5_O_4_ [M + H]^+^ 532.1979, found 532.2000.

(2′*R*,3*S*)-4′-Amino-6-chloro-1,1″,3″-trimethyl-2,2″,4″,6″-tetraoxo-2′-phenyl-1″,3″,4″,6″-tetrahydro-2″*H*-dispiro[indoline-3,1′-cyclopentane-3′,5″-pyrimidin]-4′-ene-5′-carbonitrile (**3ac**). According to the general procedure from **1a** (58.6 mg, 0.24 mmol) and **2c** (49.0 mg, 0.2 mmol) to obtain 90.0 mg (92% yield) compound **3ac** as a yellow solid, m.p. 172–174 °C. HPLC (Daicel Chiralpak ADH, *n*-hexane/2-propanol = 70:30, flow rate 1.0 mL/min, detection at 254 nm): *t*_R_ = 8.0 min (minor), *t*_R_ = 15.6 min (major), 91% ee. [α]_D_^25^ = −30.9 (*c* = 0.4, CH_2_Cl_2_). ^1^H NMR (700 MHz, DMSO-d_6_): *δ* 8.04 (d, *J* = 8.4 Hz, 1H), 7.25 (dd, *J*_1_ = 8.0 Hz, *J*_2_ = 2.2 Hz, 3H), 7.19 (t, *J* = 7.4 Hz, 1H), 7.11 (t, *J* = 7.7 Hz, 2H), 7.04 (d, *J* = 1.4 Hz, 1H), 6.75 (d, *J* = 7.7 Hz, 2H), 4.23 (s, 1H), 3.07 (s, 3H), 3.01 (s, 3H), 2.89 (s, 3H) ppm. ^13^C NMR (176 MHz, DMSO-d_6_): *δ* 176.6, 168.5, 167.9, 158.7, 149.8, 144.5, 133.8, 130.8, 129.3, 129.2, 128.6, 128.2, 127.2, 122.1, 116.1, 109.3, 77.9, 67.9, 64.7, 63.2, 28.6, 28.4, 26.6 ppm. HRMS (ESI): *m*/*z* calcd. for C_25_H_21_ClN_5_O_4_ [M + H]^+^ 490.1277, found 490.1300.

(2′*R*,3*S*)-4′-amino-1,1″,3″,5,7-pentamethyl-2,2″,4″,6″-tetraoxo-2′-phenyl-1″,3″,4″,6″-tetrahydro-2″*H*-dispiro[indoline-3,1′-cyclopentane-3′,5″-pyrimidin]-4′-ene-5′-carbonitrile (**3ad**). According to the general procedure from **1a** (58.6 mg, 0.24 mmol) and **2d** (47.8 mg, 0.2 mmol) to obtain 86.9 mg (90% yield) compound **3ad** as a white solid, m.p. 181–182 °C. HPLC (Daicel Chiralpak IC, *n*-hexane/2-propanol = 70:30, flow rate 1.0 mL/min, detection at 254 nm): *t*_R_ = 56.3 min (minor), *t*_R_ = 40.3 min (major), 90% ee. [α]_D_^25^ = −40.6 (*c* = 0.4, CH_2_Cl_2_). ^1^H NMR (700 MHz, DMSO-d_6_): *δ* 7.76 (s, 1H), 7.16 (t, *J* = 7.4 Hz, 1H), 7.13 (s, 2H), 7.07 (t, *J* = 7.7 Hz, 2H), 6.83 (s, 1H), 6.76 (d, *J* = 7.7 Hz, 2H), 4.22 (s, 1H), 3.23 (s, 3H), 3.07 (s, 3H), 2.84 (s, 3H), 2.33 (s, 3H), 2.31 (s, 3H) ppm. ^13^C NMR (176 MHz, DMSO-d_6_): *δ* 177.1, 168.7, 167.8, 158.2, 149.8, 138.5, 133.3, 131.3, 130.8, 129.5, 129.1, 128.9, 128.0, 126.0, 119.3, 116.5, 79.2, 68.2, 65.0, 63.0, 29.6, 28.5, 28.4, 20.7, 18.3 ppm. HRMS (ESI): *m*/*z* calcd. for C_27_H_26_N_5_O_4_ [M + H]^+^ 484.1979, found 484.1992.

(2′*R*,3*S*)-4′-Amino-6-fluoro-1,1″,3″-trimethyl-2,2″,4″,6″-tetraoxo-2′-phenyl-1″,3″,4″,6″-tetrahydro-2″*H*-dispiro[indoline-3,1′-cyclopentane-3′,5″-pyrimidin]-4′-ene-5′-carbonitrile (**3ae**). According to the general procedure from **1a** (58.6 mg, 0.24 mmol) and **2e** (45.8 mg, 0.2 mmol) to obtain 79.5 mg (84% yield) compound **3ae** as a white solid, m.p. 173–175 °C. HPLC (Daicel Chiralpak IC, *n*-hexane/2-propanol = 70:30, flow rate 1.0 mL/min, detection at 254 nm): *t*_R_ = 9.4 min (minor), *t*_R_ = 11.8 min (major), 82% ee. [α]_D_^25^ = −62.8 (*c* = 1, CH_2_Cl_2_). ^1^H NMR (700 MHz, DMSO-d_6_): *δ* 8.04 (dd, *J*_1_ = 7.7 Hz, *J*_2_ = 5.6 Hz, 1H), 7.23 (s, 2H), 7.18 (t, *J* = 7.4 Hz, 1H), 7.10 (t, *J* = 7.4 Hz, 2H), 7.01–6.98 (m, 1H), 6.86 (d, *J* = 9.1 Hz, 1H), 6.75 (d, *J* = 8.4 Hz, 2H), 4.22 (s, 1H), 3.07 (s, 3H), 3.00 (s, 3H), 2.88 (s, 3H) ppm. ^13^C NMR (176 MHz, DMSO-d_6_): *δ* 176.9, 168.6, 167.9, 162.8 (d, ^1^*J*_C–F_ = 243.8 Hz), 158.5, 149.8, 144.8 (d, ^2^*J*_C–F_ = 12.1 Hz), 130.9, 129.4, 129.1, 128.8 (d, ^3^*J*_C–F_ = 9.7 Hz), 128.1, 124.0 (d, ^4^*J*_C–F_ = 2.5 Hz), 116.2, 108.4 (d, ^2^*J*_C–F_ = 22.2 Hz), 97.5 (d, ^2^*J*_C–F_ = 27.6 Hz), 78.1, 68.0, 64.7, 63.1, 28.6, 28.4, 26.7 ppm. ^19^F NMR (659 MHz, DMSO-d_6_): δ −110.9. HRMS (ESI): *m*/*z* calcd. for C_25_H_21_FN_5_O_4_ [M + H]^+^ 474.1572, found 474.1602.

(2′*R*,3*S*)-4′-Amino-6-bromo-1,1″,3″-trimethyl-2,2″,4″,6″-tetraoxo-2′-phenyl-1″,3″,4″,6″-tetrahydro-2″*H*-dispiro[indoline-3,1′-cyclopentane-3′,5″-pyrimidin]-4′-ene-5′-carbonitrile (**3af**). According to the general procedure from **1a** (58.6 mg, 0.24 mmol) and **2f** (57.8 mg, 0.2 mmol) to obtain 101.3 mg (95% yield) compound **3af** as a white solid, m.p. 161–163 °C. HPLC (Daicel Chiralpak IC, *n*-hexane/2-propanol = 70:30, flow rate 1.0 mL/min, detection at 254 nm): *t*_R_ = 17.9 min (major); >99% ee. [α]_D_^25^ = −42.4 (*c* = 0.5, CH_2_Cl_2_). ^1^H NMR (700 MHz, DMSO-d_6_): *δ* 7.97 (d, *J* = 7.7 Hz, 1H), 7.39 (dd, *J*_1_ = 8.0 Hz, *J*_2_ = 1.8 Hz, 1H), 7.25 (s, 2H), 7.19 (t, *J* = 7.4 Hz, 1H), 7.16 (d, *J* = 1.4 Hz, 1H), 7.11 (t, *J* = 7.7 Hz, 2H), 6.75 (d, *J* = 7.7 Hz, 2H), 4.23 (s, 1H), 3.07 (s, 3H), 3.01 (s, 3H), 2.89 (s, 3H) ppm. ^13^C NMR (176 MHz, DMSO-d_6_): *δ* 176.4, 168.5, 167.8, 158.7, 149.8, 144.6, 130.8, 129.3, 129.2, 129.0, 128.2, 127.6, 125.0, 122.2, 116.1, 112.1, 77.9, 67.9, 64.3, 63.2, 28.6, 28.4, 26.6 ppm. HRMS (ESI): *m*/*z* calcd. for C_25_H_21_^79^BrN_5_O_4_ [M + H]^+^ 534.0771, found 534.0785; calcd. for C_25_H_21_^81^BrN_5_O_4_ [M + H]^+^ 536.0751, found 536.0765.

(2′*R*,3*S*)-4′-Amino-5-fluoro-1,1″,3″-trimethyl-2,2″,4″,6″-tetraoxo-2′-phenyl-1″,3″,4″,6″-tetrahydro-2″*H*-dispiro[indoline-3,1′-cyclopentane-3′,5″-pyrimidin]-4′-ene-5′-carbonitrile (**3ag**). According to the general procedure from **1a** (58.6 mg, 0.24 mmol) and **2g** (45.8 mg, 0.2 mmol) to obtain 89.9 mg (95% yield) compound **3ag** as a white solid, m.p. 201–203 °C. HPLC (Daicel Chiralpak ADH, *n*-hexane/2-propanol = 70:30, flow rate 1.0 mL/min, detection at 254 nm): *t*_R_ = 18.7 min (minor), *t*_R_ = 20.2 min (major); 95% ee. [α]_D_^25^ = −58.6 (*c* = 0.5, CH_2_Cl_2_). ^1^H NMR (700 MHz, DMSO-d_6_): *δ* 7.91 (dd, *J*_1_ = 8.8 Hz, *J*_2_ = 2.4 Hz, 1H), 7.28 (s, 2H), 7.20–7.15 (m, 2H), 7.12 (t, *J* = 7.7 Hz, 2H), 6.90 (dd, *J*_1_ = 8.4 Hz, *J*_2_ = 4.2 Hz, 1H), 6.76 (d, *J* = 7.7 Hz, 2H), 4.26 (s, 1H), 3.07 (s, 3H), 3.00 (s, 3H), 2.91 (s, 3H) ppm. ^13^C NMR (176 MHz, DMSO-d_6_): *δ* 176.2, 168.5, 168.0, 158.8, 158.3 (^1^*J*_C–F_ = 237.4 Hz), 149.8, 139.4, 130.8, 130.2 (^3^*J*_C–F_ = 8.3 Hz), 129.23, 129.19, 128.3, 116.1, 115.7 (^2^*J*_C–F_ = 23.4 Hz), 114.8 (^2^*J*_C–F_ = 25.7 Hz), 109.8 (^3^*J*_C–F_ = 8.3 Hz), 78.1, 67.9, 64.6, 63.8, 29.9, 28.6, 28.4, 26.6 ppm. ^19^F NMR (659 MHz, DMSO-d_6_) δ −120.1. HRMS (ESI): *m*/*z* calcd. for C_25_H_21_FN_5_O_4_ [M + H]^+^ 474.1572, found 474.1595.

(2′*R*,3*S*)-4′-Amino-5-bromo-1,1″,3″-trimethyl-2,2″,4″,6″-tetraoxo-2′-phenyl-1″,3″,4″,6″-tetrahydro-2″*H*-dispiro[indoline-3,1′-cyclopentane-3′,5″-pyrimidin]-4′-ene-5′-carbonitrile (**3ah**). According to the general procedure from **1a** (58.6 mg, 0.24 mmol) and **2h** (57.8 mg, 0.2 mmol) to obtain 101.3 mg (95% yield) compound **3ah** as a white solid, m.p. 192–194 °C. HPLC (Daicel Chiralpak IA, *n*-hexane/2-propanol = 80:20, flow rate 1.0 mL/min, detection at 254 nm): *t*_R_ = 14.1 min (minor), *t*_R_ = 11.3 min (major), >99% ee. [α]_D_^25^ = −211.6 (*c* = 0.8, CH_2_Cl_2_). ^1^H NMR (700 MHz, DMSO-d_6_): *δ* 8.23 (d, *J* = 2.1 Hz, 1H), 7.50 (dd, *J*_1_ = 8.4 Hz, *J*_2_ = 2.1 Hz, 1H), 7.30 (s, 2H), 7.19 (t, *J* = 7.4 Hz, 1H), 7.12 (t, *J* = 7.7 Hz, 2H), 6.87 (d, *J* = 8.4 Hz, 1H), 6.75 (d, *J* = 7.0 Hz, 2H), 4.24 (s, 1H), 3.07 (s, 3H), 2.99 (s, 3H), 2.90 (s, 3H) ppm. ^13^C NMR (176 MHz, DMSO-d_6_): *δ* 176.0, 168.4, 167.9, 158.8, 149.7, 142.3, 132.0, 130.8, 130.7, 130.0, 129.23, 129.18, 128.3, 116.1, 114.2, 110.8, 77.9, 67.8, 64.6, 63.5, 28.6, 28.4, 26.6 ppm. HRMS (ESI): *m*/*z* calcd. for C_25_H_21_^79^BrN_5_O_4_ [M + H]^+^ 534.0771, found 534.0786; calcd. for C_25_H_21_^81^BrN_5_O_4_ [M + H]^+^ 536.0751, found 536.0769.

(2′*R*,3*S*)-4′-Amino-5-chloro-1,1″,3″-trimethyl-2,2″,4″,6″-tetraoxo-2′-phenyl-1″,3″,4″,6″-tetrahydro-2″*H*-dispiro[indoline-3,1′-cyclopentane-3′,5″-pyrimidin]-4′-ene-5′-carbonitrile (**3ai**). According to the general procedure from **1a** (58.6 mg, 0.24 mmol) and **2i** (49.0 mg, 0.2 mmol) to obtain 90.0 mg (92% yield) compound **3ai** as a white solid, m.p. 201–204 °C. HPLC (Daicel Chiralpak IA, *n*-hexane/2-propanol = 90:10, flow rate 1.0 mL/min, detection at 254 nm): *t*_R_ = 45.7 min (minor), *t*_R_ = 32.5 min (major); >99% ee. [α]_D_^25^ = −100.6 (*c* = 0.5, CH_2_Cl_2_). ^1^H NMR (700 MHz, DMSO-d_6_): *δ* 8.11 (d, *J* = 2.8 Hz, 1H), 7.37 (dd, *J*_1_ = 8.4 Hz, *J*_2_ = 2.8 Hz, 1H), 7.30 (s, 2H), 7.19 (t, *J* = 7.7 Hz, 1H), 7.12 (t, *J* = 7.7 Hz, 2H), 6.92 (d, *J* = 7.7 Hz, 1H), 6.75 (dd, *J*_1_ = 8.4 Hz, *J*_2_ = 1.4 Hz, 2H), 4.25 (s, 1H), 3.08 (s, 3H), 3.00 (s, 3H), 2.91 (s, 3H) ppm. ^13^C NMR (176 MHz, DMSO-d_6_): *δ* 176.1, 168.4, 168.0, 158.8, 149.74, 142.0, 130.7, 130.4, 129.23, 129.19, 128.3, 127.2, 126.5, 116.1, 110.4, 77.9, 67.8, 64.6, 63.6, 28.6, 28.4, 26.6 ppm. HRMS (ESI): *m*/*z* calcd. for C_25_H_21_ClN_5_O_4_ [M + H]^+^ 490.1277, found 490.1304.

(2′*R*,3*S*)-4′-Amino-1,1″,3″-trimethyl-2,2″,4″,6″-tetraoxo-2′-phenyl-7-(trifluoromethyl)-1″,3″,4″,6″-tetrahydro-2″*H*-dispiro[indoline-3,1′-cyclopentane-3′,5″-pyrimidin]-4′-ene-5′-carbonitrile (**3aj**). According to the general procedure from **1a** (58.6 mg, 0.24 mmol) and **2j** (55.8 mg, 0.2 mmol) to obtain 88.9 mg (85% yield) compound **3aj** as a white solid, m.p. 177–179 °C. HPLC (Daicel Chiralpak IA, *n*-hexane/2-propanol = 70:30, flow rate 1.0 mL/min, detection at 254 nm): *t*_R_ = 5.6 min (minor), *t*_R_ = 7.6 min (major); 83% ee. [α]_D_^25^ = −60.8 (*c* = 0.5, CH_2_Cl_2_). ^1^H NMR (700 MHz, DMSO-d_6_): *δ* 8.37 (d, *J* = 7.0 Hz, 1H), 7.64 (dd, *J*_1_ = 8.0 Hz, *J*_2_ = 1.0 Hz, 1H), 7.38 (t, *J* = 8.0 Hz, 3H), 7.17 (t, *J* = 7.4 Hz, 1H), 7.07 (t, *J* = 7.7 Hz, 2H), 6.67 (d, *J* = 7.0 Hz, 2H), 4.22 (s, 1H), 3.14 (s, 3H), 3.08 (s, 3H), 2.89 (s, 3H) ppm. ^13^C NMR (176 MHz, DMSO-d_6_): *δ* 178.0, 168.5, 167.8, 159.3, 149.8, 140.6, 131.4, 131.3, 130.4, 129.2, 128.1, 127.3, 127.2 (q, ^3^*J*_C–F_ = 5.5 Hz), 123.2 (q, ^1^*J*_C–F_ = 271.2 Hz), 122.2, 116.0, 110.9 (q, ^2^*J*_C–F_ = 32.7 Hz), 77.2, 67.8, 65.3, 62.2, 29.0 (q, *J*_C–F_ = 5.8 Hz), 28.6, 28.4 ppm. ^19^F NMR (659 MHz, DMSO-d_6_): δ −52.1. HRMS (ESI): *m*/*z* calcd. for C_26_H_21_F_3_N_5_O_4_ [M + H]^+^ 524.1540, found 524.1559.

(2′*R*,3*S*)-4′-Amino-1,1″,3″,7-tetramethyl-2,2″,4″,6″-tetraoxo-2′-phenyl-1″,3″,4″,6″-tetrahydro-2″*H*-dispiro[indoline-3,1′-cyclopentane-3′,5″-pyrimidin]-4′-ene-5′-carbonitrile (**3ak**). According to the general procedure from **1a** (58.6 mg, 0.24 mmol) and **2k** (45.0 mg, 0.2 mmol) to obtain 89.1 mg (95% yield) compound **3ak** as a white solid, m.p. 182–185 °C. HPLC (Daicel Chiralpak IA, *n*-hexane/2-propanol = 70:30, flow rate 1.0 mL/min, detection at 254 nm): *t*_R_ = 7.5 min (minor), *t*_R_ = 9.3 min (major); 82% ee. [α]_D_^25^ = 23.4 (*c* = 0.4, CH_2_Cl_2_). ^1^H NMR (700 MHz, DMSO-d_6_): *δ* 7.92 (d, *J* = 6.3 Hz, 1H), 7.18–7.14 (m, 3H), 7.08–7.01 (m, 3H), 6.75 (d, *J* = 7.7 Hz, 2H), 4.23 (s, 1H), 3.27 (s, 3H), 3.07 (s, 3H), 2.84 (s, 3H), 2.38 (s, 3H) ppm. ^13^C NMR (176 MHz, DMSO-d_6_): *δ* 177.2, 168.7, 167.8, 158.3, 149.8, 140.8, 132.9, 131.2, 129.5, 129.0, 128.0, 125.4, 122.1, 119.7, 116.4, 79.0, 68.2, 65.0, 62.9, 29.6, 28.5, 28.3, 18.4 ppm. HRMS (ESI): *m*/*z* calcd. for C_26_H_24_N_5_O_4_ [M + H]^+^ 470.1823, found 470.1834.

### 3.5. Gram-Scale Synthesis of 3aa

To a dried 50 mL round-bottom flask, benzylidene barbituric acid **1a** (1.17 g, 4.8 mmol), oxindolylmalonitrile **2a** (0.84 g, 4.0 mmol), chiral organocatalyst **C5** (101.6 mg, 0.2 mmol, 0.05 equiv), and CH_2_Cl_2_ (20 mL) were added. After stirring at room temperature for 8 h, the reaction mixture was concentrated and directly purified by silica gel column chromatography (200–300 mesh) using ethyl acetate/petroleum ether (1:2) as eluent to afford the desired product **3aa** (1.6 g, 88% yield).

## 4. Conclusions

In summary, we developed an efficient and practical asymmetric Michael/cyclization reaction of benzylidene barbituric acids with oxindolylmalonitriles at room temperature. Using a squaramide catalyst, a series of chiral bisspiro barbituric acid derivatives were obtained in high yields (72–97%) with high-to-excellent stereoselectivities (up to >99% ee and >20:1 dr). In addition, the practicability of the reaction was verified by the preparation of the product at the gram-scale.

## Figures and Tables

**Table 1:** Optimization of reaction conditions ^a.^.

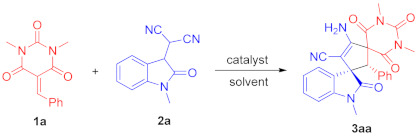					
Entry ^a^	Solvent	Catalyst	Yield ^b^ (%)	dr ^c^	ee ^d^ (%)
1	CH_2_Cl_2_	**C1**	86	>20:1	88
2	CH_2_Cl_2_	**C2**	77	>20:1	65
3	CH_2_Cl_2_	**C3**	82	>20:1	50
4	CH_2_Cl_2_	**C4**	85	>20:1	83
5	CH_2_Cl_2_	**C5**	94	>20:1	>99
6	CH_2_Cl_2_	**C6**	83	>20:1	82
7	CH_2_Cl_2_	**C7**	75	>20:1	87
8	CH_2_Cl_2_	**C8**	88	>20:1	85
9	CH_2_Cl_2_	**C9**	92	>20:1	53
10	CH_2_Cl_2_	**C10**	85	>20:1	81
11	CH_2_Cl_2_	**C11**	80	>20:1	82
12	CH_2_Cl_2_	**C12**	77	>20:1	74
13	MeCN	**C5**	68	>20:1	25
14	PhMe	**C5**	72	>20:1	80
15	THF	**C5**	72	>20:1	47
16	CHCl_3_	**C5**	92	>20:1	97
17	MTBE	**C5**	57	>20:1	5
18 ^e^	CH_2_Cl_2_	**C5**	80	>20:1	88
19 ^f^	CH_2_Cl_2_	**C5**	86	>20:1	94

^a^ Unless otherwise specified, reactions were conducted with **1a** (0.24 mmol), **2a** (0.20 mmol), and catalyst (10 mol%) in solvent (1.5 mL) at room temperature under air for 8 h. ^b^ Isolated yield after column chromatography purification. ^c^ Determined by ^1^H NMR analysis. ^d^ Enantiomeric excess (ee) was determined by HPLC analysis. ^e^ 5 mol% catalyst was used. ^f^ The reaction was performed at 0 °C for 24 h.

## Data Availability

Data are contained within this article and Supplementary Materials.

## Supplementary Materials

The following supporting information can be downloaded at https://www.mdpi.com/article/10.3390/molecules30092000/s1, Copies of ^1^H and ^13^C NMR spectra, HPLC chromatograms for all new compounds.
