# Supplementary material for: Squaramide-Catalyzed Asymmetric Michael Addition/Cyclization Reaction for the Synthesis of Chiral Bisspiro Barbituric Acid–Oxindole Derivatives
Source: Molecules. 2025 Apr 30;30(9):2000. doi: 10.3390/molecules30092000 (PMC12073245; doi:10.3390/molecules30092000)
Supplement: Supplementary file 1 [file molecules-30-02000-s001.zip › molecules-3582442-supplementary.pdf]

Squaramide-Catalyzed Asymmetric Michael Addition/Cyclization Reaction for the Synthesis of Chiral Bisspiro Barbituric Acid-Oxindoles

De-Jun Qiao, Da-Ming Du*

Key Laboratory of Medicinal Molecule Science and Pharmaceutical Engineering, School of Chemistry and Chemical Engineering, Beijing Institute of Technology, Beijing 100081, China

E-mail: dudm@bit.edu.cn

Supporting Information

Contents

1. Copies of ^{1}H and ^{13}C NMR spectra of new compounds……….......................................S1
2. Copies of HPLC chromatograms……………………………………………..…....S23

1. Copies of ^{1}H, ^{13}C and ^{19}F NMR spectra of new compounds

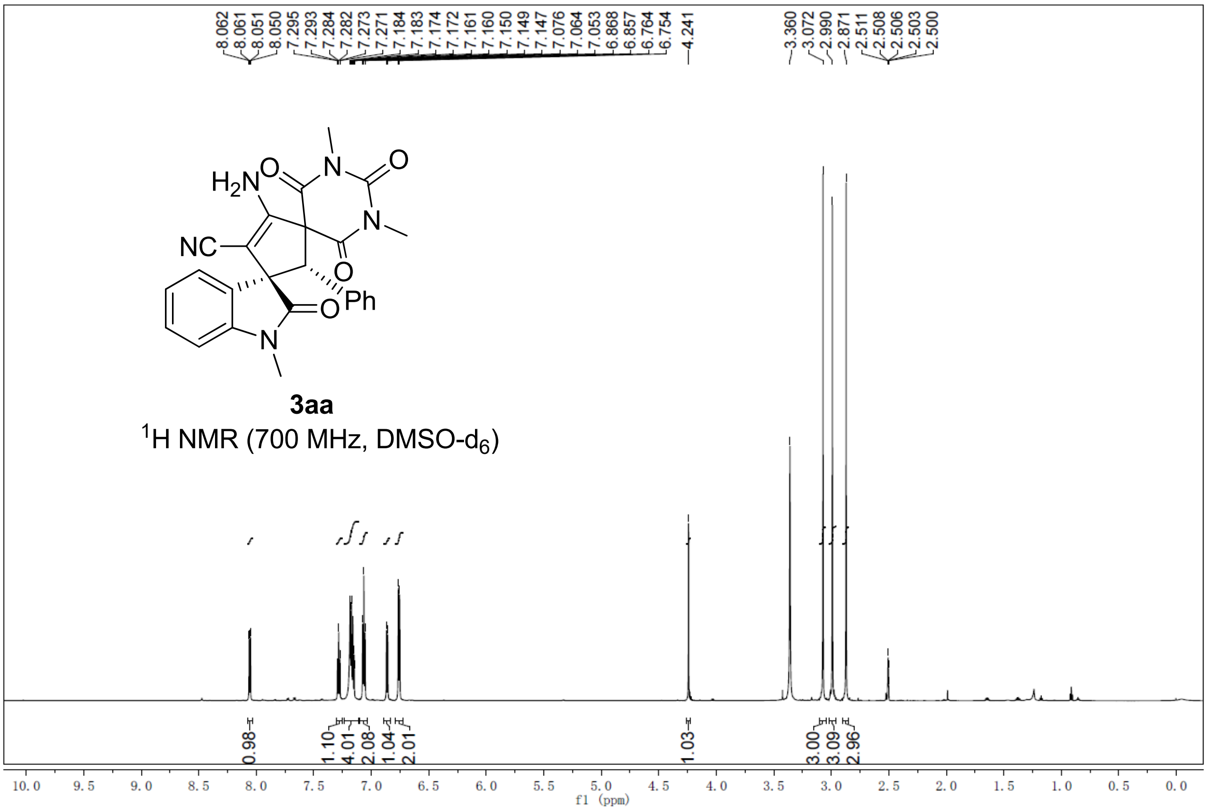

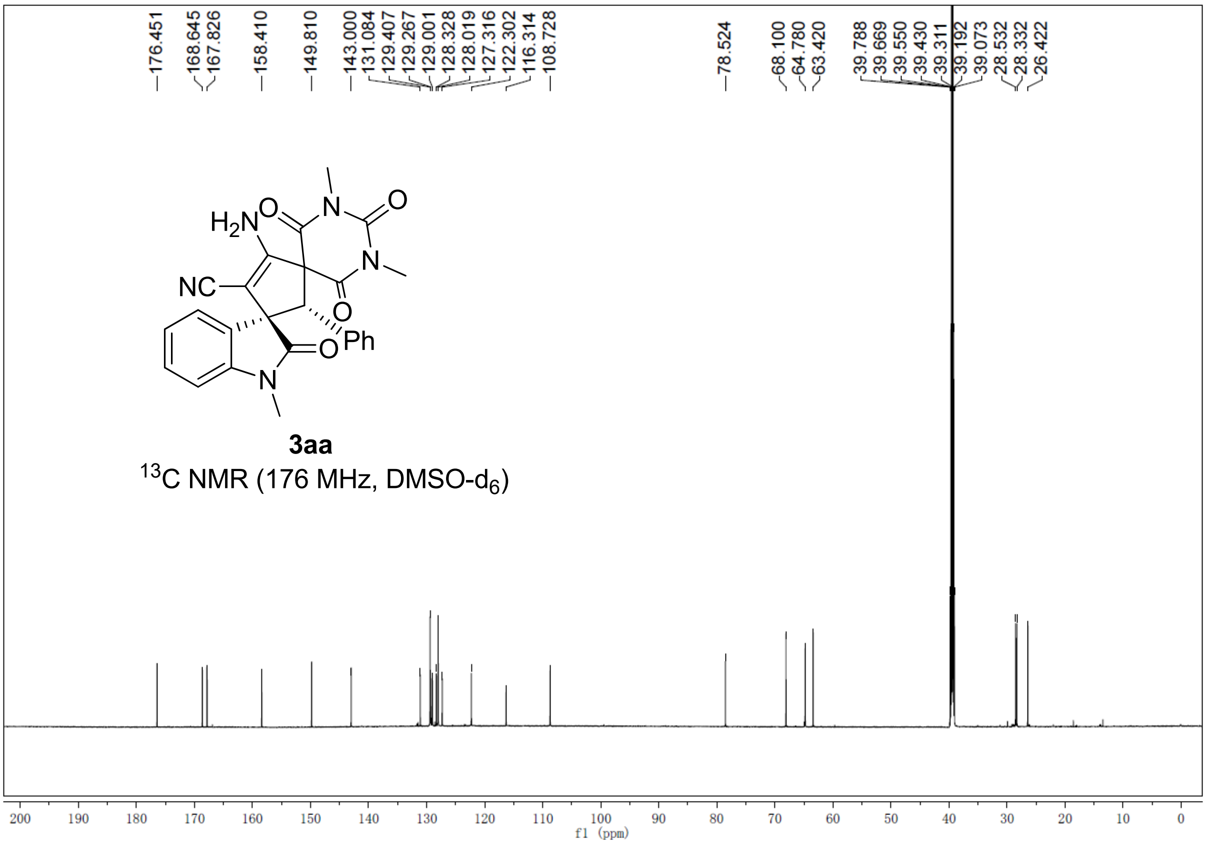

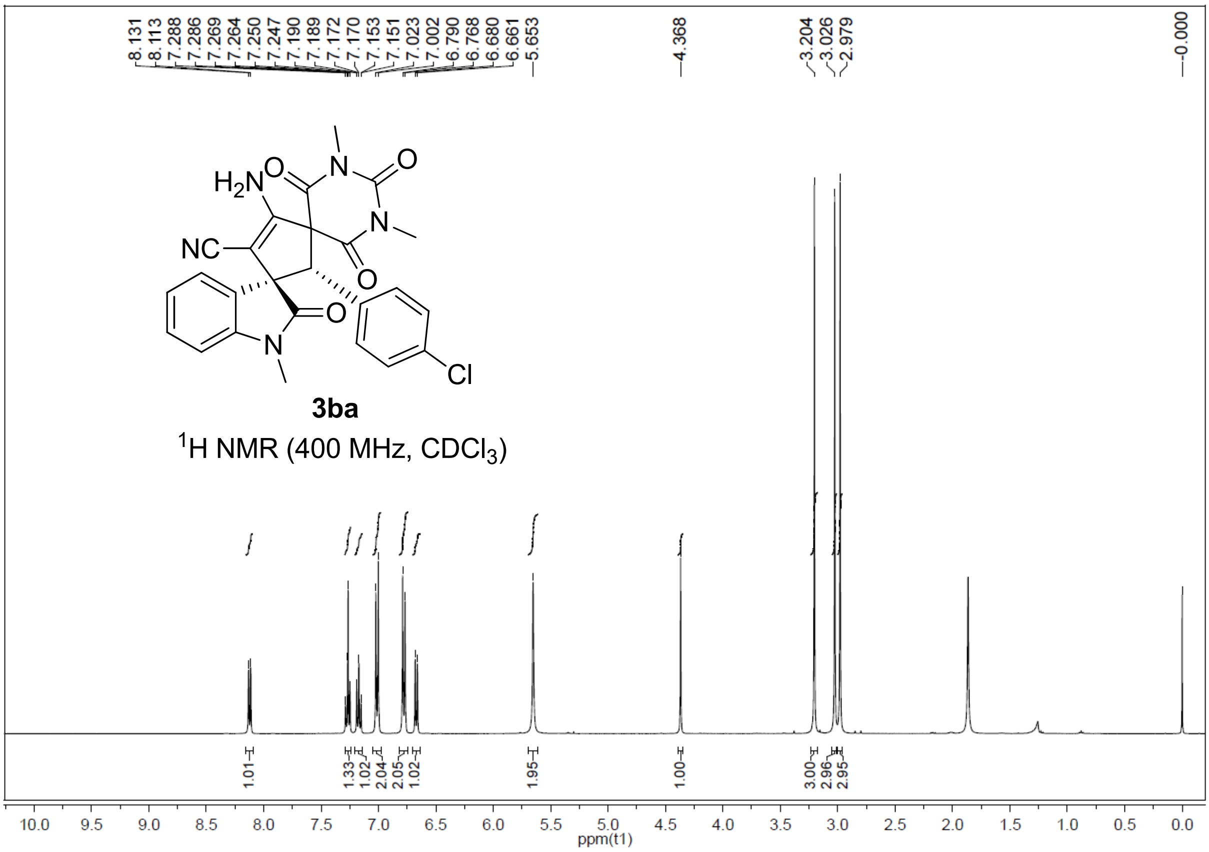
8.131
8.113
7.288
7.286
7.269
7.264
7.250
7.247
7.190
7.189
7.172
7.170
7.153
7.151
7.023
7.002
6.790
6.768
6.680
6.661
5.653
4.368
3.204
3.026
2.979
0.000
H2N
NC
O
N
Cl
3ba
1H NMR (400 MHz, CDCl3)
1.01
1.33
1.02
2.04
2.05
1.02
1.95
1.00
3.00
2.96
2.95
ppm(t1)

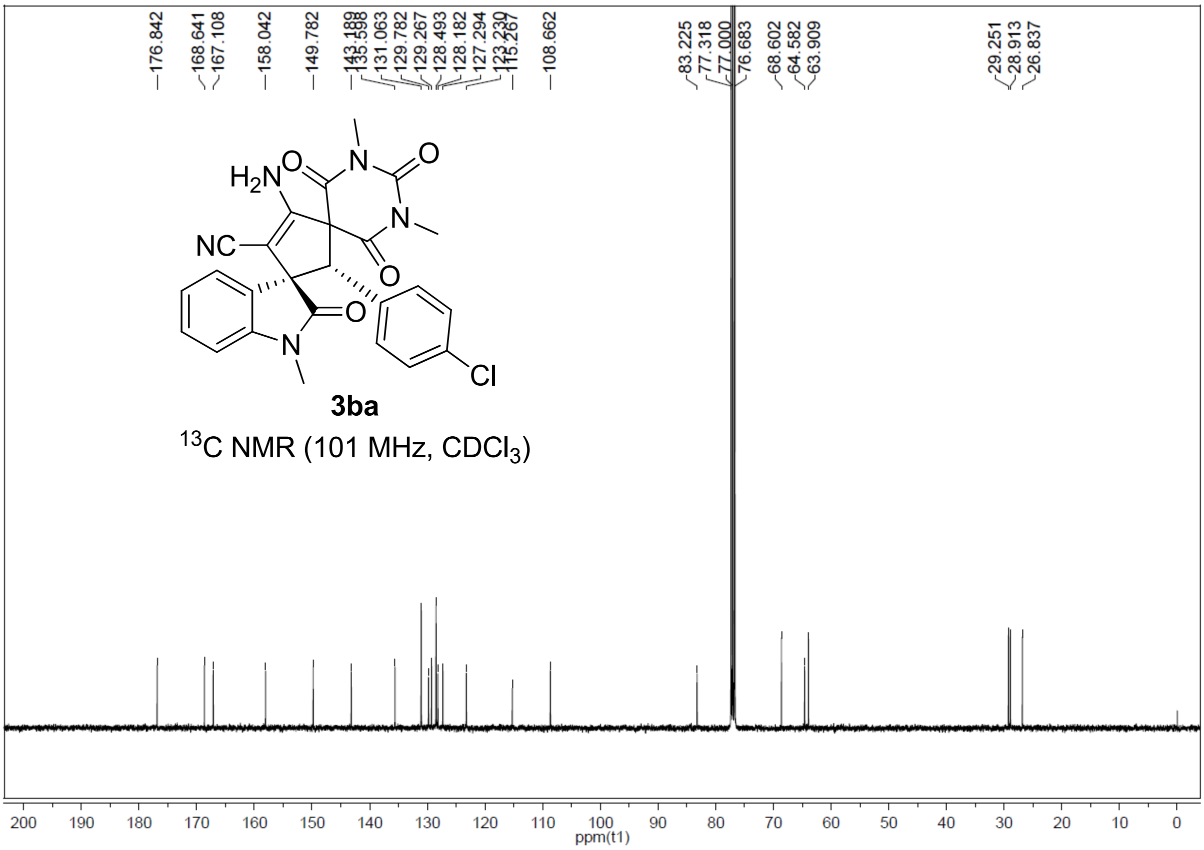
176.842
168.641
167.108
158.042
149.782
143.189
135.598
131.063
129.782
129.267
128.493
128.182
127.294
123.230
115.267
108.662
83.225
77.318
77.000
76.683
68.602
64.582
63.909
29.251
28.913
26.837
H2N
NC
O
N
Cl
3ba
13C NMR (101 MHz, CDCl3)
ppm(t1)

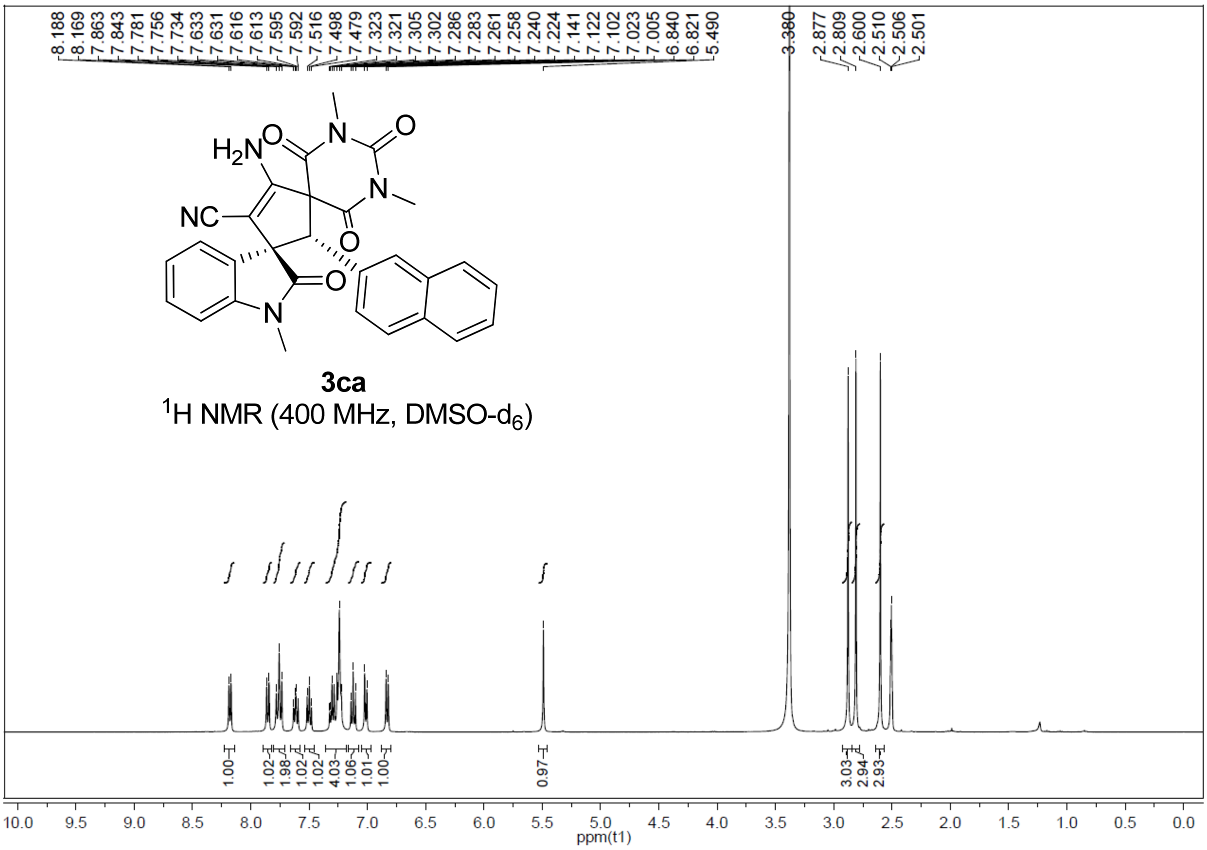
3ca
^{1}H NMR (400 MHz, DMSO-d$_6$)
ppm(t1)

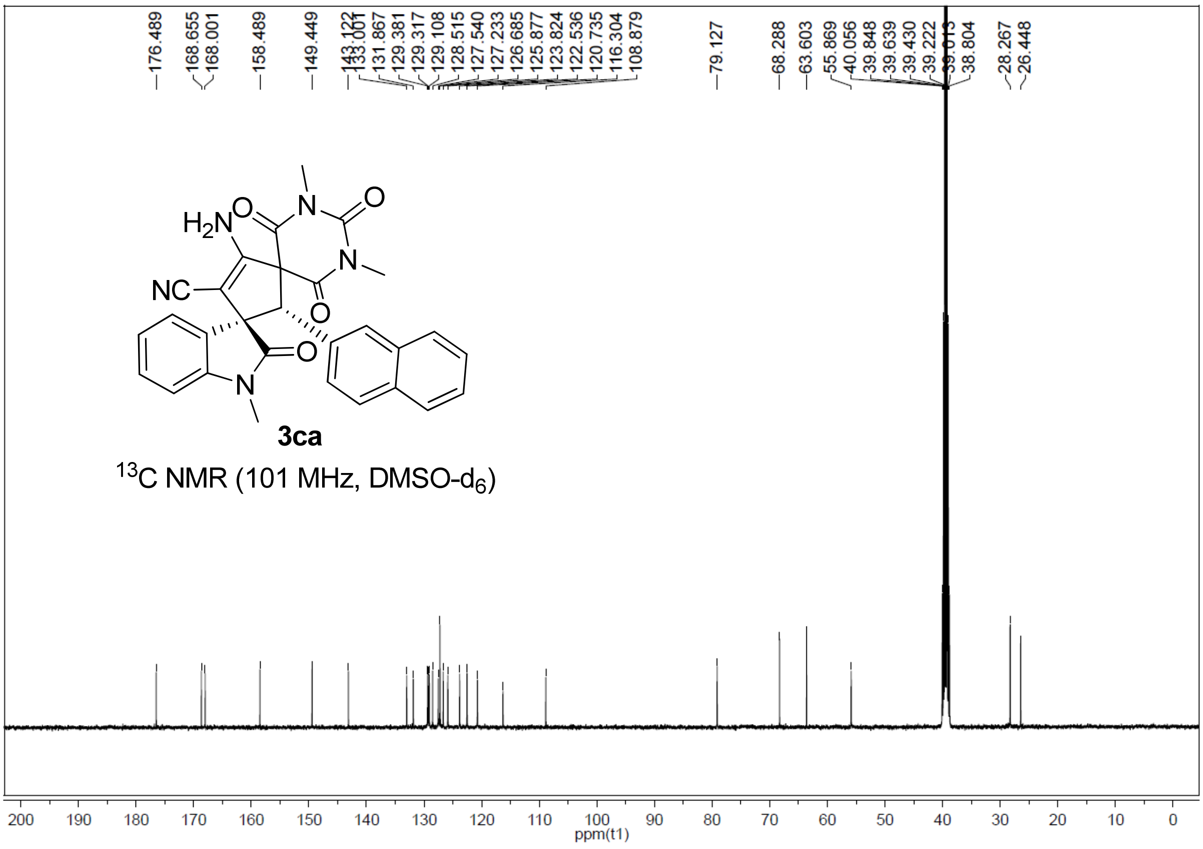
3ca
^{13}C NMR (101 MHz, DMSO-d$_6$)
ppm(t1)

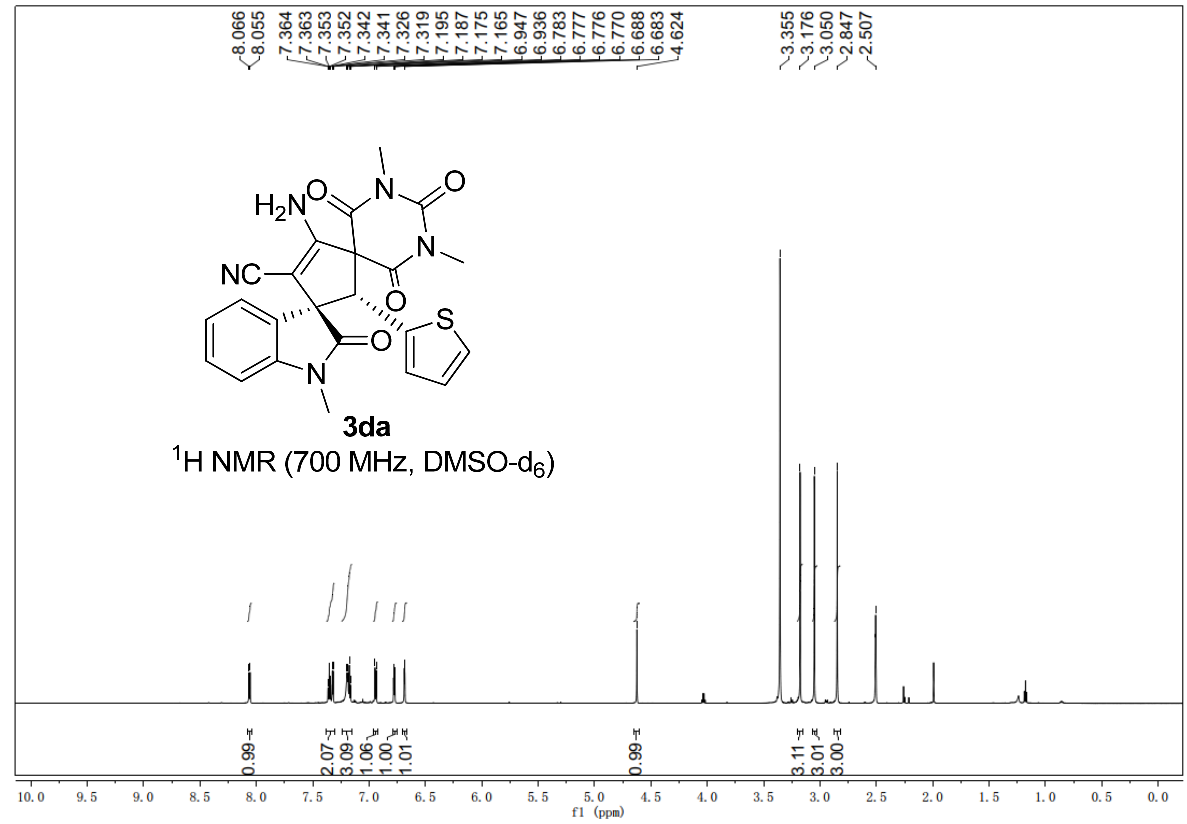

8.066
8.055
7.364
7.363
7.353
7.352
7.342
7.341
7.326
7.319
7.195
7.187
7.175
7.165
6.947
6.936
6.783
6.777
6.776
6.770
6.688
6.683
4.624
3.355
3.176
3.050
2.847
2.507
H2N
O
N
O
N
NC
O
O
S
O
N
3da
1H NMR (700 MHz, DMSO-d6)
0.99
2.07
3.09
1.06
1.00
1.01
0.99
3.11
3.01
3.00
f1 (ppm)

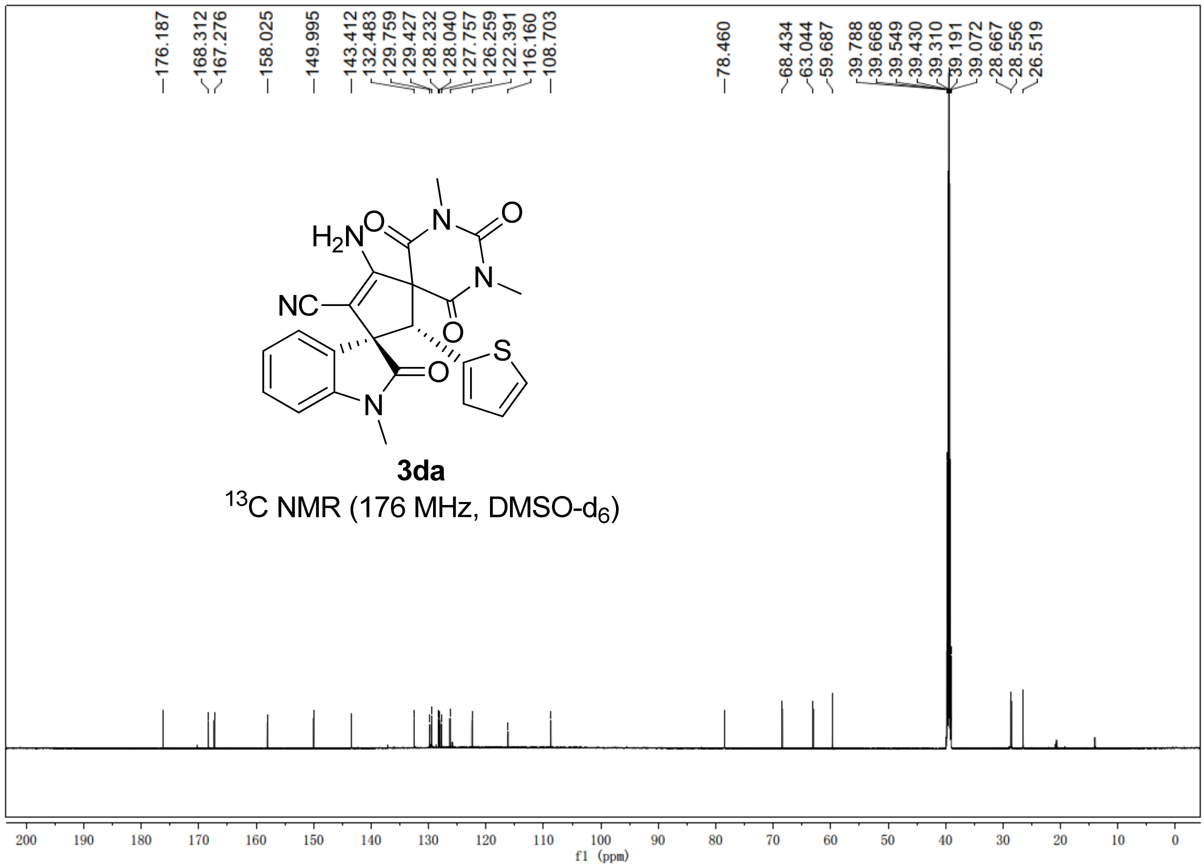

176.187
168.312
167.276
158.025
149.995
143.412
132.483
129.759
129.427
128.232
128.040
127.757
126.259
122.391
116.160
108.703
78.460
68.434
63.044
59.687
39.788
39.668
39.549
39.430
39.310
39.191
39.072
28.667
28.556
26.519
H2N
O
N
O
N
NC
O
O
S
O
N
3da
13C NMR (176 MHz, DMSO-d6)
f1 (ppm)

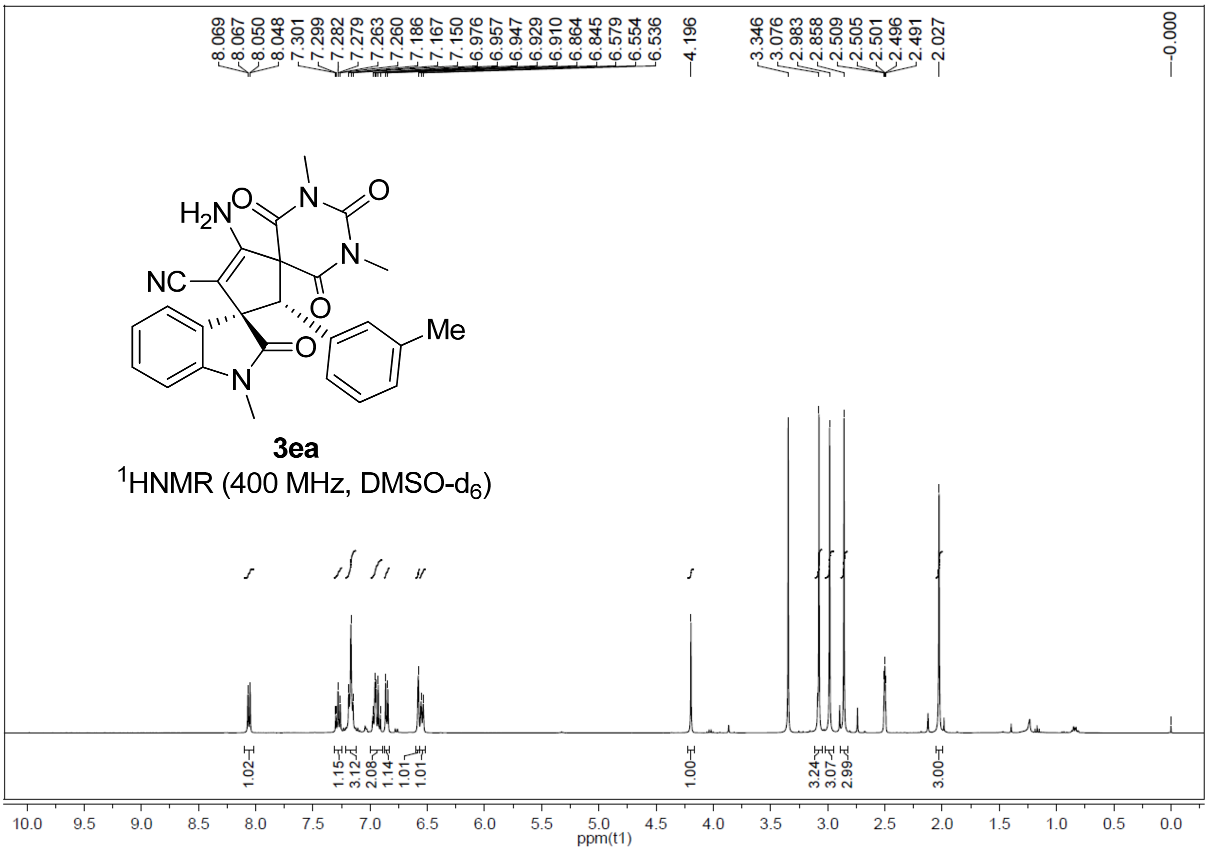
8.069
8.067
8.050
8.048
7.301
7.299
7.282
7.279
7.263
7.260
7.186
7.167
7.150
6.976
6.957
6.947
6.929
6.910
6.864
6.845
6.579
6.554
6.536
4.196
3.346
3.076
2.983
2.858
2.509
2.505
2.501
2.496
2.491
2.027
0.000
H2N
O
N
O
N
NC
O
O
Me
N
3ea
1HNMR (400 MHz, DMSO-d6)
1.02
1.15
3.12
2.08
1.14
1.01
1.01
1.00
3.24
3.07
2.99
3.00
10.0 9.5 9.0 8.5 8.0 7.5 7.0 6.5 6.0 5.5 5.0 4.5 4.0 3.5 3.0 2.5 2.0 1.5 1.0 0.5 0.0
ppm(t1)

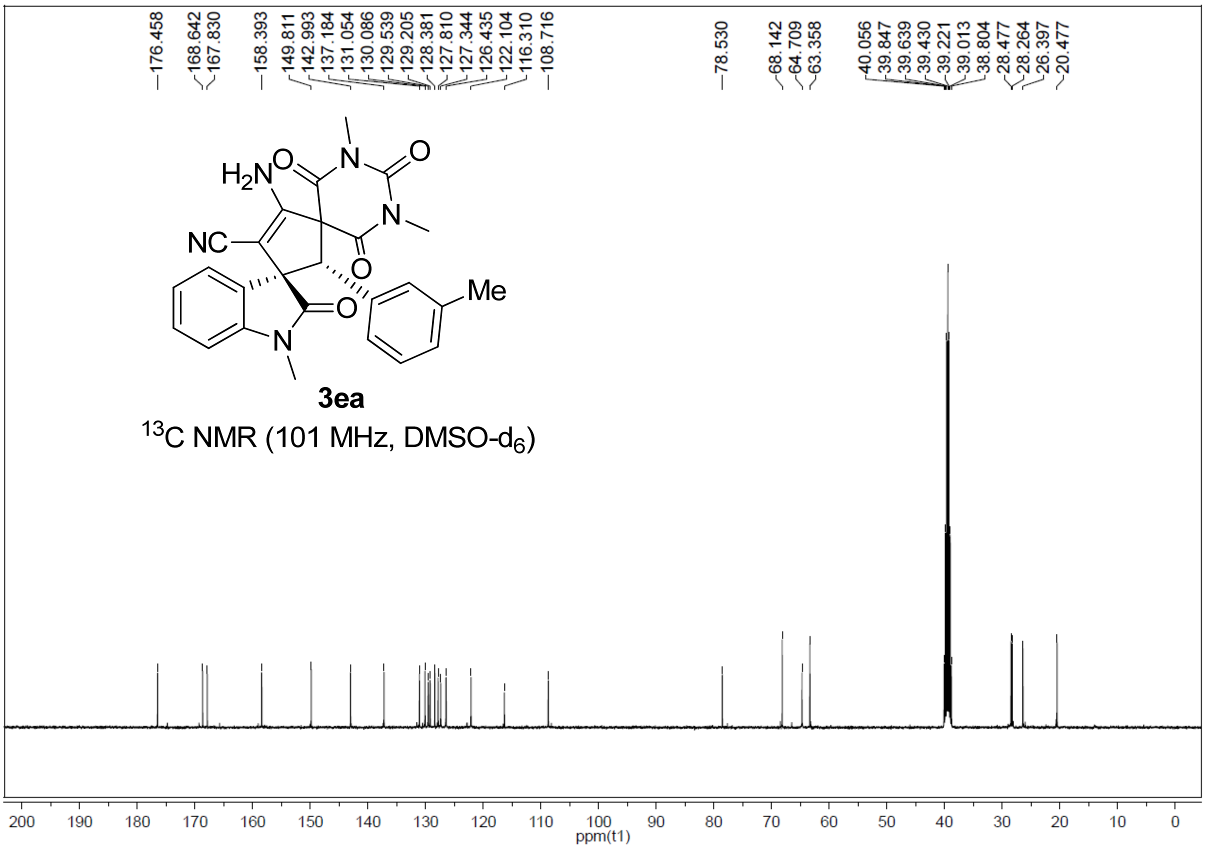
176.458
168.642
167.830
158.393
149.811
142.993
137.184
131.054
130.086
129.539
129.205
128.381
127.810
127.344
126.435
122.104
116.310
108.716
78.530
68.142
64.709
63.358
40.056
39.847
39.639
39.430
39.221
39.013
38.804
28.477
28.264
26.397
20.477
H2N
O
N
O
N
NC
O
O
Me
N
3ea
13C NMR (101 MHz, DMSO-d6)
200 190 180 170 160 150 140 130 120 110 100 90 80 70 60 50 40 30 20 10 0
ppm(t1)

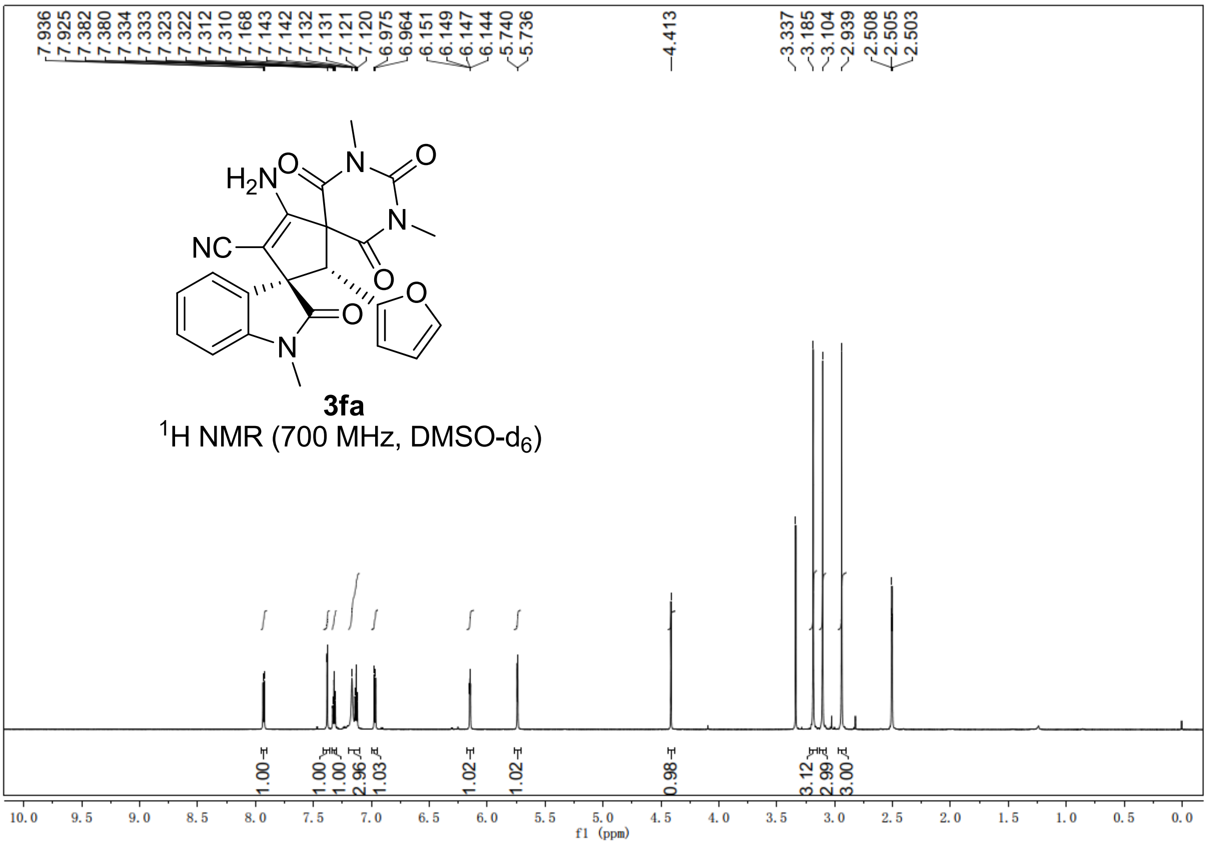
7.936
7.925
7.382
7.380
7.334
7.333
7.323
7.322
7.312
7.310
7.168
7.143
7.142
7.132
7.131
7.121
7.120
6.975
6.964
6.151
6.149
6.147
6.144
5.740
5.736
4.413
3.337
3.185
3.104
2.939
2.508
2.505
2.503
H2N
O
N
O
N
NC
O
O
O
O
N
3fa
1H NMR (700 MHz, DMSO-d6)
1.00
1.00
1.00
2.96
1.03
1.02
1.02
0.98
3.12
2.99
3.00
f1 (ppm)

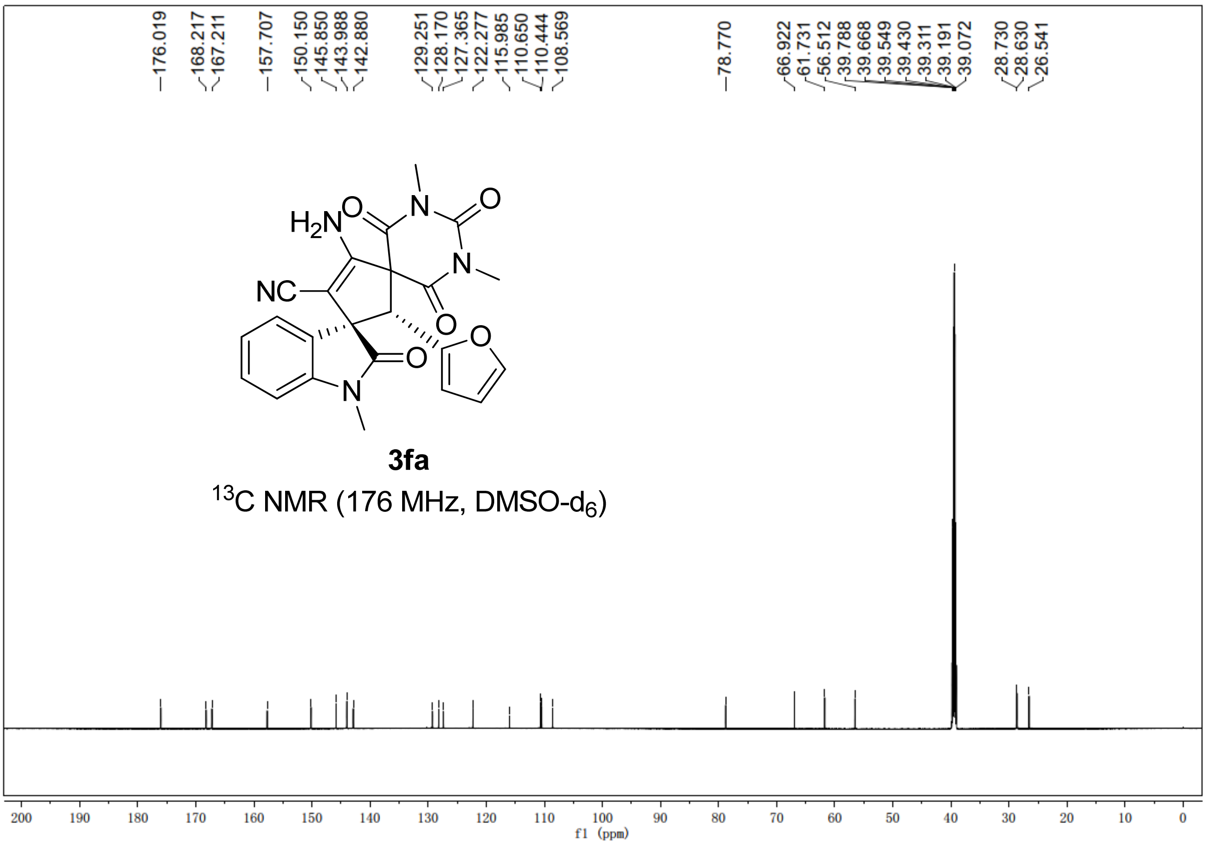
176.019
168.217
167.211
157.707
150.150
145.850
143.988
142.880
129.251
128.170
127.365
122.277
115.985
110.650
110.444
108.569
78.770
66.922
61.731
56.512
39.788
39.668
39.549
39.430
39.311
39.191
39.072
28.730
28.630
26.541
H2N
O
N
O
N
NC
O
O
O
O
N
3fa
13C NMR (176 MHz, DMSO-d6)
f1 (ppm)

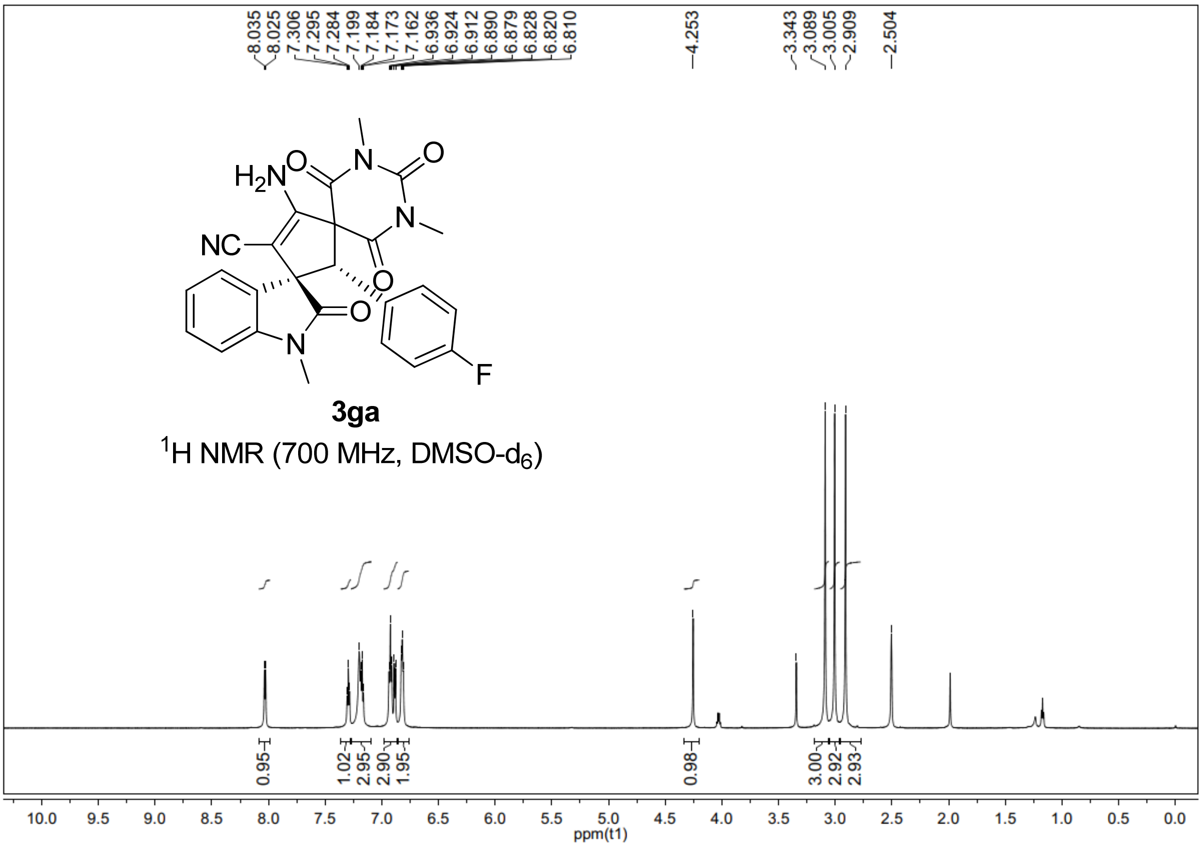
8.035
8.025
7.306
7.295
7.284
7.199
7.184
7.173
7.162
6.936
6.924
6.912
6.890
6.879
6.828
6.820
6.810
4.253
3.343
3.089
3.005
2.909
2.504
H2N
O
N
O
N
O
NC
O
O
N
F
3ga
1H NMR (700 MHz, DMSO-d6)
0.95
1.02
2.95
2.90
1.95
0.98
3.00
2.92
2.93
10.0
9.5
9.0
8.5
8.0
7.5
7.0
6.5
6.0
5.5
5.0
4.5
4.0
3.5
3.0
2.5
2.0
1.5
1.0
0.5
0.0
ppm(t1)

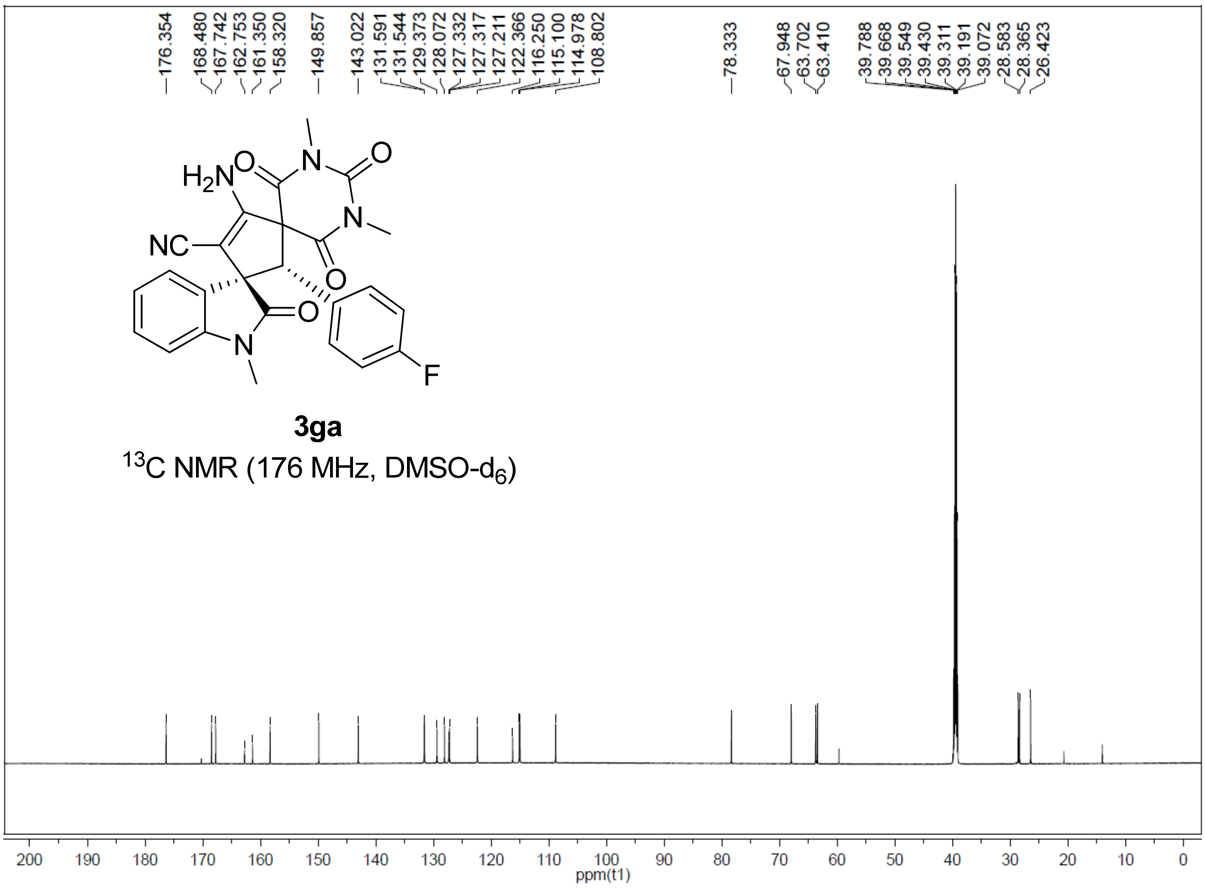
176.354
168.480
167.742
162.753
161.350
158.320
149.857
143.022
131.591
131.544
129.373
128.072
127.332
127.317
127.211
122.366
116.250
115.100
114.978
108.802
78.333
67.948
63.702
63.410
39.788
39.668
39.549
39.430
39.311
39.191
39.072
28.583
28.365
26.423
H2N
O
N
O
N
O
NC
O
O
N
F
3ga
13C NMR (176 MHz, DMSO-d6)
200
190
180
170
160
150
140
130
120
110
100
90
80
70
60
50
40
30
20
10
0
ppm(t1)

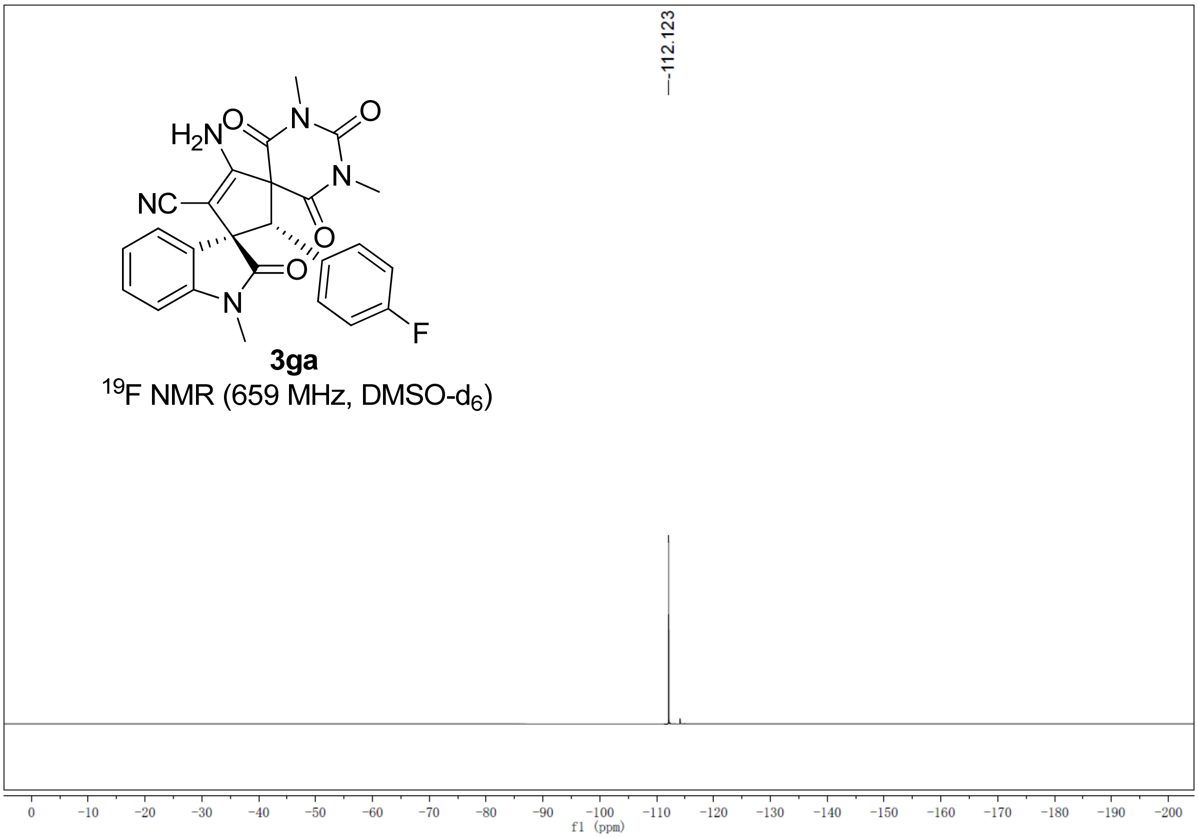

-112.123
3ga
^{19}F NMR (659 MHz, DMSO-d$_6$)
f1 (ppm)

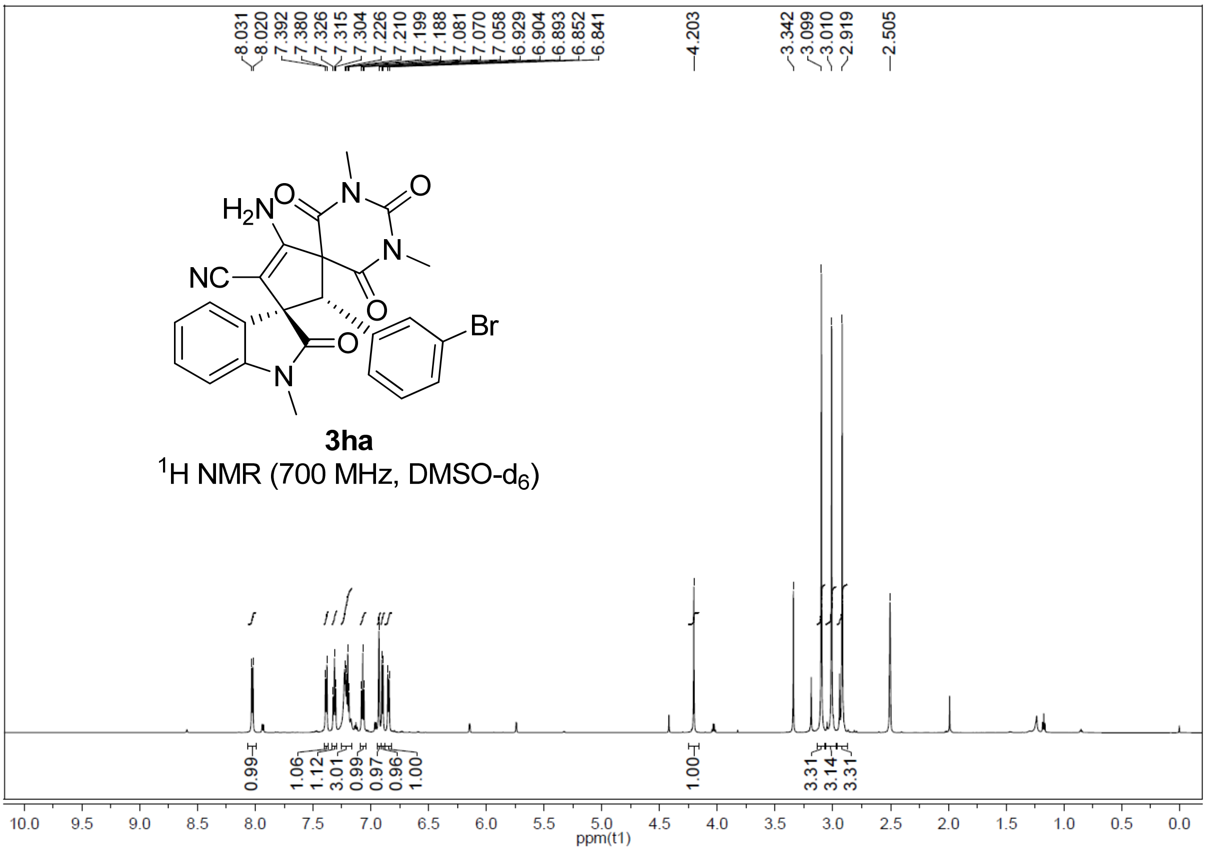

8.031
8.020
7.392
7.380
7.326
7.315
7.304
7.226
7.210
7.199
7.188
7.081
7.070
7.058
6.929
6.904
6.893
6.852
6.841
4.203
3.342
3.099
3.010
2.919
2.505
3ha
^{1}H NMR (700 MHz, DMSO-d$_6$)
0.99
1.06
1.12
3.01
0.99
0.97
0.96
1.00
1.00
3.31
3.14
3.31
ppm(t1)

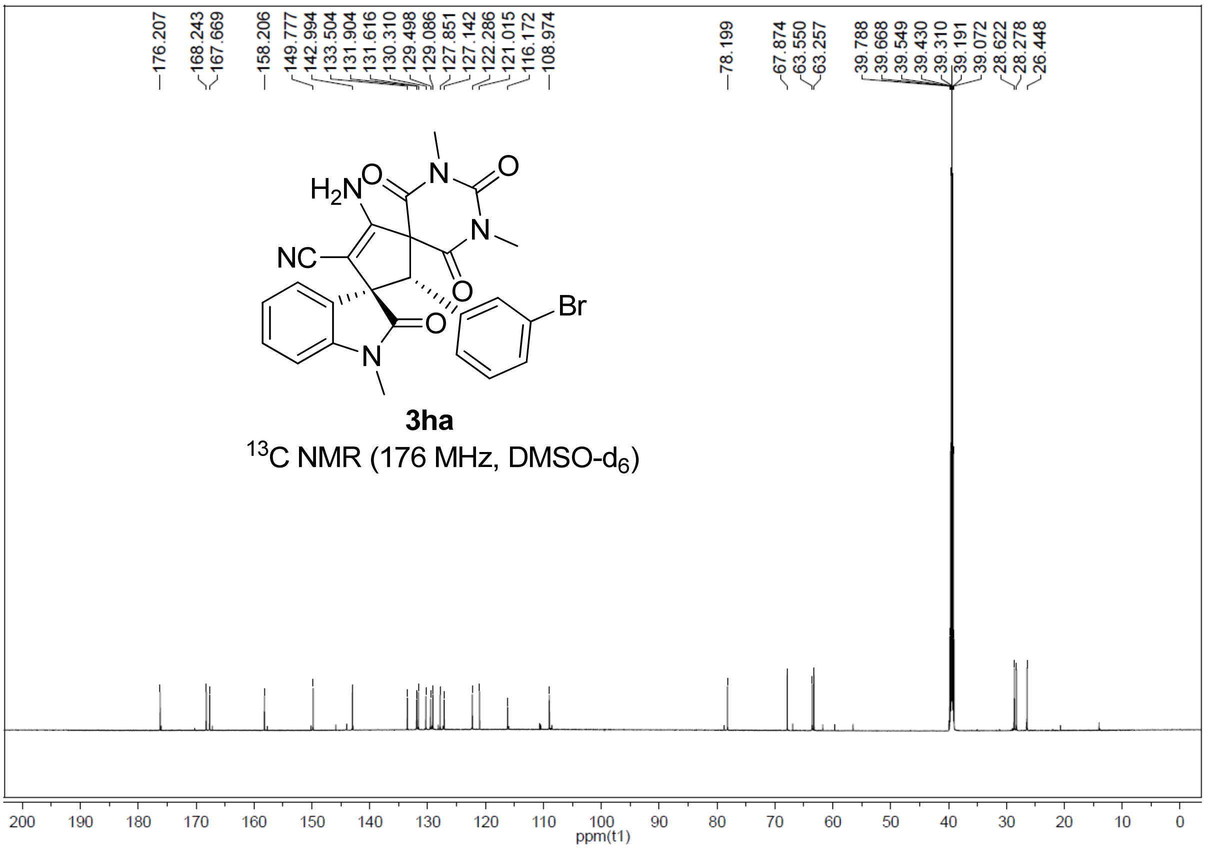

3ha

^{13}C NMR (176 MHz, DMSO-d$_6$)

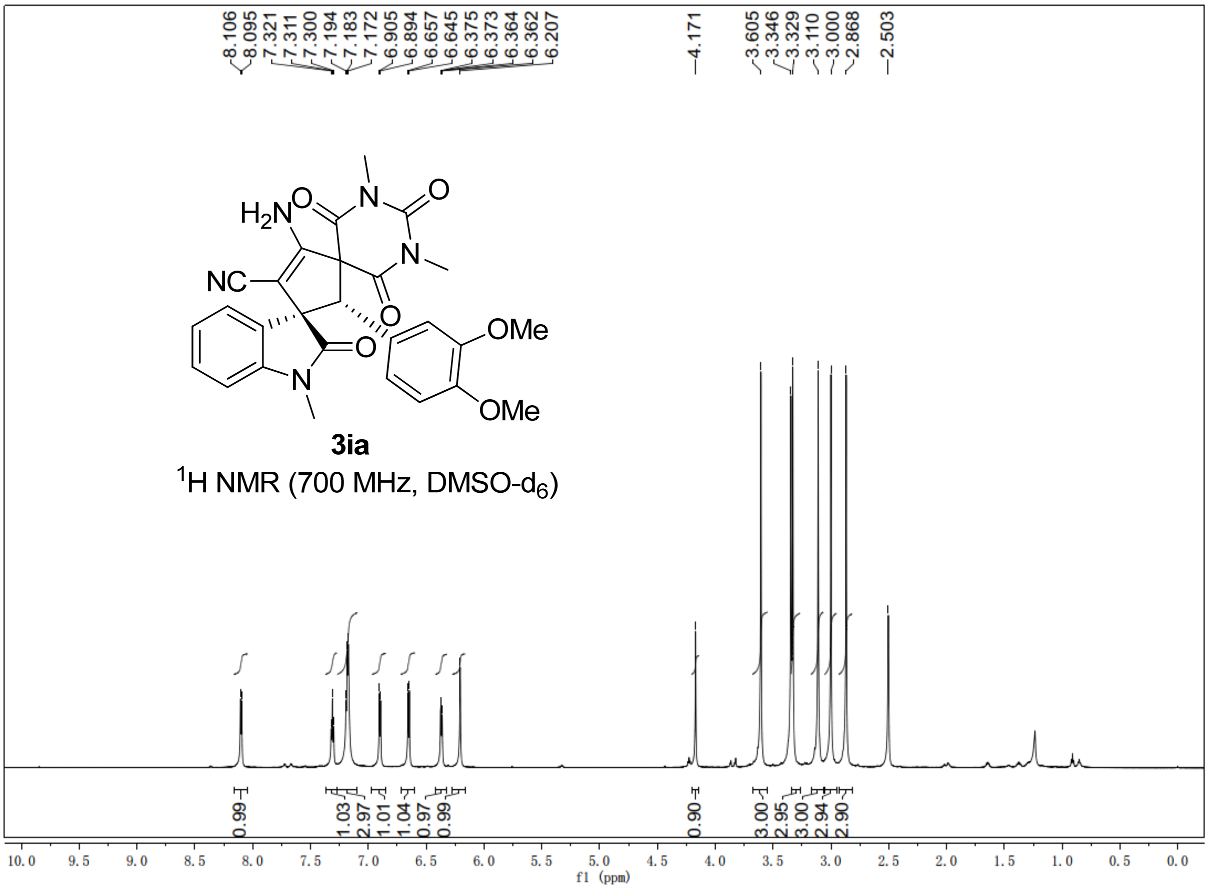

3ia

^{1}H NMR (700 MHz, DMSO-d$_6$)

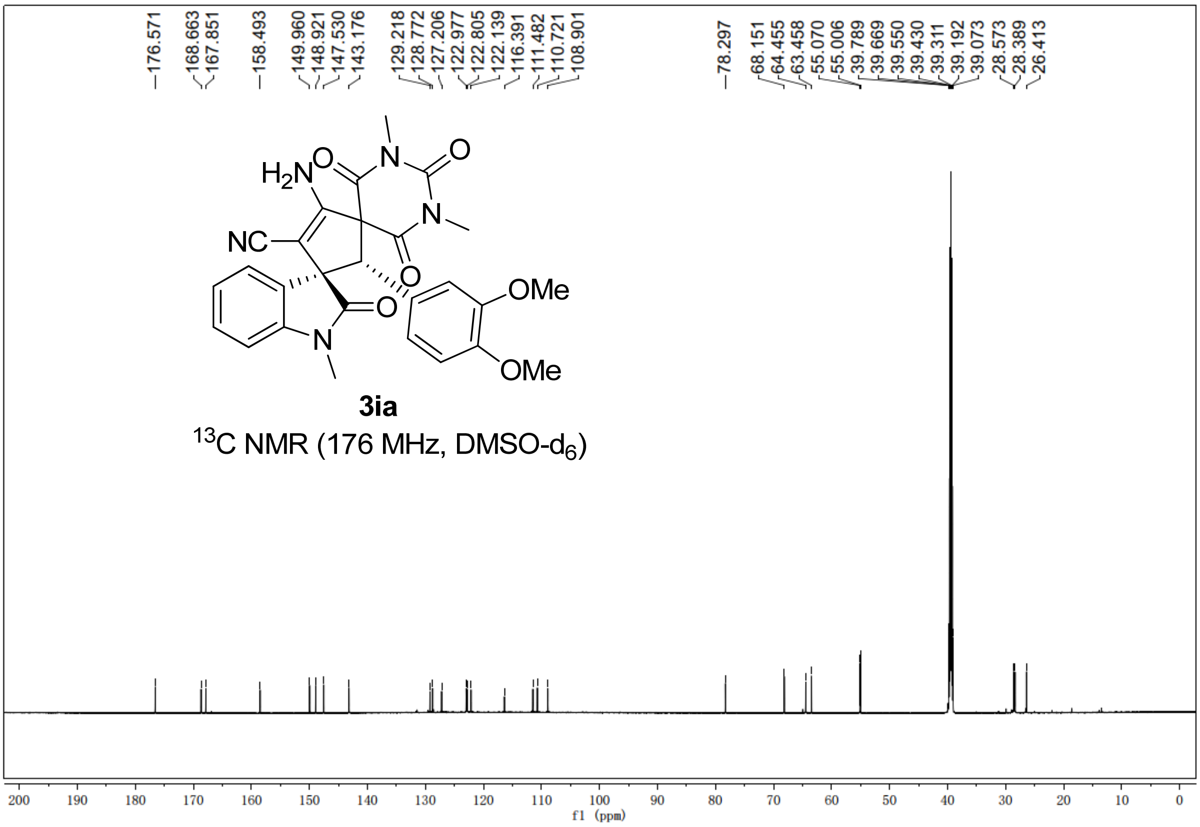
176.571
168.663
167.851
158.493
149.960
148.921
147.530
143.176
129.218
128.772
127.206
122.977
122.805
122.139
116.391
111.482
110.721
108.901
78.297
68.151
64.455
63.458
55.070
55.006
39.789
39.669
39.550
39.430
39.311
39.192
39.073
28.573
28.389
26.413
H2N
NC
O
N
OMe
3ia
13C NMR (176 MHz, DMSO-d6)
200 190 180 170 160 150 140 130 120 110 100 90 80 70 60 50 40 30 20 10 0
f1 (ppm)

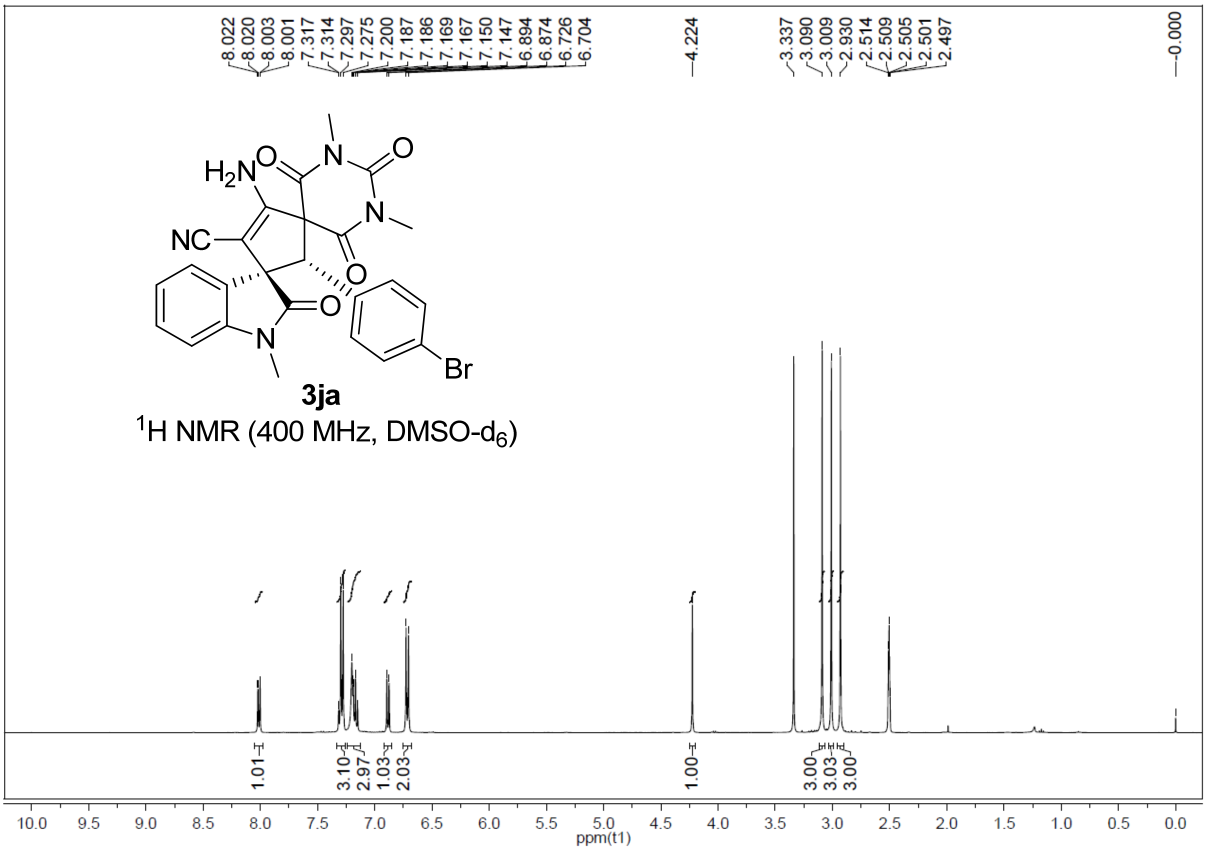
8.022
8.020
8.003
8.001
7.317
7.314
7.297
7.275
7.200
7.187
7.186
7.169
7.167
7.150
7.147
6.894
6.874
6.726
6.704
4.224
3.337
3.090
3.009
2.930
2.514
2.509
2.505
2.501
2.497
0.000
H2N
NC
O
N
Br
3ja
1H NMR (400 MHz, DMSO-d6)
1.01
3.10
2.97
1.03
2.03
1.00
3.00
3.03
3.00
10.0 9.5 9.0 8.5 8.0 7.5 7.0 6.5 6.0 5.5 5.0 4.5 4.0 3.5 3.0 2.5 2.0 1.5 1.0 0.5 0.0
ppm(t1)

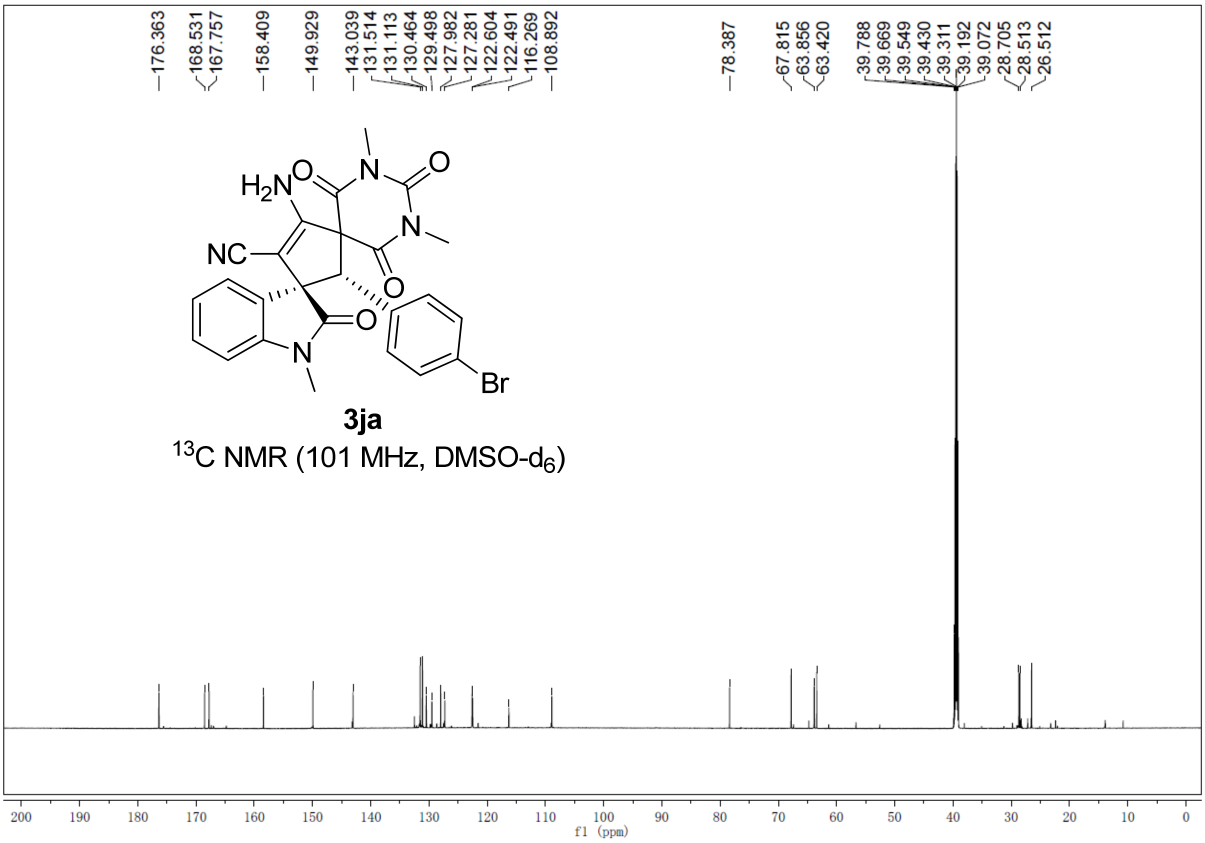
176.363
168.531
167.757
158.409
149.929
143.039
131.514
131.113
130.464
129.498
127.982
127.281
122.604
122.491
116.269
108.892
78.387
67.815
63.856
63.420
39.788
39.669
39.549
39.430
39.311
39.192
39.072
28.705
28.513
26.512
H2N
NC
Br
3ja
13C NMR (101 MHz, DMSO-d6)
f1 (ppm)

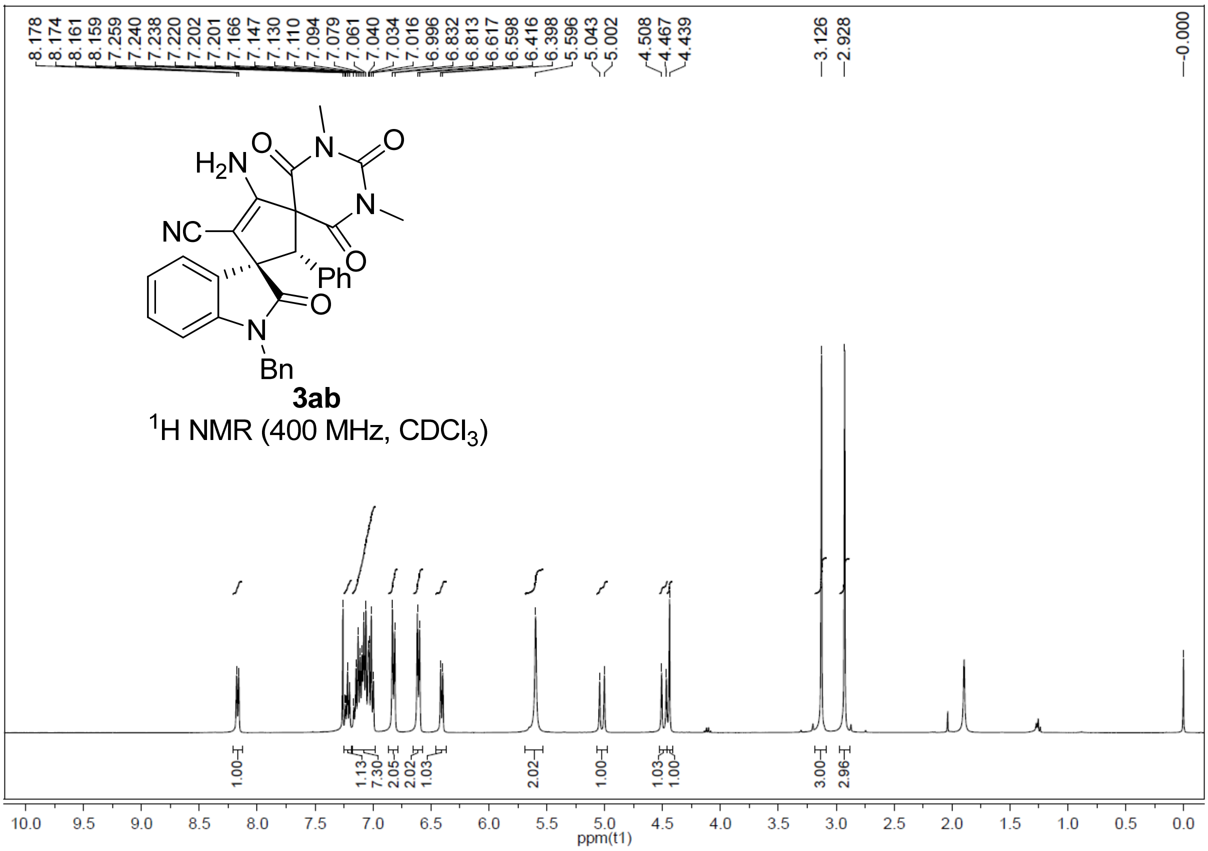
8.178
8.174
8.161
8.159
7.259
7.240
7.238
7.220
7.202
7.201
7.166
7.147
7.130
7.110
7.094
7.079
7.061
7.040
7.034
7.016
6.996
6.832
6.813
6.617
6.598
6.416
6.398
5.596
5.043
5.002
4.508
4.467
4.439
3.126
2.928
-0.000
H2N
NC
Ph
Bn
3ab
1H NMR (400 MHz, CDCl3)
1.00
1.13
7.30
2.05
2.02
1.03
2.02
1.00
1.03
1.00
3.00
2.96
ppm(t1)

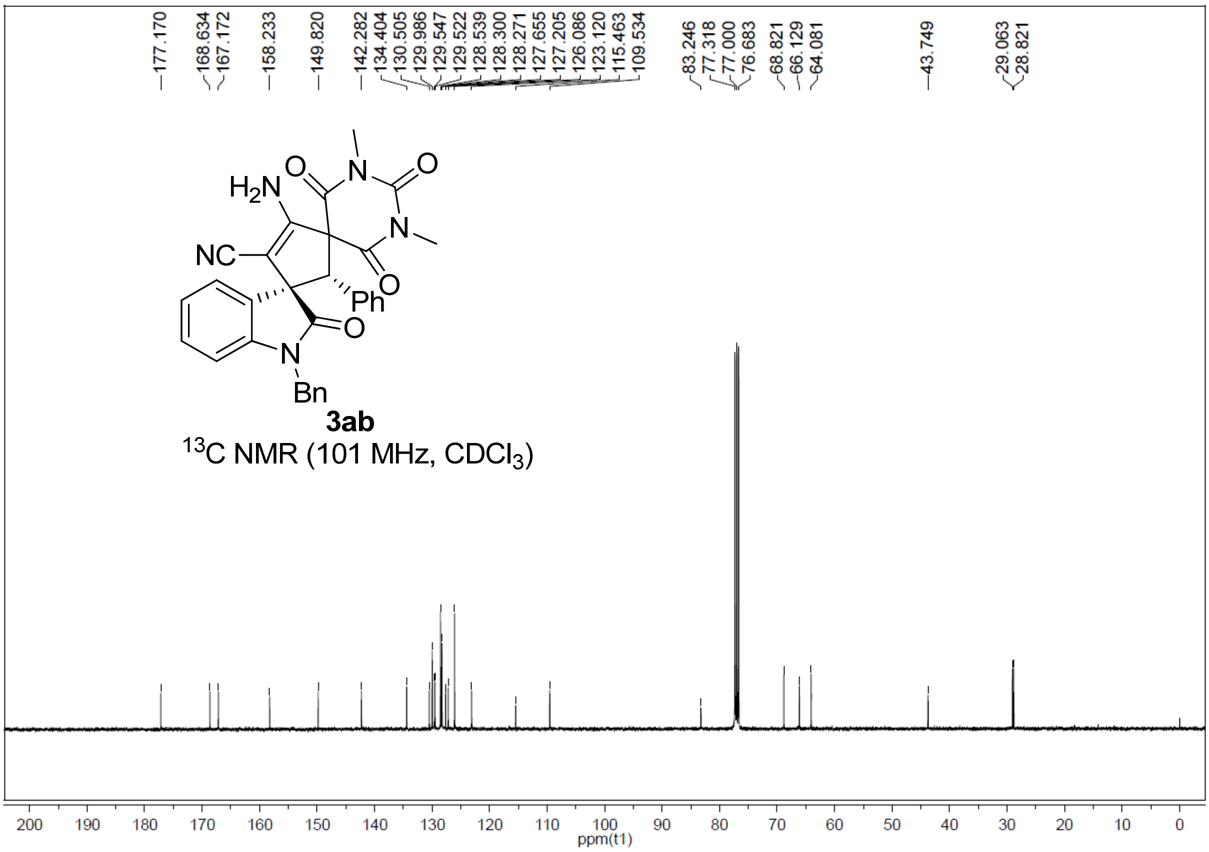

3ab
^{13}C NMR (101 MHz, $CDCl_3$)

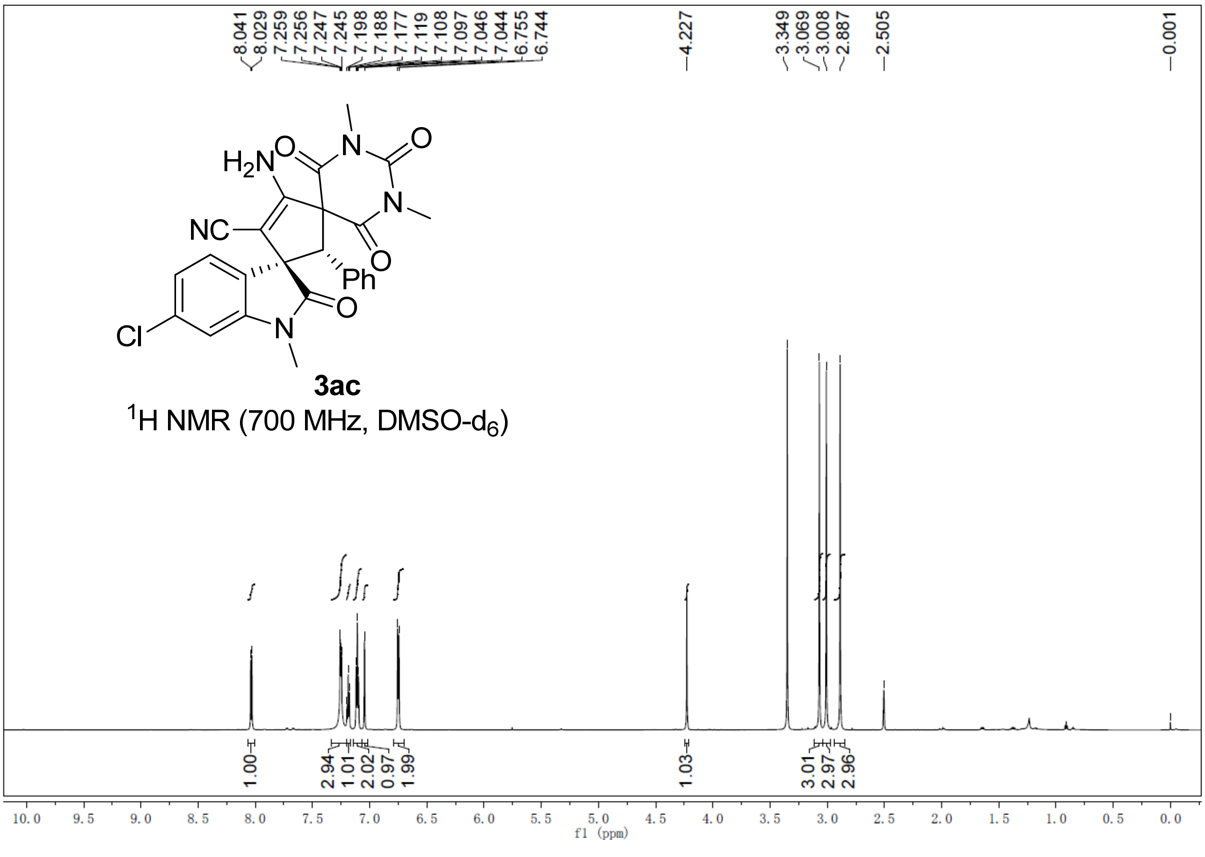

3ac
^{1}H NMR (700 MHz, DMSO-d_6)

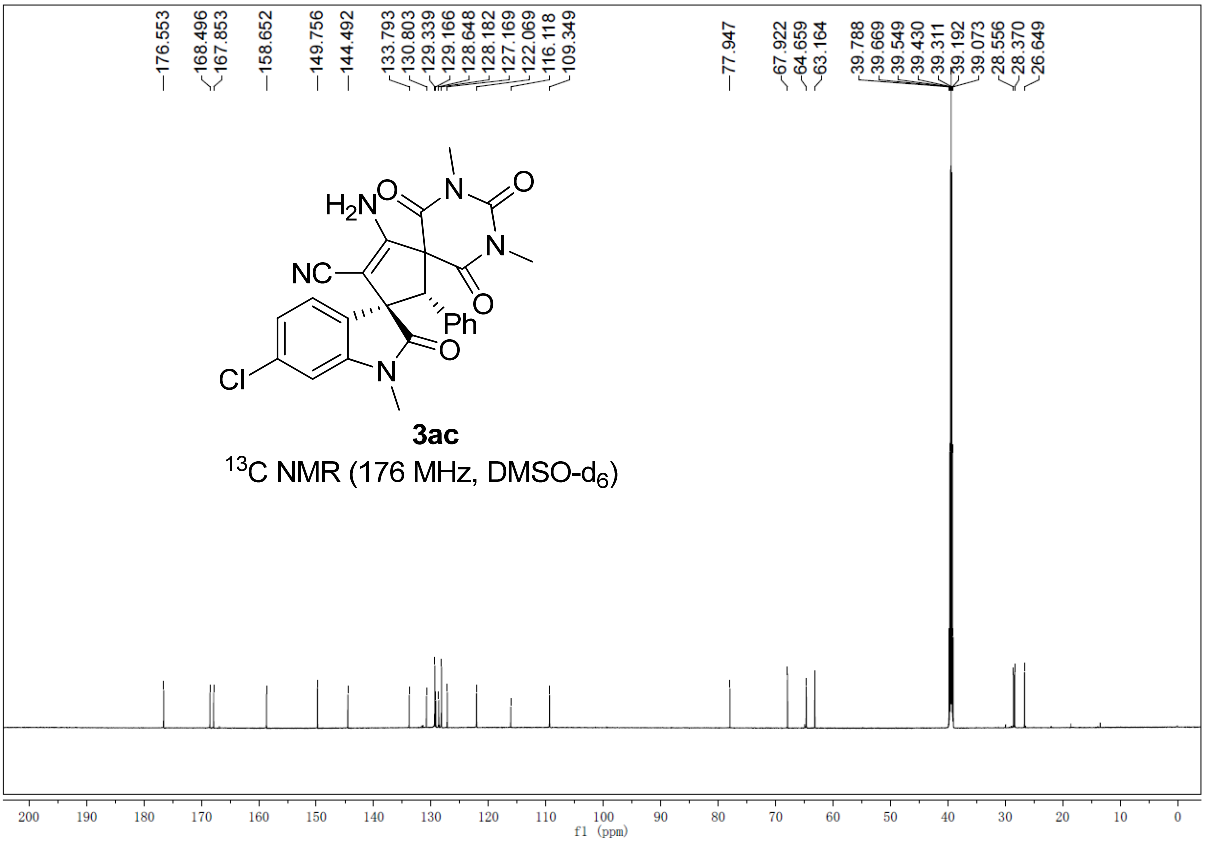
176.553
168.496
167.853
158.652
149.756
144.492
133.793
130.803
129.339
129.166
128.648
128.182
127.169
122.069
116.118
109.349
77.947
67.922
64.659
63.164
39.788
39.669
39.549
39.430
39.311
39.192
39.073
28.556
28.370
26.649
H2N
O
N
O
N
O
NC
Ph
O
Cl
N
3ac
13C NMR (176 MHz, DMSO-d6)
200 190 180 170 160 150 140 130 120 110 100 90 80 70 60 50 40 30 20 10 0
f1 (ppm)

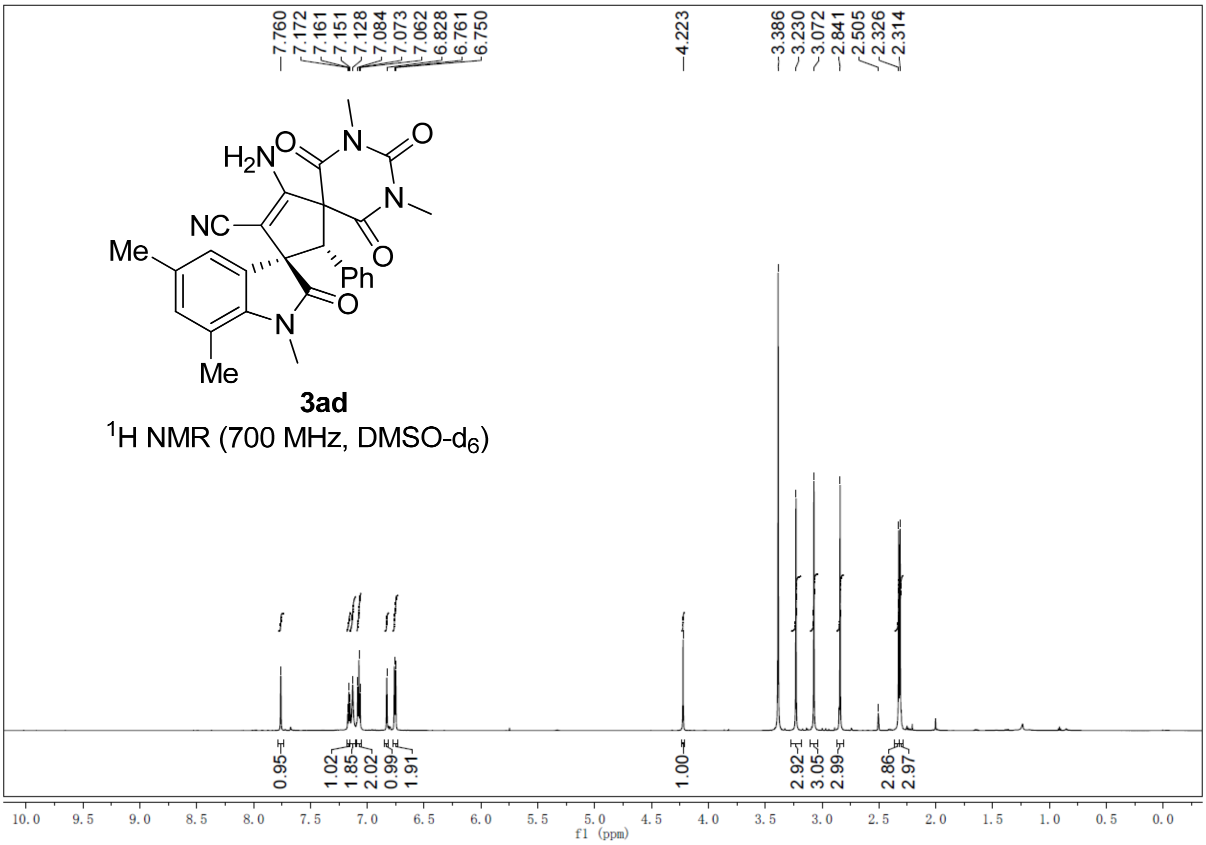
7.760
7.172
7.161
7.151
7.128
7.084
7.073
7.062
6.828
6.761
6.750
4.223
3.386
3.230
3.072
2.841
2.505
2.326
2.314
H2N
O
N
O
N
O
NC
Me
Ph
O
N
Me
3ad
1H NMR (700 MHz, DMSO-d6)
0.95
1.02
1.85
2.02
0.99
1.91
1.00
2.92
3.05
2.99
2.86
2.97
10.0 9.5 9.0 8.5 8.0 7.5 7.0 6.5 6.0 5.5 5.0 4.5 4.0 3.5 3.0 2.5 2.0 1.5 1.0 0.5 0.0
f1 (ppm)

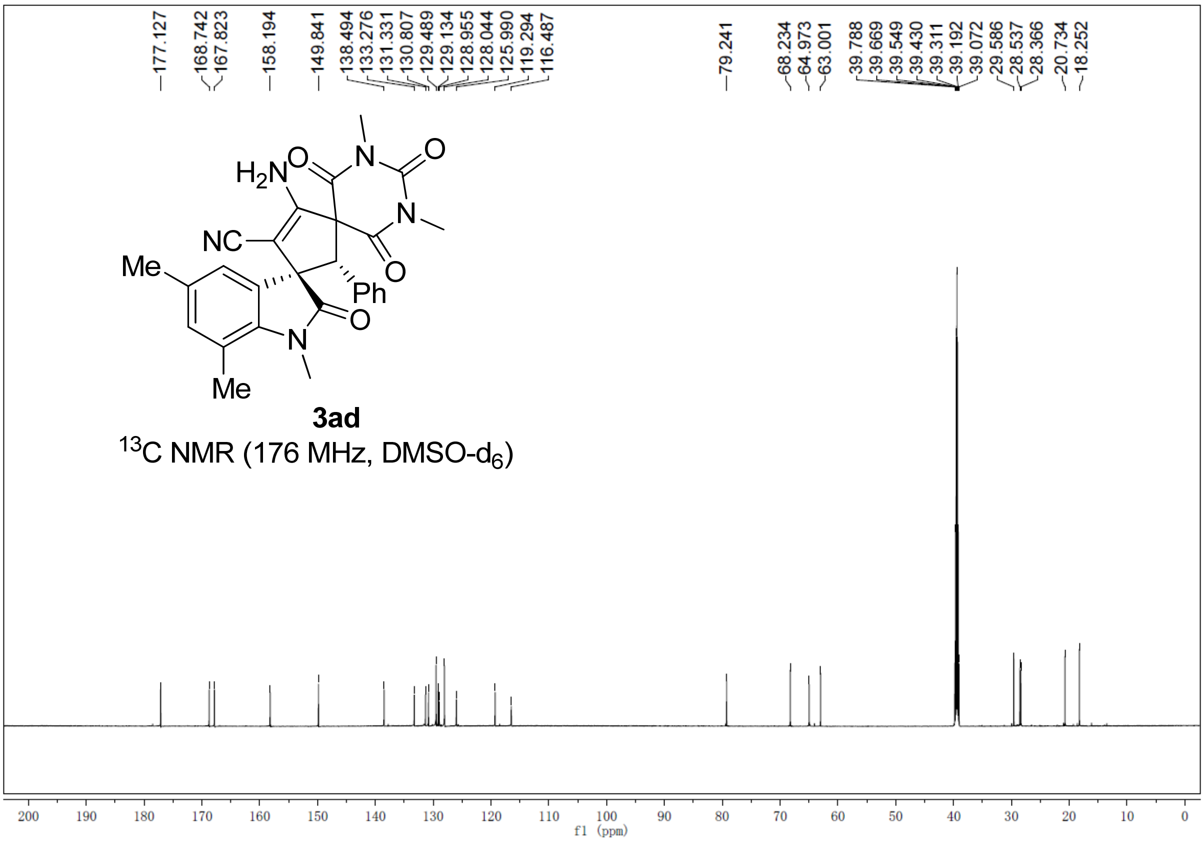
177.127
168.742
167.823
158.194
149.841
138.494
133.276
131.331
130.807
129.489
129.134
128.955
128.044
125.990
119.294
116.487
79.241
68.234
64.973
63.001
39.788
39.669
39.549
39.430
39.311
39.192
39.072
29.586
28.537
28.366
20.734
18.252
3ad
^{13}C NMR (176 MHz, DMSO-d_6)
f1 (ppm)

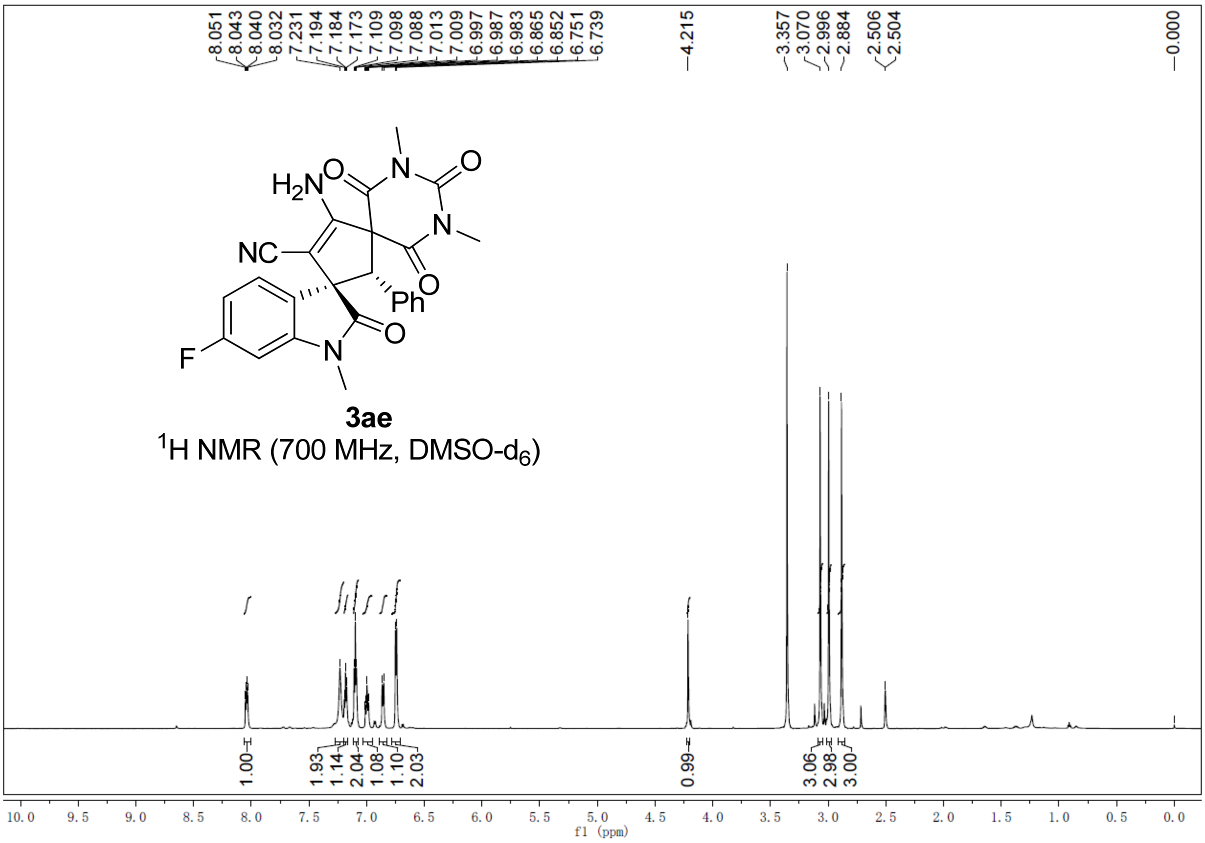
8.051
8.043
8.040
8.032
7.231
7.194
7.184
7.173
7.109
7.098
7.088
7.013
7.009
6.997
6.987
6.983
6.865
6.852
6.751
6.739
4.215
3.357
3.070
2.996
2.884
2.506
2.504
0.000
3ae
^{1}H NMR (700 MHz, DMSO-d_6)
1.00
1.93
1.14
2.04
1.08
1.10
2.03
0.99
3.06
2.98
3.00
f1 (ppm)

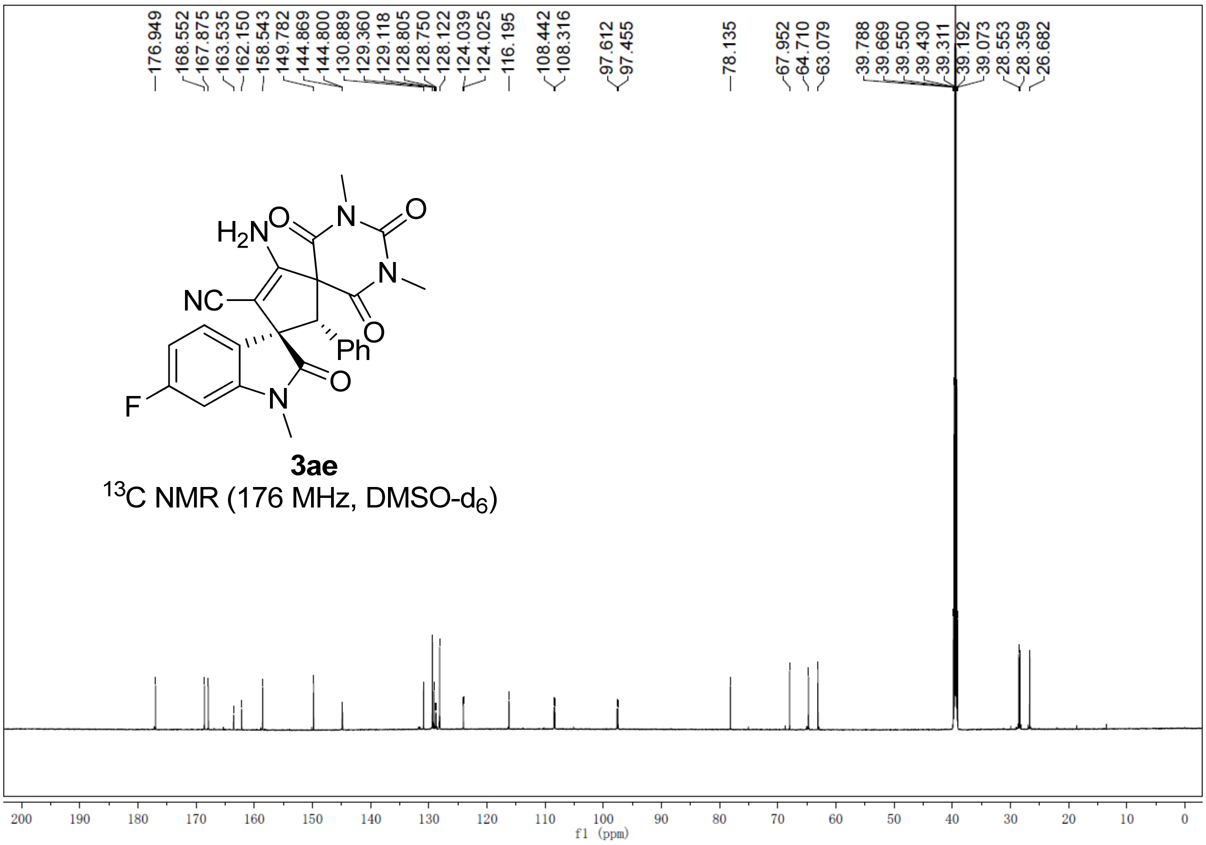
176.949
168.552
167.875
163.535
162.150
158.543
149.782
144.869
144.800
130.889
129.360
129.118
128.805
128.750
128.122
124.039
124.025
116.195
108.442
108.316
97.612
97.455
78.135
67.952
64.710
63.079
39.788
39.669
39.550
39.430
39.311
39.192
39.073
28.553
28.359
26.682
H2N
O
N
O
N
NC
O
Ph
F
N
O
3ae
13C NMR (176 MHz, DMSO-d6)
200 190 180 170 160 150 140 130 120 110 100 90 80 70 60 50 40 30 20 10 0
f1 (ppm)

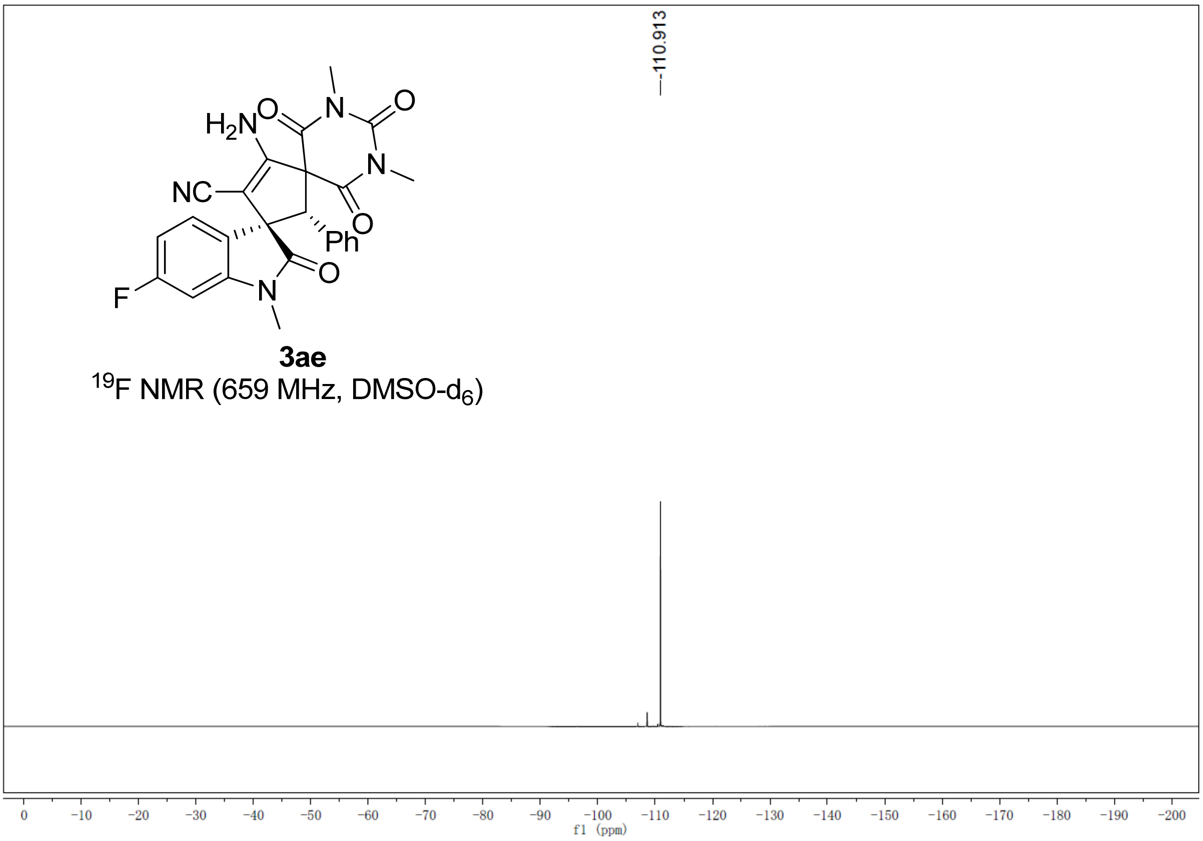
-110.913
H2N
O
N
O
N
NC
O
Ph
F
N
O
3ae
19F NMR (659 MHz, DMSO-d6)
0 -10 -20 -30 -40 -50 -60 -70 -80 -90 -100 -110 -120 -130 -140 -150 -160 -170 -180 -190 -200
f1 (ppm)

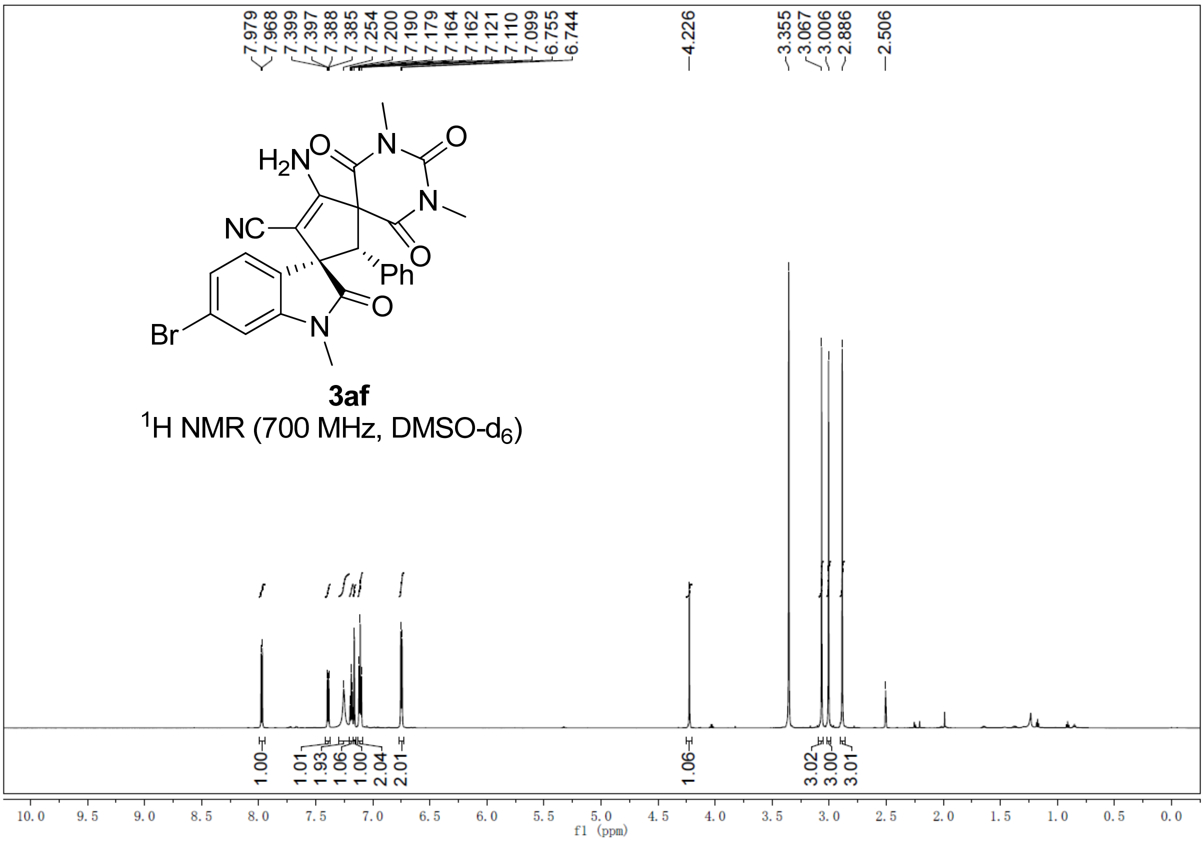

3af
^{1}H NMR (700 MHz, DMSO-d$_6$)
f1 (ppm)

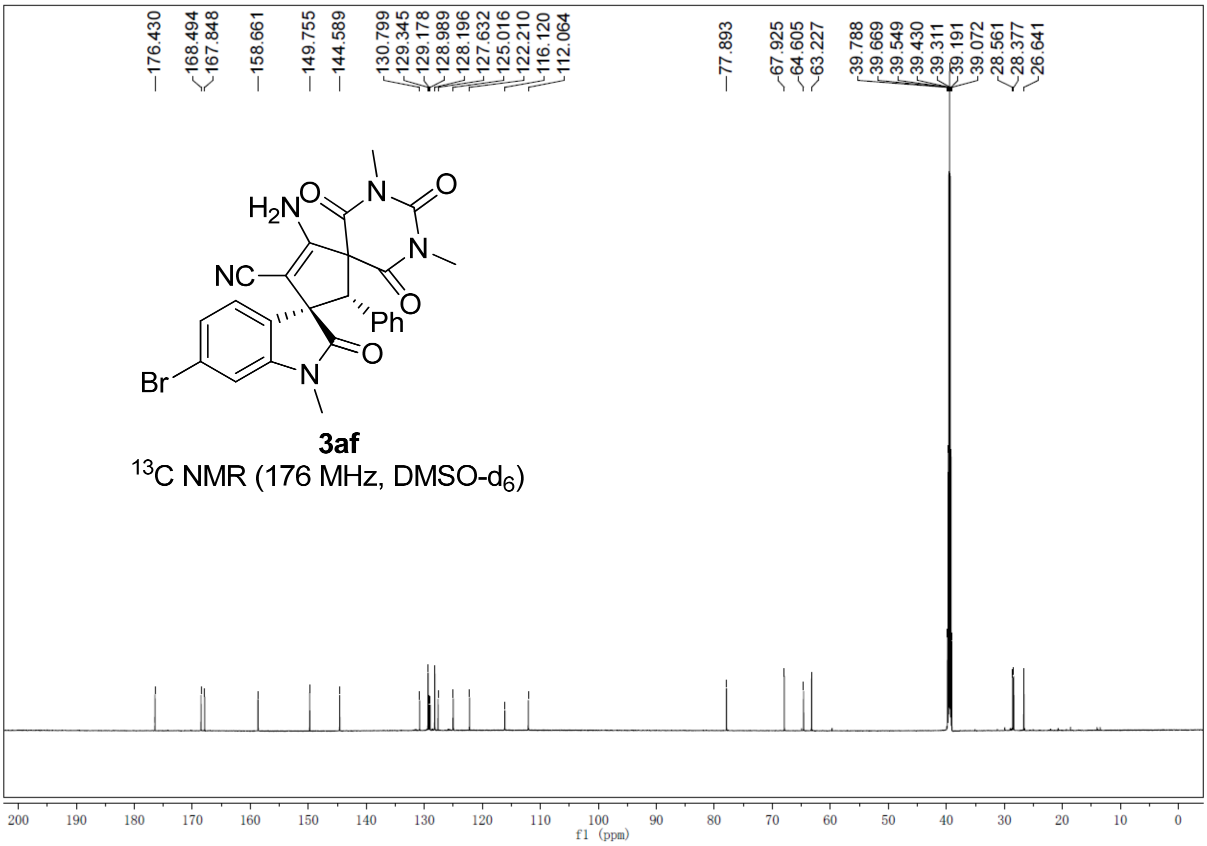

3af
^{13}C NMR (176 MHz, DMSO-d$_6$)
f1 (ppm)

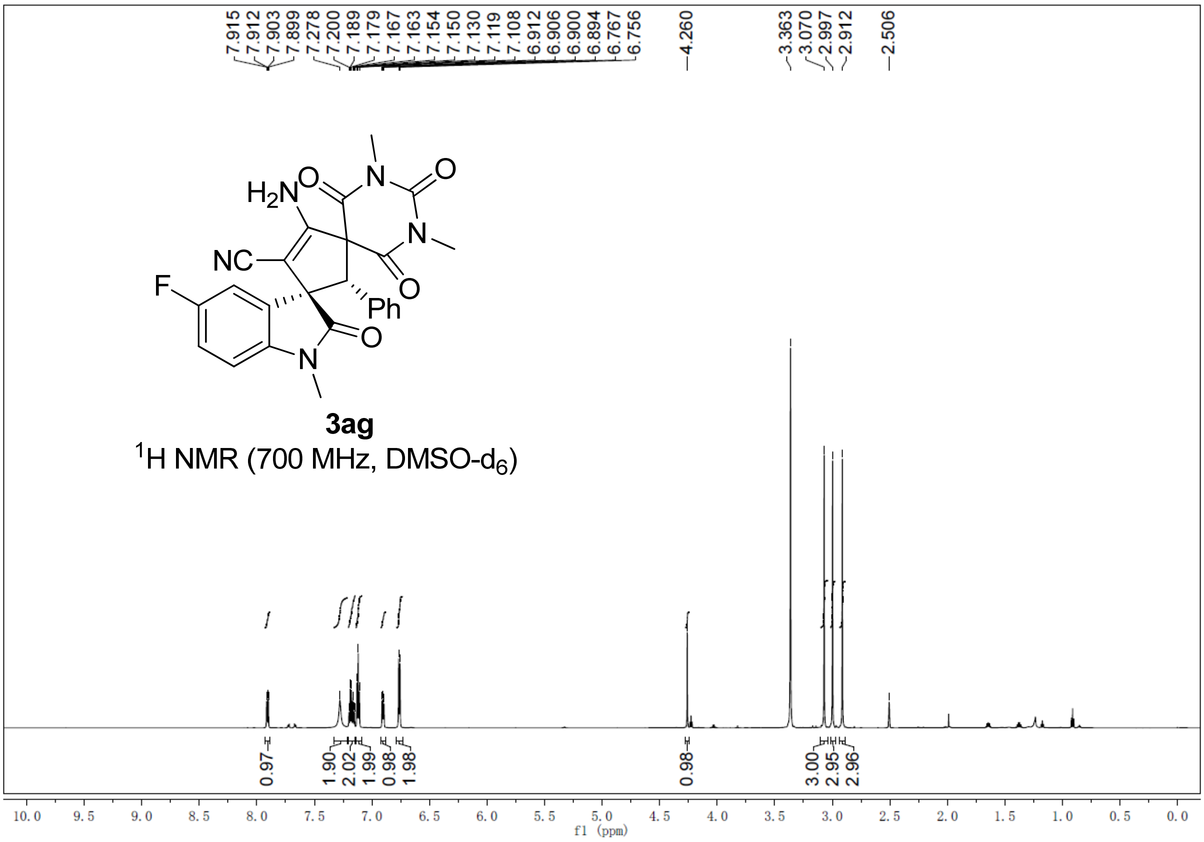
7.915
7.912
7.903
7.899
7.278
7.200
7.189
7.179
7.167
7.163
7.154
7.150
7.130
7.119
7.108
6.912
6.906
6.900
6.894
6.767
6.756
4.260
3.363
3.070
2.997
2.912
2.506
H2N
NC
F
Ph
3ag
1H NMR (700 MHz, DMSO-d6)
0.97
1.90
2.02
1.99
0.98
1.98
0.98
3.00
2.95
2.96
f1 (ppm)

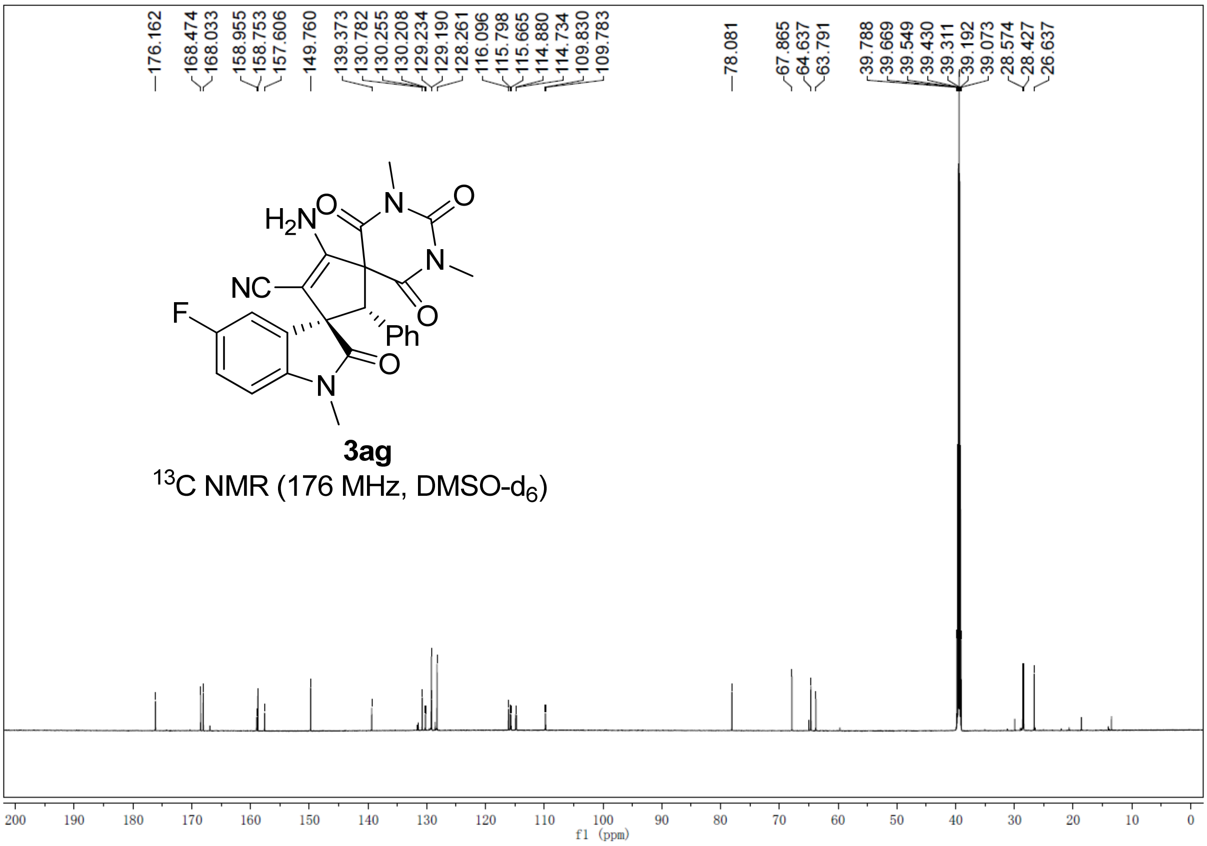
176.162
168.474
168.033
158.955
158.753
157.606
149.760
139.373
130.782
130.255
130.208
129.234
129.190
128.261
116.096
115.798
115.665
114.880
114.734
109.830
109.783
78.081
67.865
64.637
63.791
39.788
39.669
39.549
39.430
39.311
39.192
39.073
28.574
28.427
26.637
H2N
NC
F
Ph
3ag
13C NMR (176 MHz, DMSO-d6)
f1 (ppm)

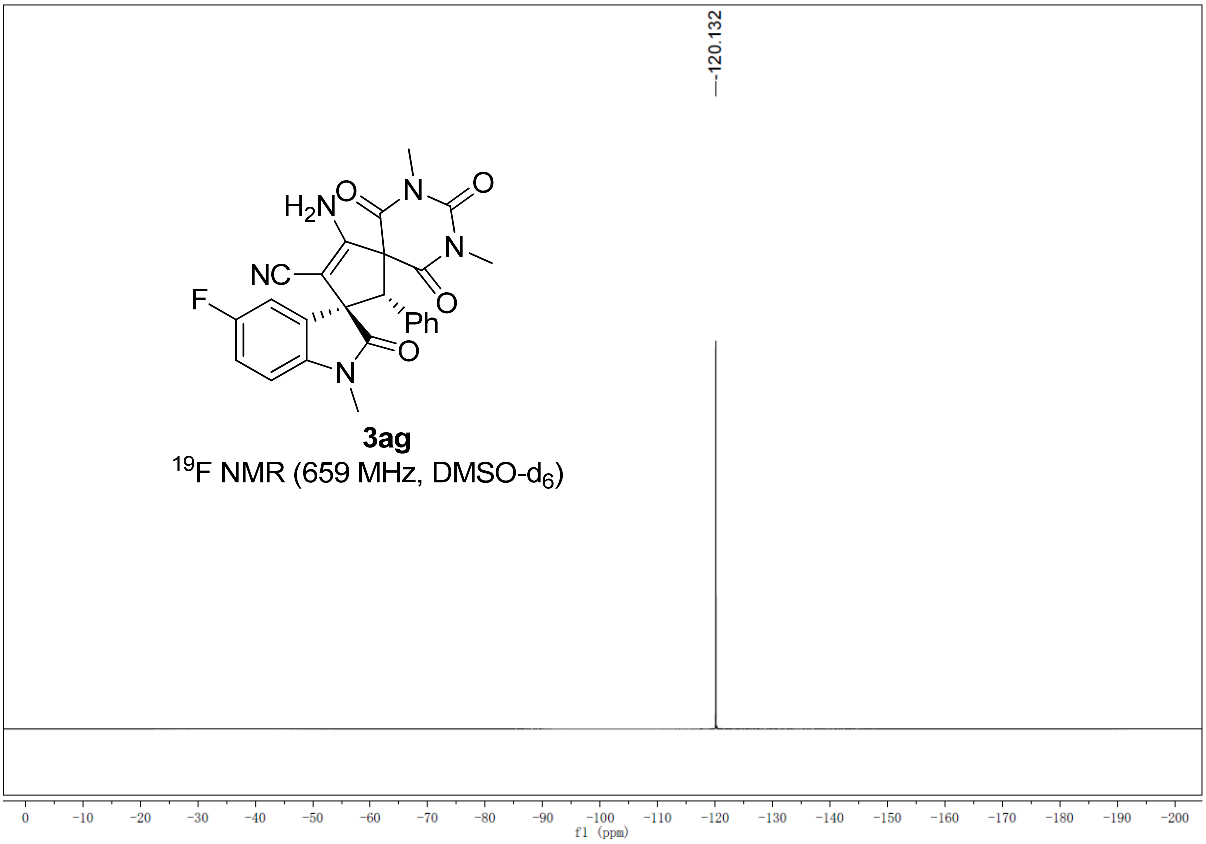
-120.132
3ag
^{19}F NMR (659 MHz, DMSO-d$_6$)
f1 (ppm)

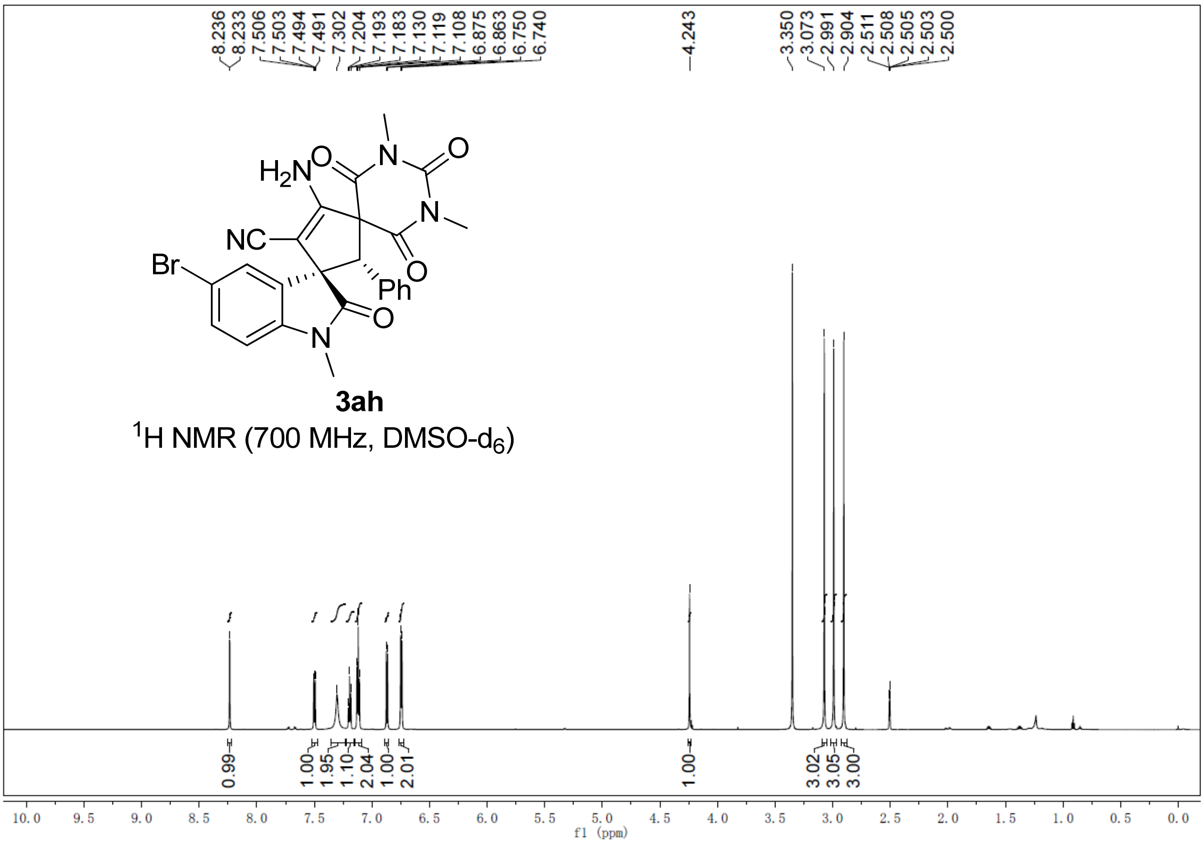
8.236
8.233
7.506
7.503
7.494
7.491
7.302
7.204
7.193
7.183
7.130
7.119
7.108
6.875
6.863
6.750
6.740
4.243
3.350
3.073
2.991
2.904
2.511
2.508
2.505
2.503
2.500
3ah
^{1}H NMR (700 MHz, DMSO-d$_6$)
0.99
1.00
1.95
1.10
2.04
1.00
2.01
1.00
3.02
3.05
3.00
f1 (ppm)

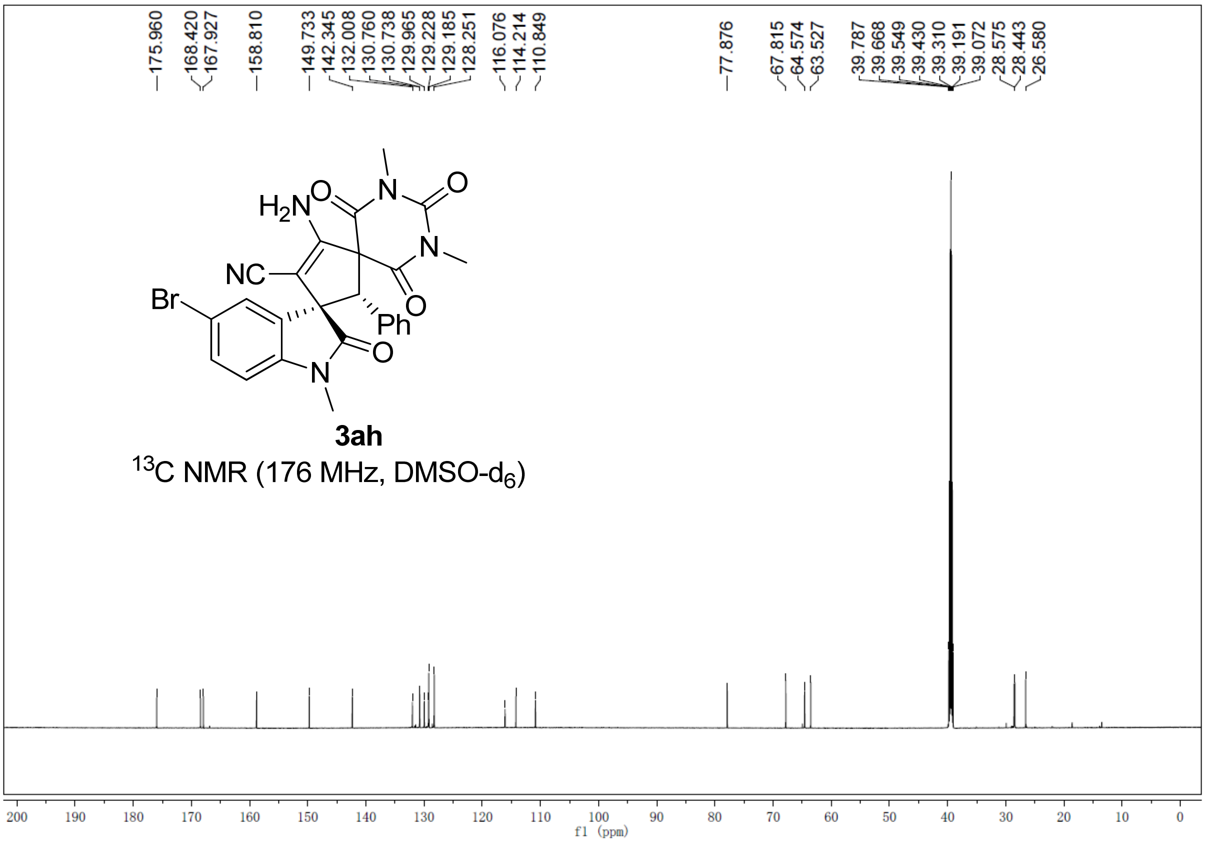
175.960
168.420
167.927
158.810
149.733
142.345
132.008
130.760
130.738
129.965
129.228
129.185
128.251
116.076
114.214
110.849
77.876
67.815
64.574
63.527
39.787
39.668
39.549
39.430
39.310
39.191
39.072
28.575
28.443
26.580
3ah
13C NMR (176 MHz, DMSO-d6)
f1 (ppm)

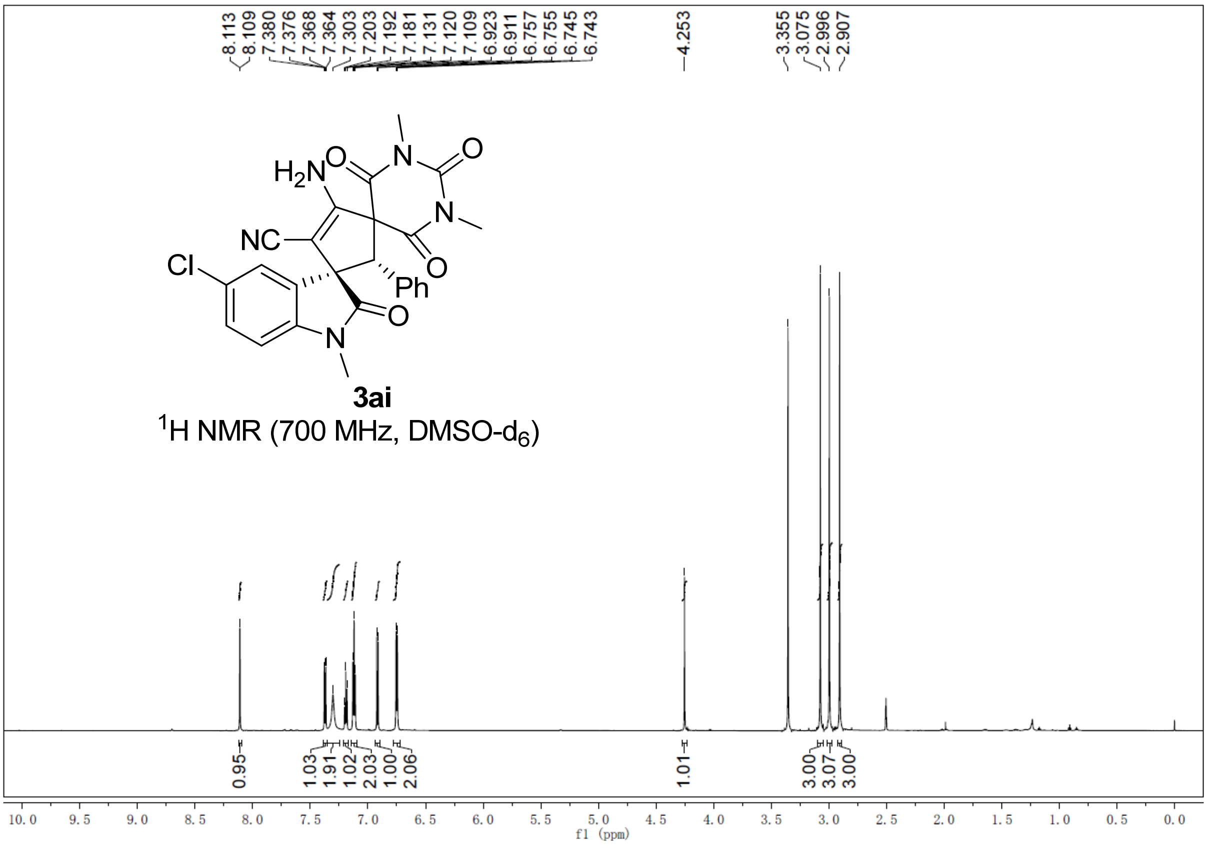
8.113
8.109
7.380
7.376
7.368
7.364
7.303
7.203
7.192
7.181
7.131
7.120
7.109
6.923
6.911
6.757
6.755
6.745
6.743
4.253
3.355
3.075
2.996
2.907
3ai
1H NMR (700 MHz, DMSO-d6)
0.95
1.03
1.91
1.02
2.03
1.00
2.06
1.01
3.00
3.07
3.00
f1 (ppm)

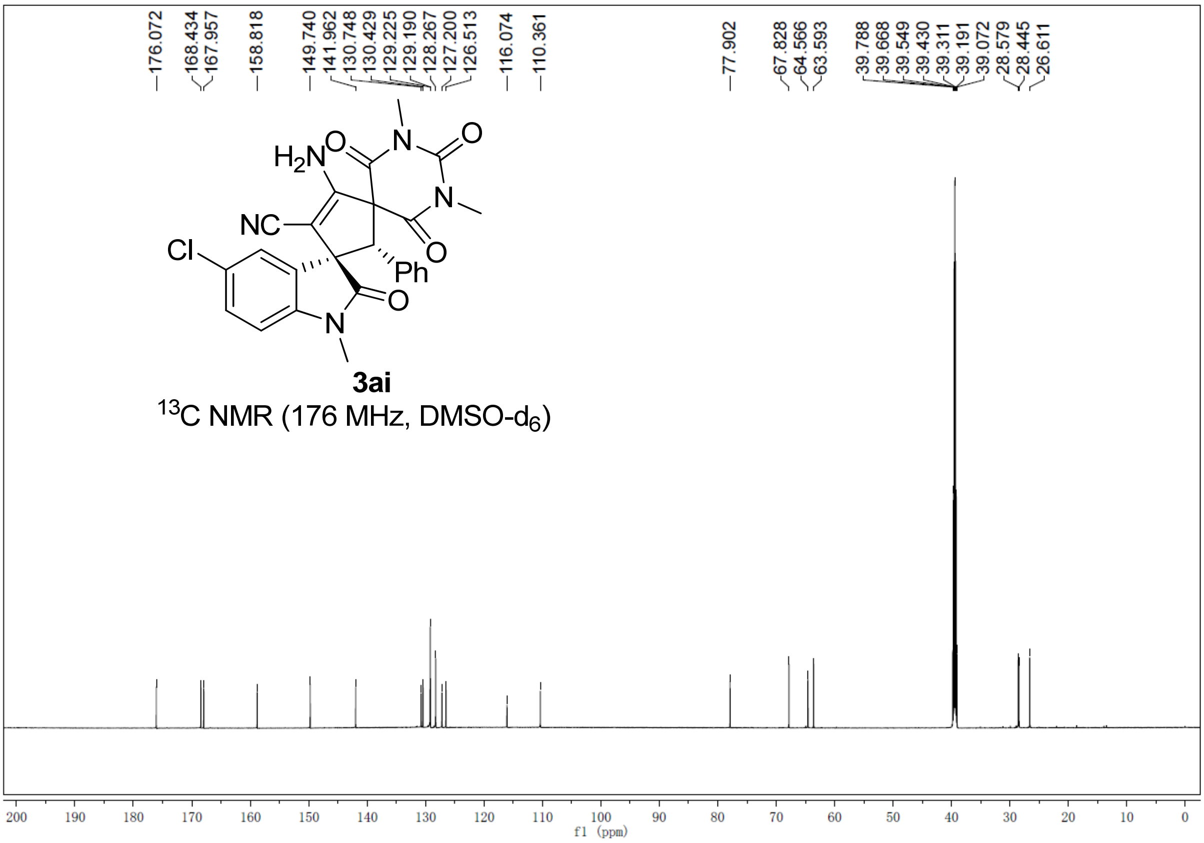

^{13}C NMR (176 MHz, DMSO-d_6)

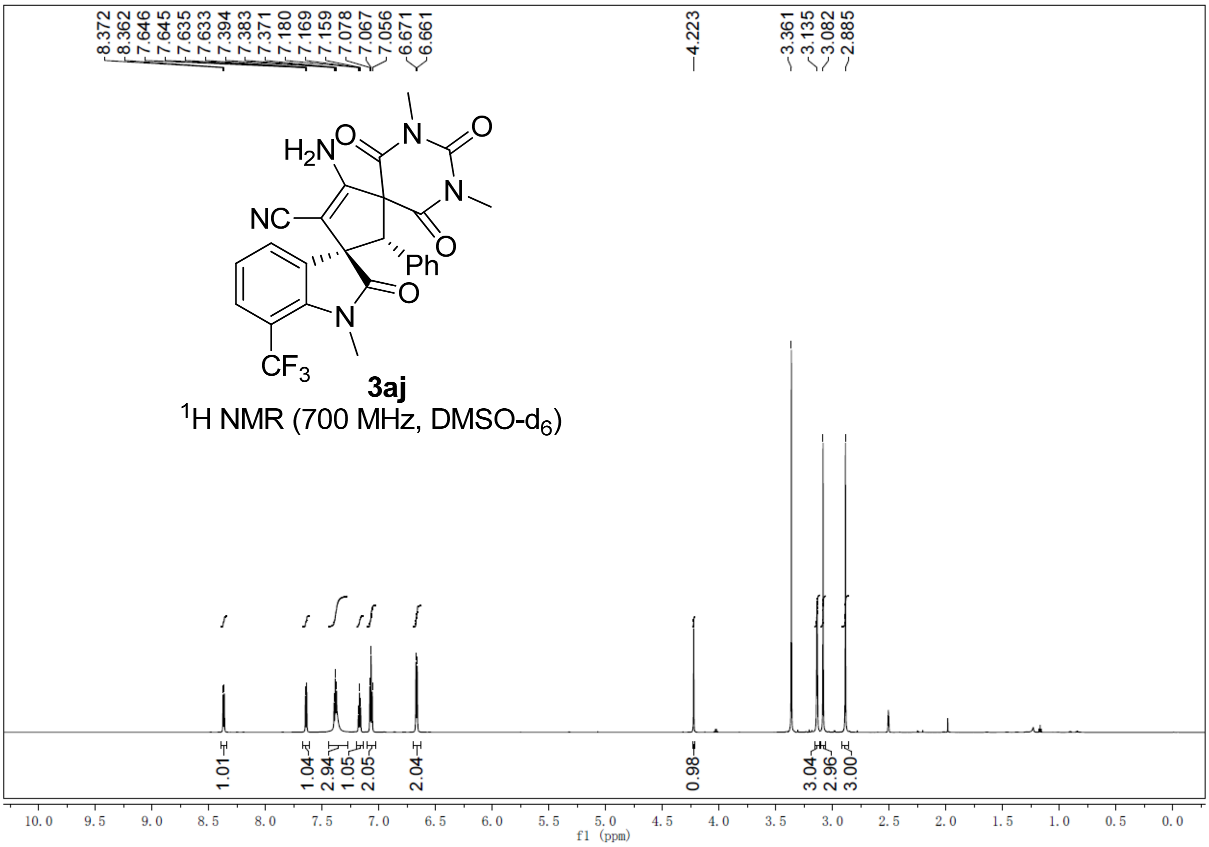

^{1}H NMR (700 MHz, DMSO-d_6)

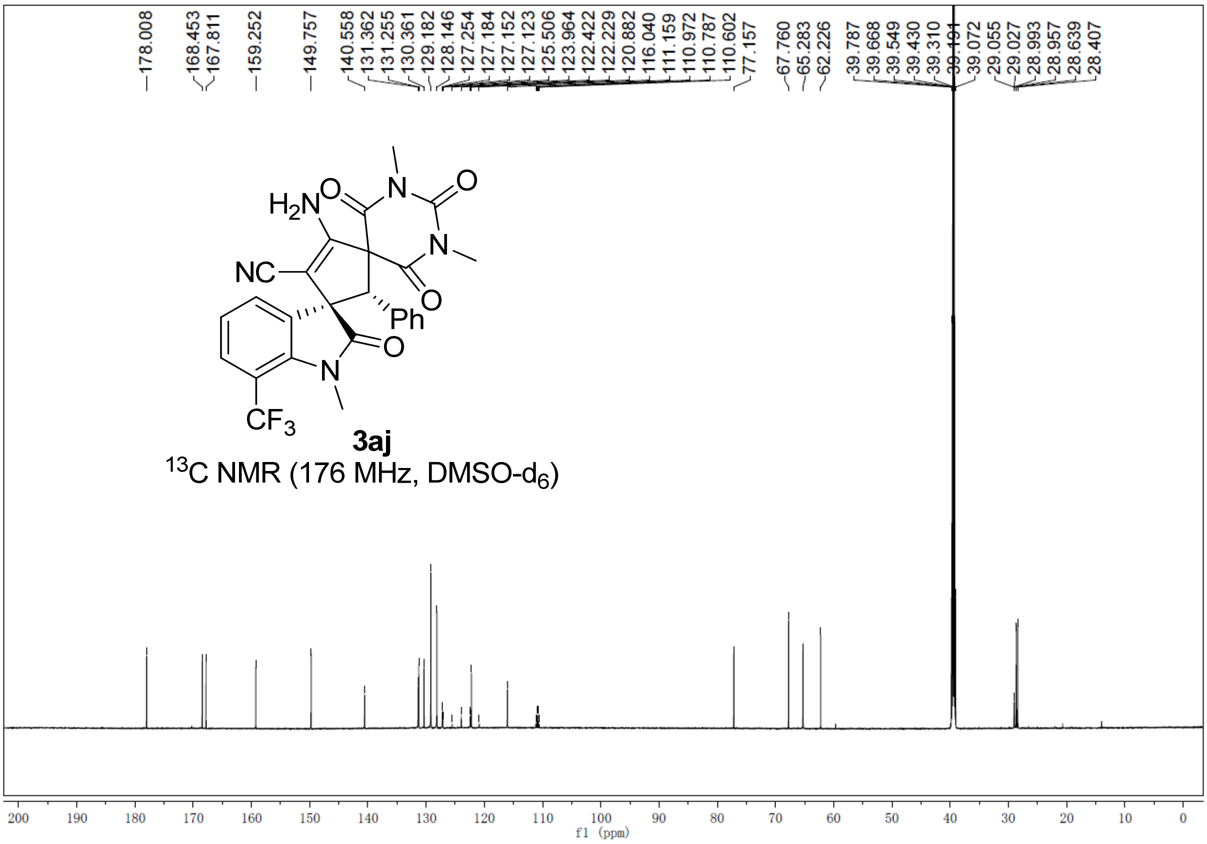
178.008
168.453
167.811
159.252
149.757
140.558
131.362
131.255
130.361
129.182
128.146
127.254
127.184
127.152
127.123
125.506
123.964
122.422
122.229
120.882
116.040
111.159
110.972
110.787
110.602
77.157
67.760
65.283
62.226
39.787
39.668
39.549
39.430
39.310
39.191
39.072
29.055
29.027
28.993
28.957
28.639
28.407
H2N
O
N
O
N
NC
O
Ph
O
N
CF3
3aj
13C NMR (176 MHz, DMSO-d6)
200 190 180 170 160 150 140 130 120 110 100 90 80 70 60 50 40 30 20 10 0
f1 (ppm)

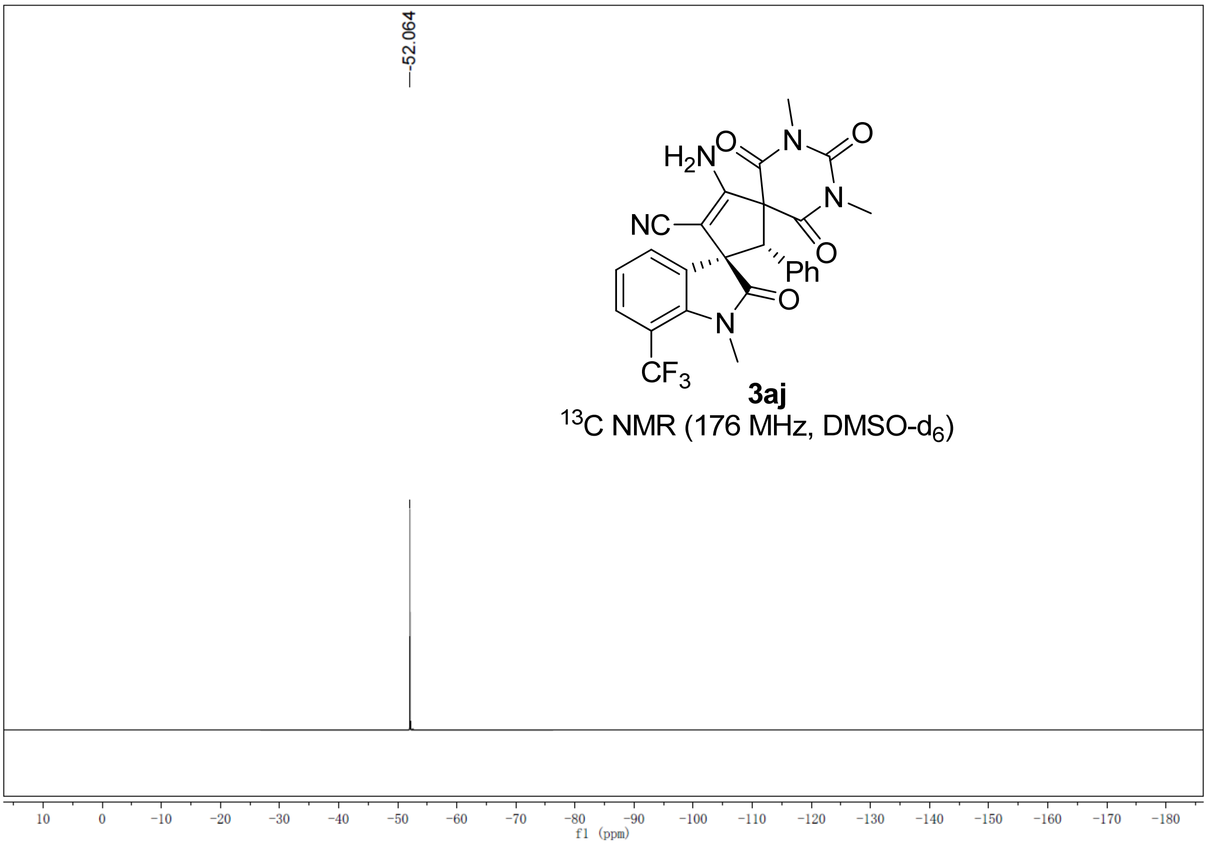
-52.064
H2N
O
N
O
N
NC
O
Ph
O
N
CF3
3aj
13C NMR (176 MHz, DMSO-d6)
10 0 -10 -20 -30 -40 -50 -60 -70 -80 -90 -100 -110 -120 -130 -140 -150 -160 -170 -180
f1 (ppm)

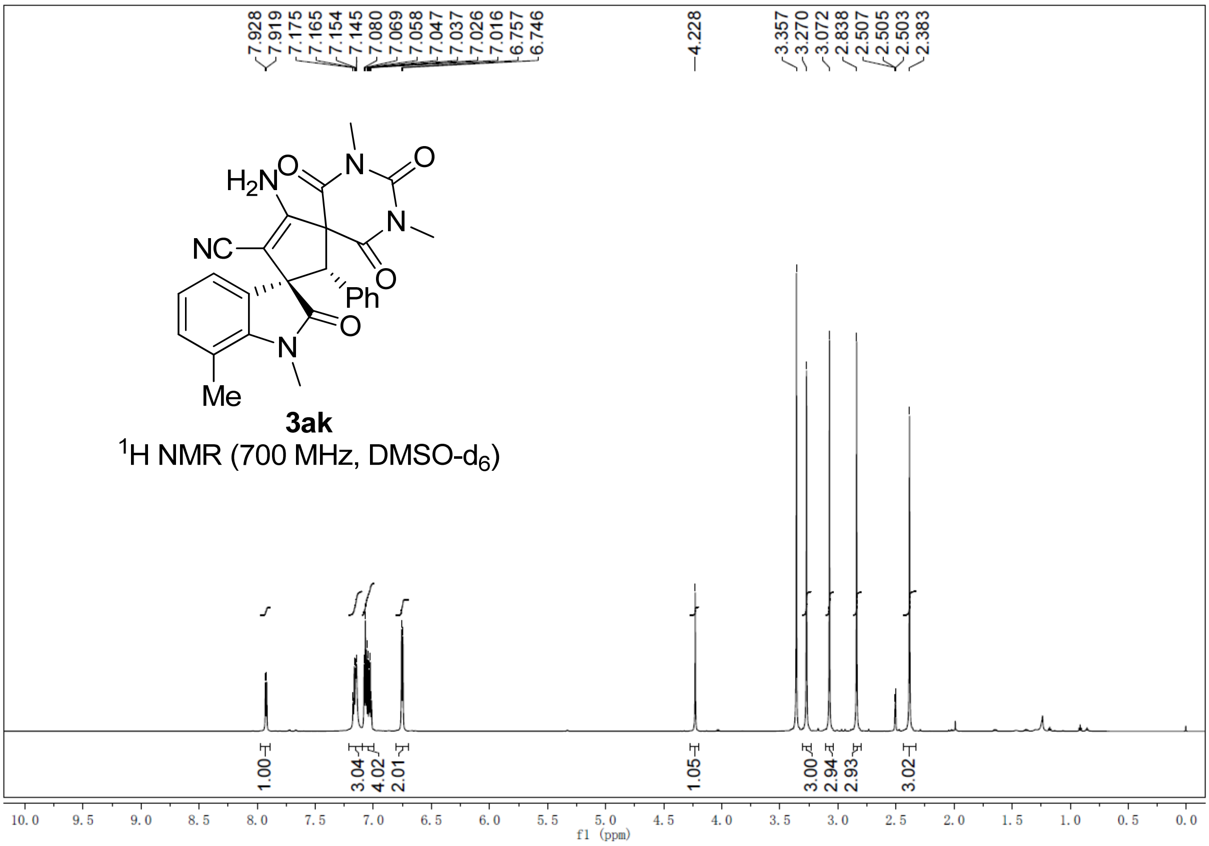
7.928
7.919
7.175
7.165
7.154
7.145
7.080
7.069
7.058
7.047
7.037
7.026
7.016
6.757
6.746
4.228
3.357
3.270
3.072
2.838
2.507
2.505
2.503
2.383
H2N
O
N
O
NC
N
O
Ph
O
N
Me
3ak
1H NMR (700 MHz, DMSO-d6)
1.00
3.04
4.02
2.01
1.05
3.00
2.94
2.93
3.02
f1 (ppm)

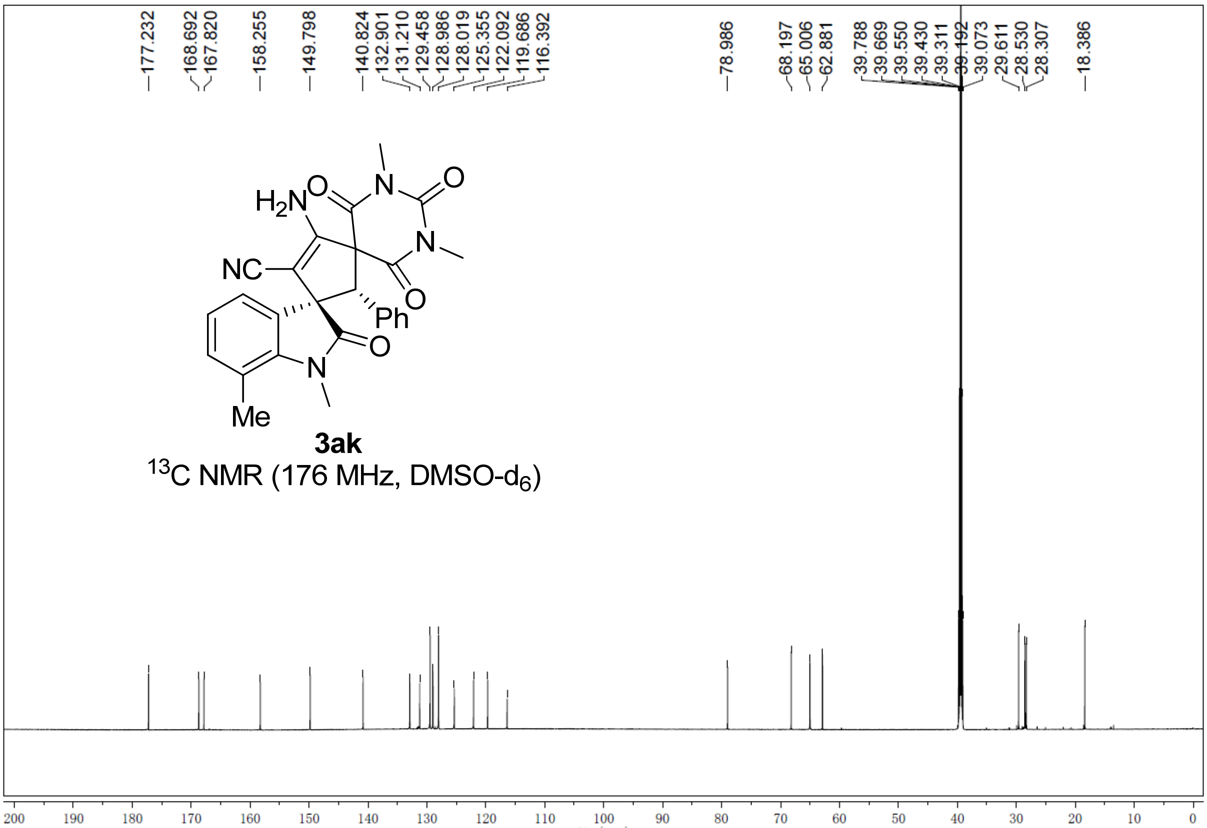
177.232
168.692
167.820
158.255
149.798
140.824
132.901
131.210
129.458
128.986
128.019
125.355
122.092
119.686
116.392
78.986
68.197
65.006
62.881
39.788
39.669
39.550
39.430
39.311
39.192
39.073
29.611
28.530
28.307
18.386
H2N
O
N
O
NC
N
O
Ph
O
N
Me
3ak
13C NMR (176 MHz, DMSO-d6)
f1 (ppm)

2. Copies of HPLC chromatograms

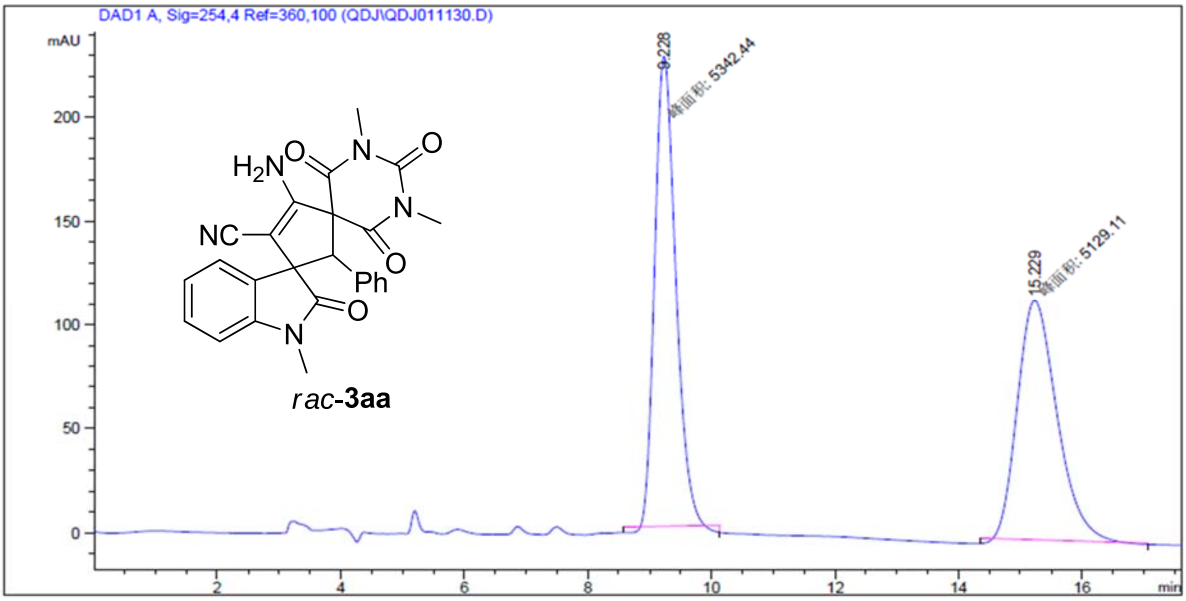

Peak #	RetTime [min]	Type	Width [min]	Area [mAU*s]	Height [mAU]	Area %
1	9.228	MM	0.3936	5342.43652	226.23160	51.0186
2	15.229	MM	0.7424	5129.11377	115.14812	48.9814

（峰面积：peak area）

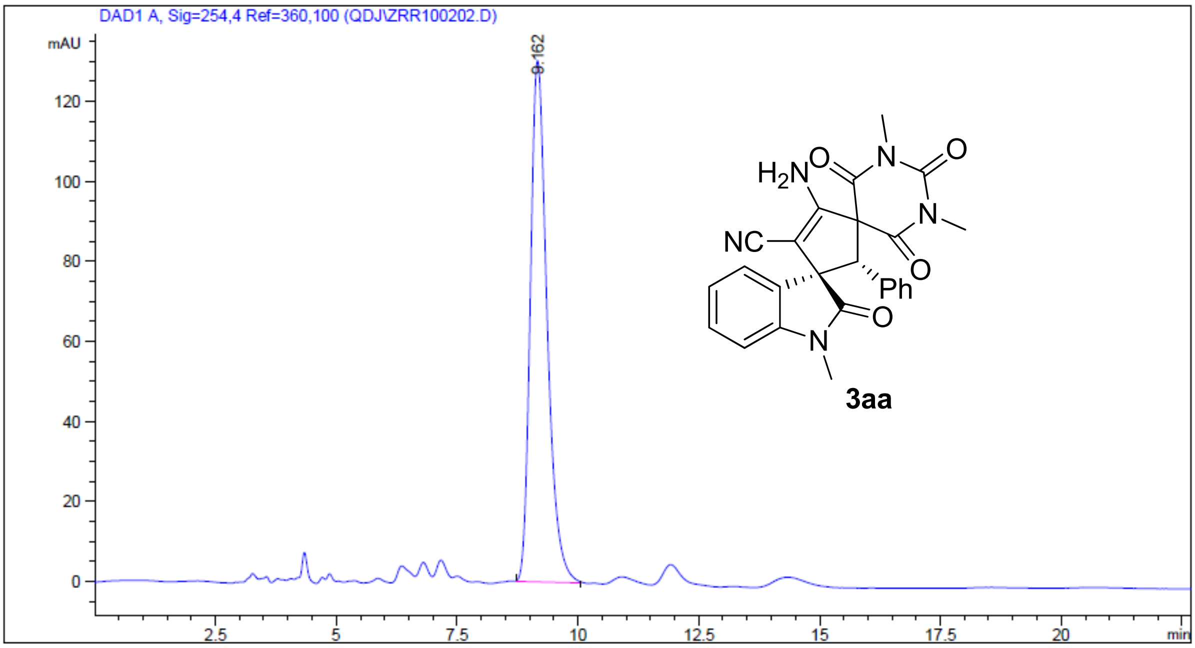

Peak #	RetTime [min]	Type	Width [min]	Area [mAU*s]	Height [mAU]	Area %
1	9.162	BB	0.3669	3138.23413	130.32306	100.0000

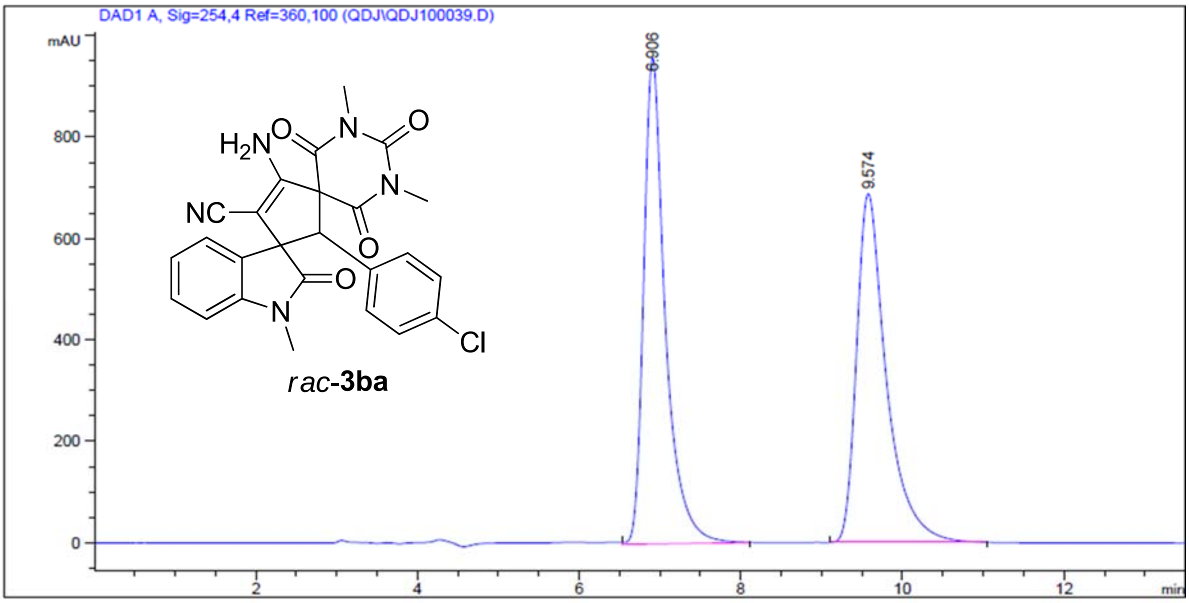

Peak #	RetTime [min]	Type	Width [min]	Area [mAU*s]	Height [mAU]	Area %
1	6.906	VB	0.2751	1.78500e4	959.07739	50.2701
2	9.574	BB	0.3793	1.76582e4	688.23169	49.7299

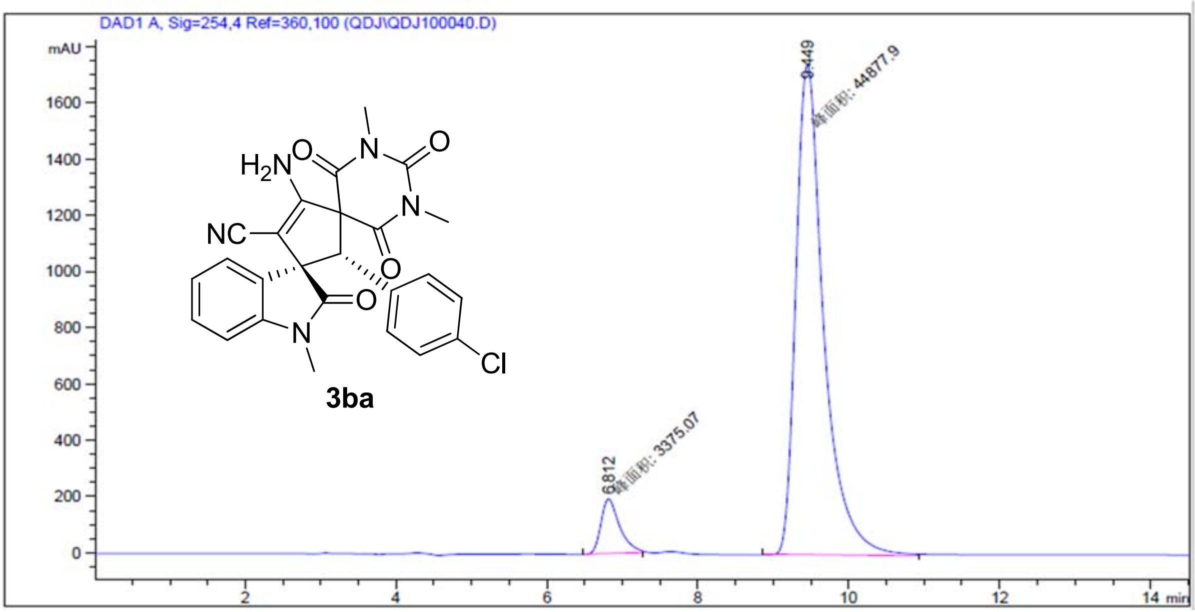

Peak #	RetTime [min]	Type	Width [min]	Area [mAU*s]	Height [mAU]	Area %
1	6.812	MM	0.2888	3375.06982	194.74199	6.9945
2	9.449	MM	0.4298	4.48779e4	1740.22803	93.0055

（峰面积：peak area）

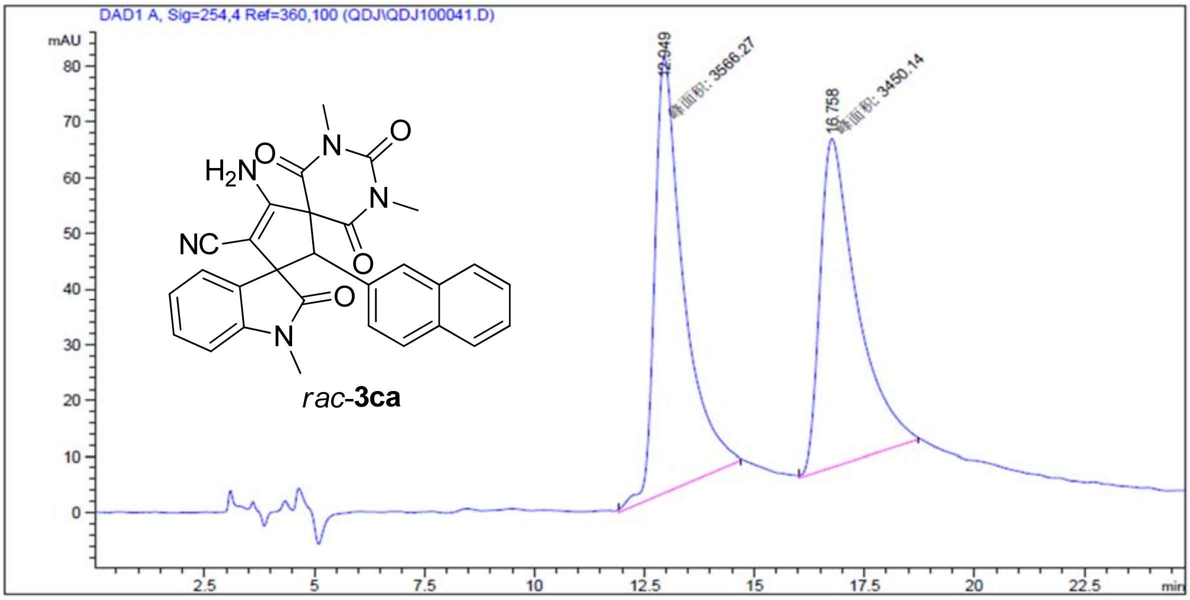

Peak #	RetTime [min]	Type	Width [min]	Area [mAU*s]	Height [mAU]	Area %
1	12.949	MM	0.7603	3566.27319	78.18192	50.8276
2	16.758	MM	0.9760	3450.13721	58.91587	49.1724

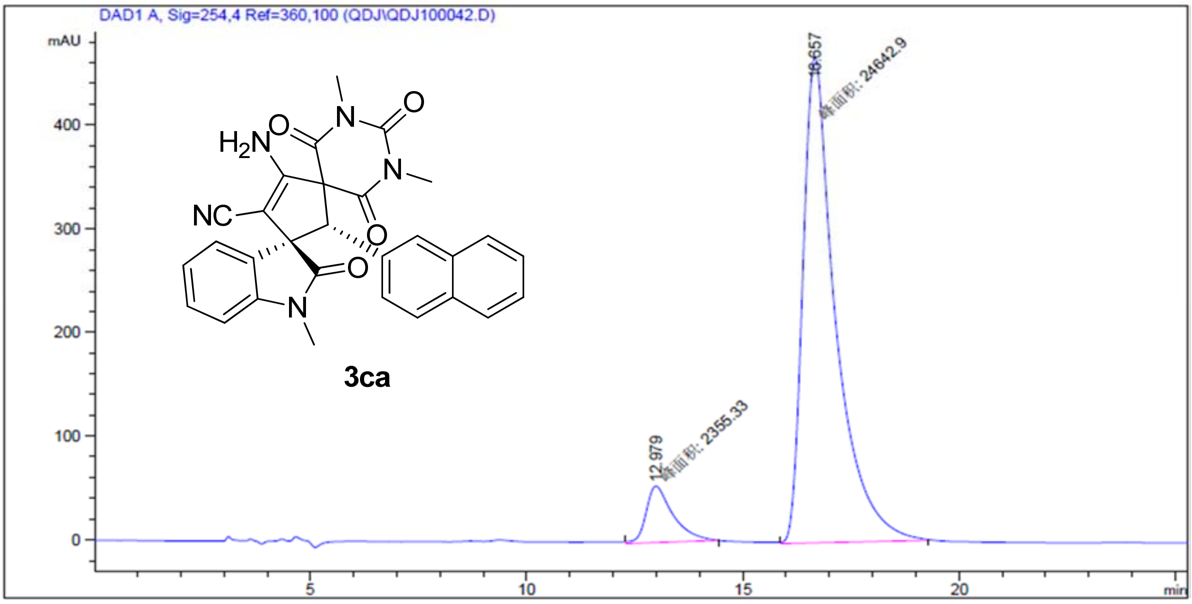

Peak #	RetTime [min]	Type	Width [min]	Area [mAU*s]	Height [mAU]	Area %
1	12.979	MM	0.7227	2355.32739	54.31891	8.7240
2	16.657	MM	0.8775	2.46429e4	468.03088	91.2760

（峰面积：peak area）

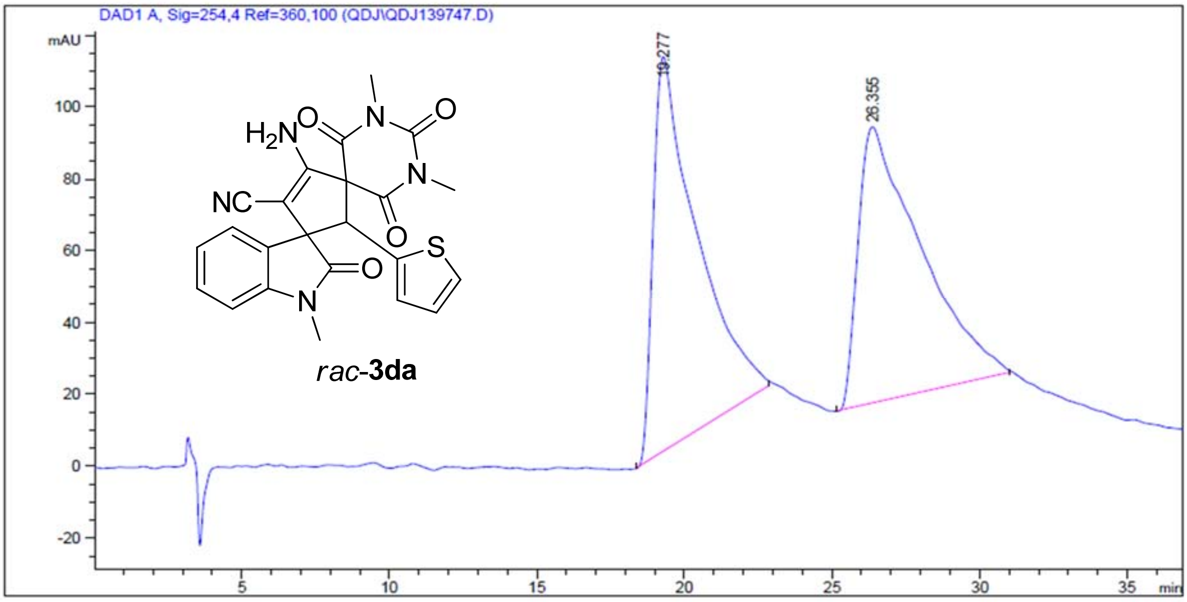

Peak #	RetTime [min]	Type	Width [min]	Area [mAU*s]	Height [mAU]	Area %
1	19.277	BB	1.3767	1.13497e4	109.91280	50.0510
2	26.355	BB	1.7334	1.13266e4	76.94252	49.9490

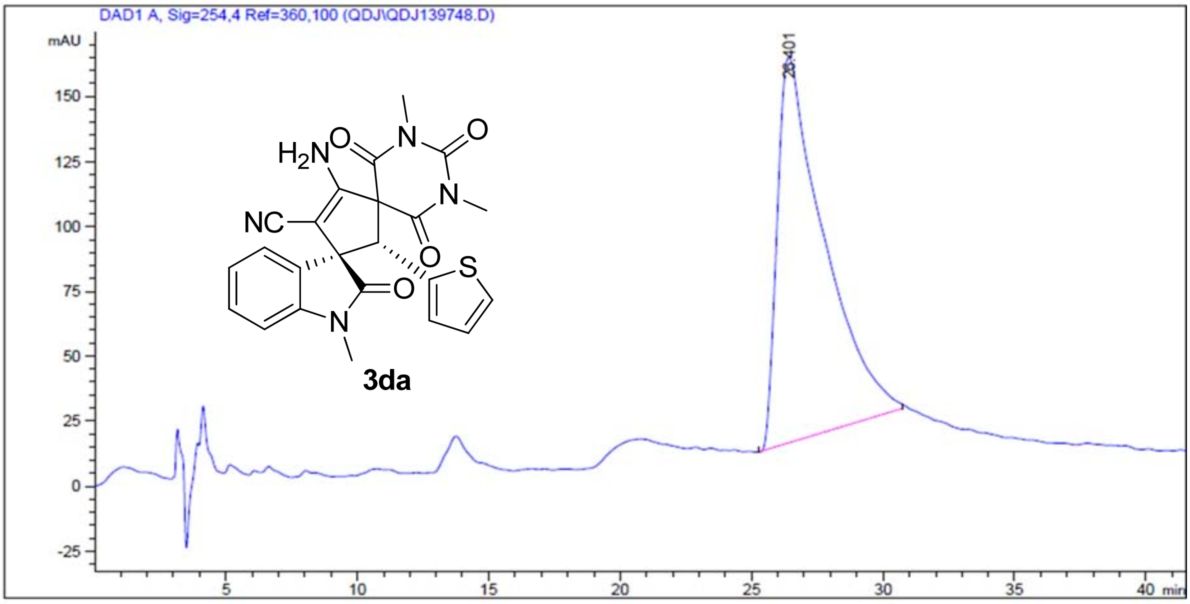

Peak #	RetTime [min]	Type	Width [min]	Area [mAU*s]	Height [mAU]	Area %
1	26.401	BB	1.6792	1.84783e4	148.75494	100.0000

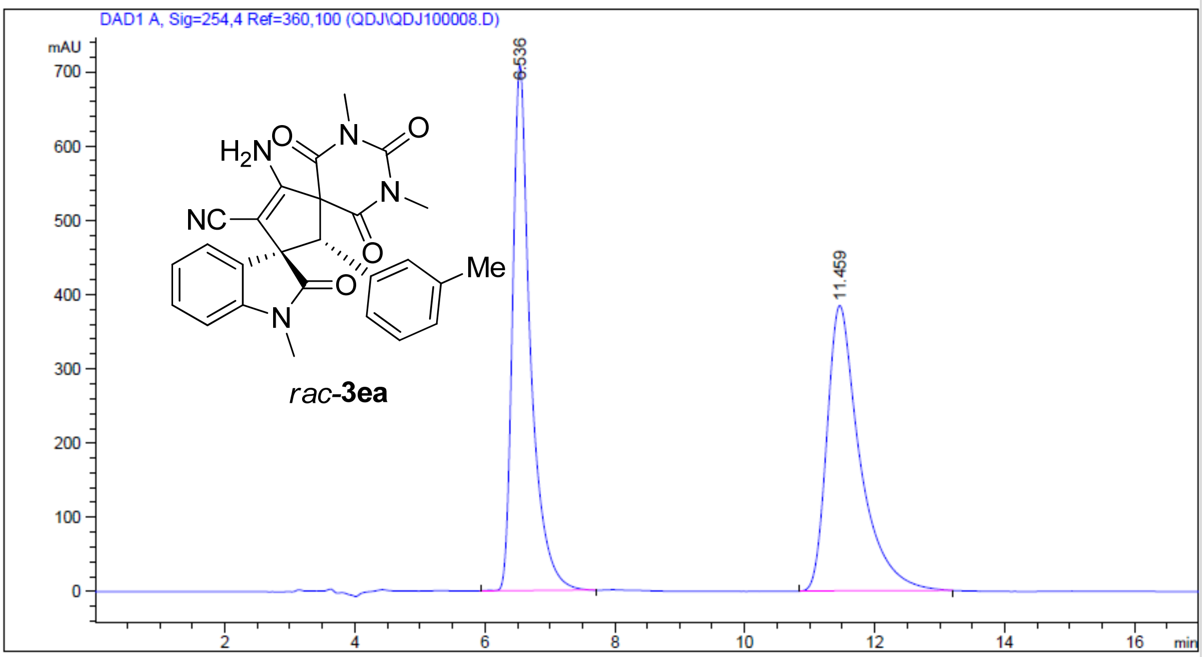

Peak #	RetTime [min]	Type	Width [min]	Area [mAU*s]	Height [mAU]	Area %
1	6.536	BB	0.2800	1.36046e4	708.44281	50.0884
2	11.459	BB	0.5170	1.35566e4	384.83334	49.9116

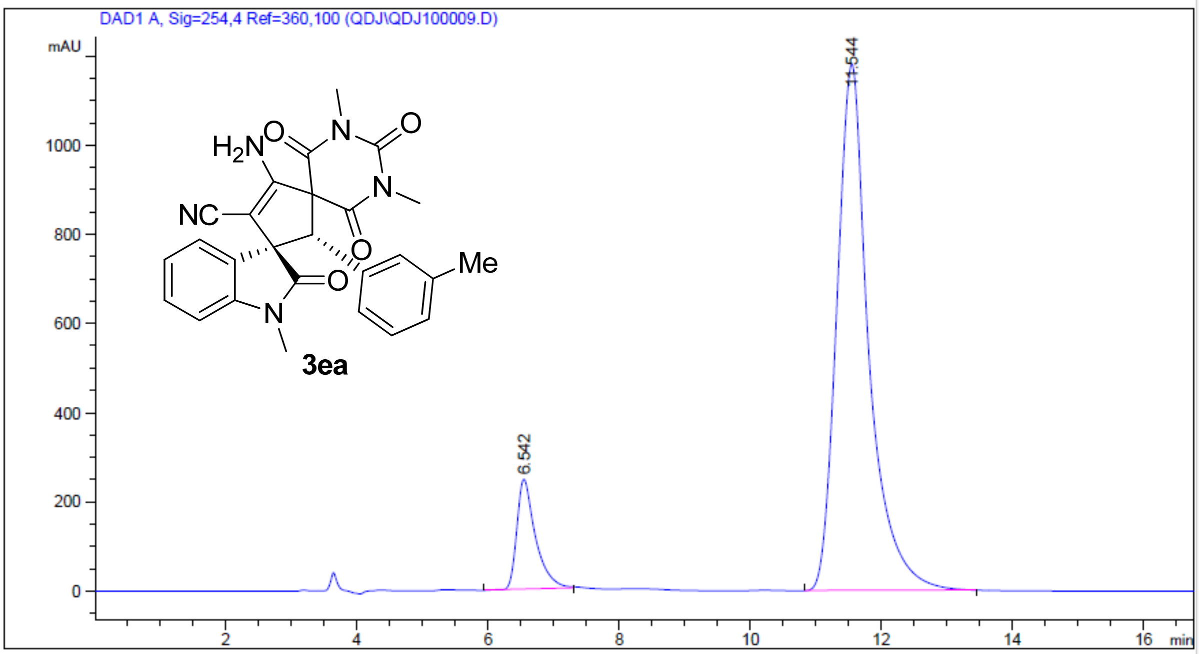

Peak #	RetTime [min]	Type	Width [min]	Area [mAU*s]	Height [mAU]	Area %
1	6.542	BB	0.2887	4879.15039	246.73080	10.8513
2	11.544	BB	0.5039	4.00844e4	1181.15967	89.1487

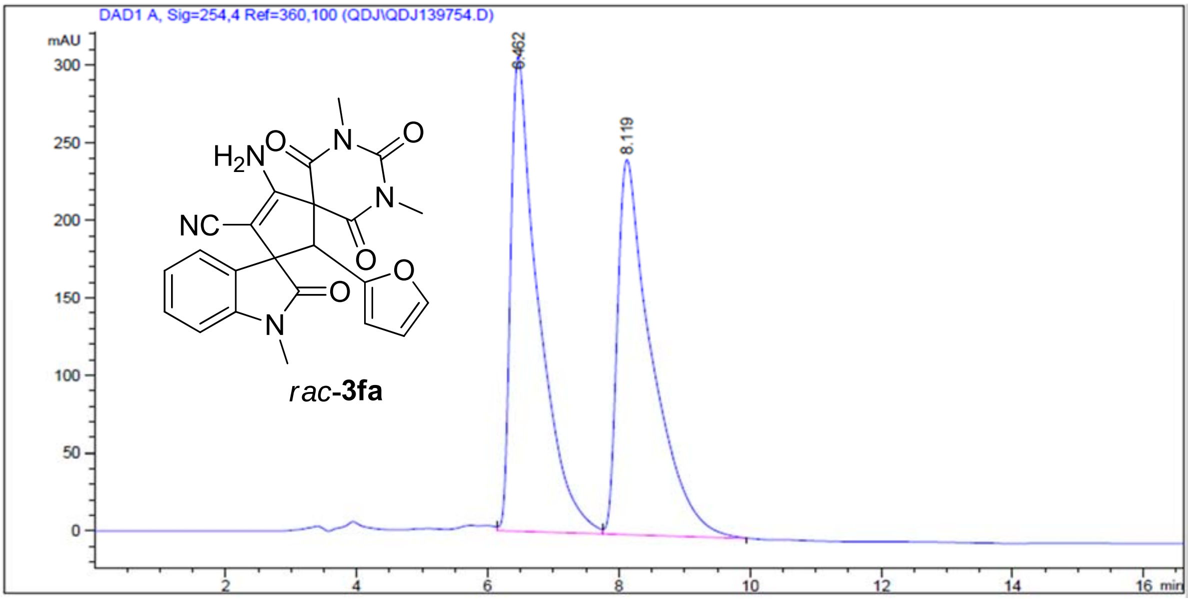

Peak #	RetTime [min]	Type	Width [min]	Area [mAU*s]	Height [mAU]	Area %
1	6.462	VV	0.4131	9056.08105	306.17358	50.6287
2	8.119	VB	0.5098	8831.16016	241.98680	49.3713

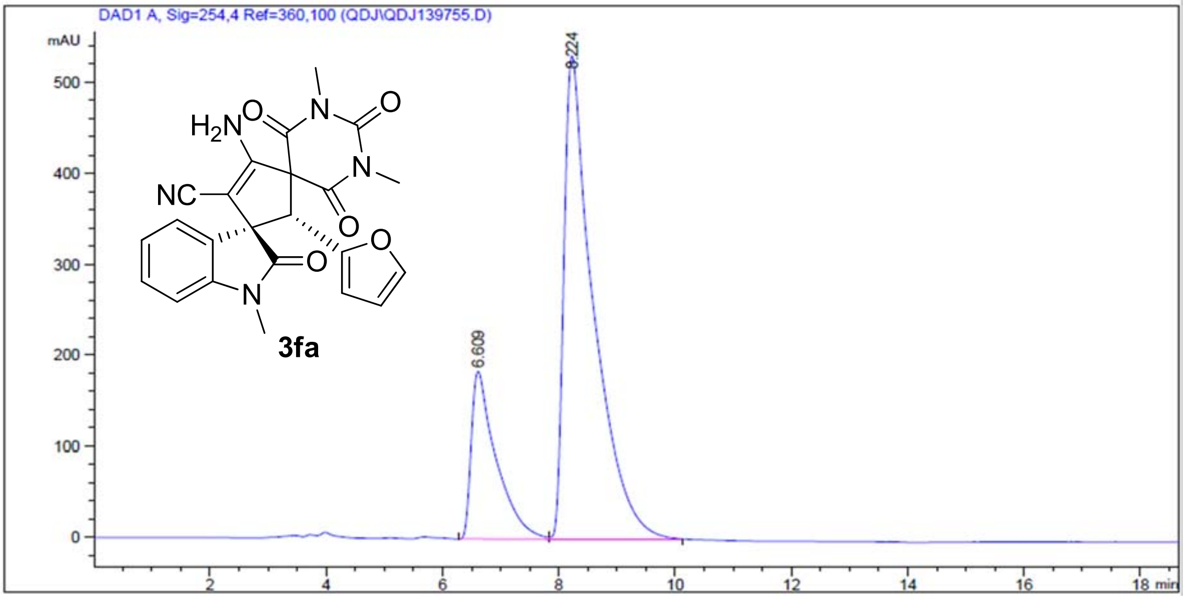

Peak #	RetTime [min]	Type	Width [min]	Area [mAU*s]	Height [mAU]	Area %
1	6.609	VV	0.4177	5481.59229	183.90752	22.4785
2	8.224	VB	0.4974	1.89043e4	530.94720	77.5215

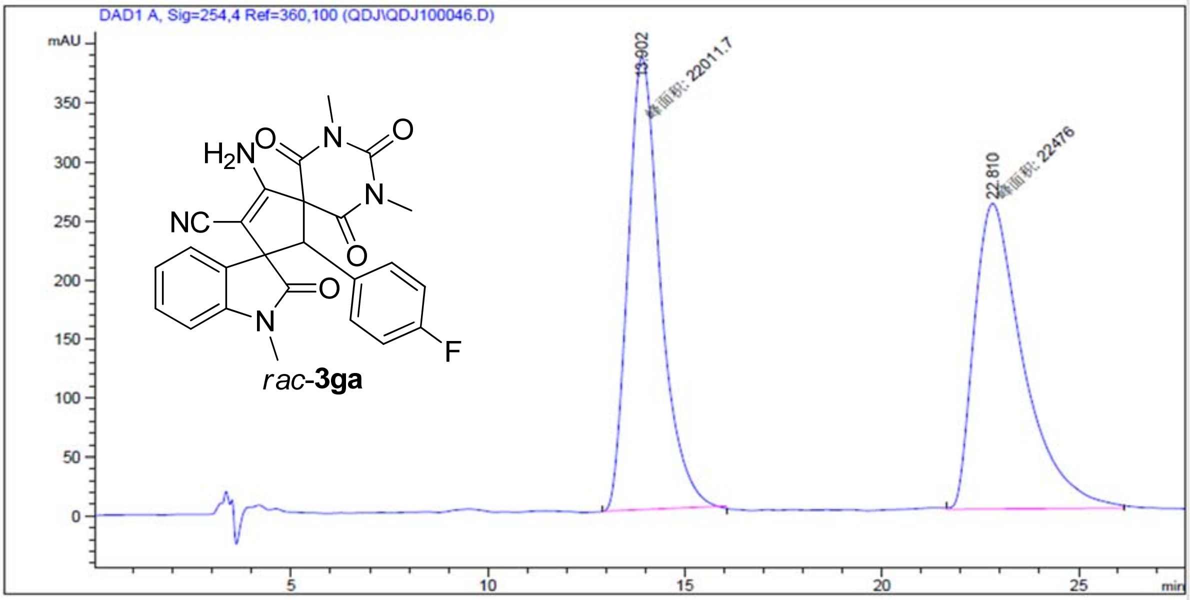

Peak #	RetTime [min]	Type	Width [min]	Area [mAU*s]	Height [mAU]	Area %
1	13.902	MM	0.9575	2.20117e4	383.16333	49.4782
2	22.810	MM	1.4452	2.24760e4	259.20590	50.5218

（峰面积：peak area）

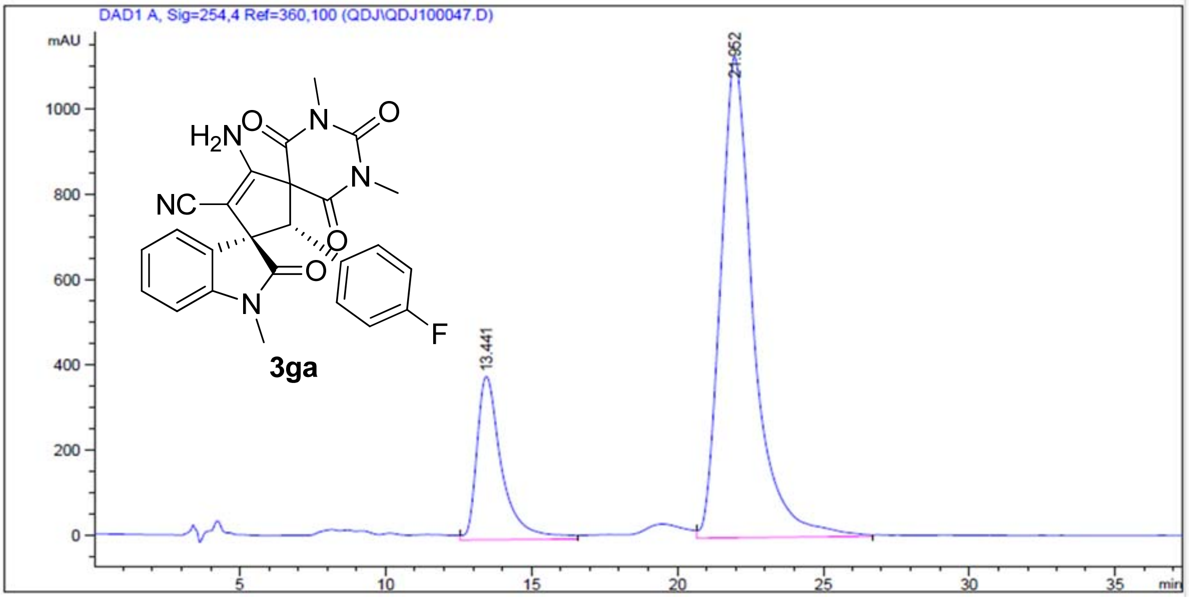

Peak #	RetTime [min]	Type	Width [min]	Area [mAU*s]	Height [mAU]	Area %
1	13.441	VV	0.8871	2.29297e4	382.65372	20.6138
2	21.952	VV	1.1859	8.83051e4	1127.34204	79.3862

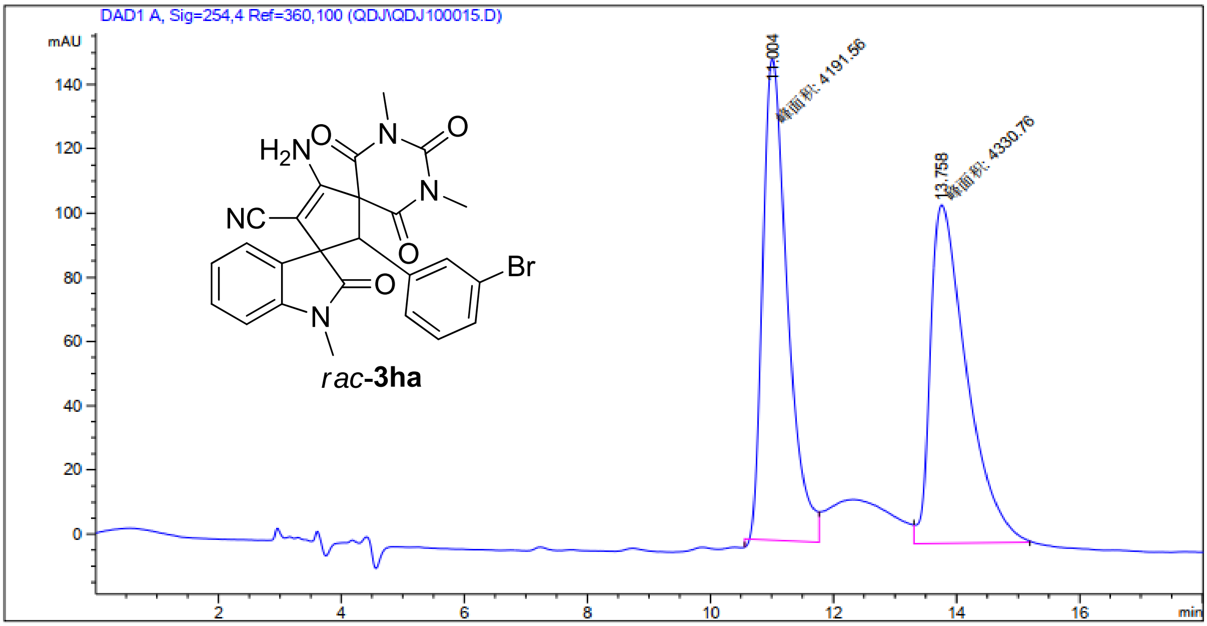

Peak #	RetTime [min]	Type	Width [min]	Area [mAU*s]	Height [mAU]	Area %
1	11.004	MM	0.4658	4191.56250	149.97838	49.1834
2	13.758	MM	0.6843	4330.75586	105.48135	50.8166

（峰面积：peak area）

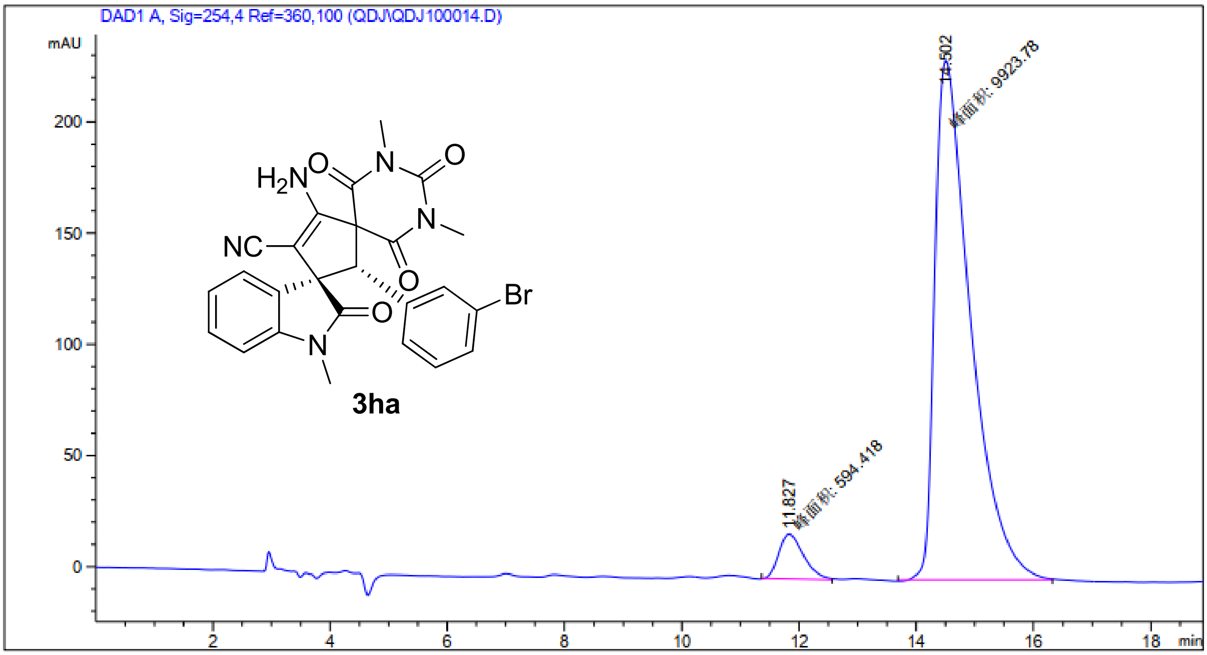

Peak #	RetTime [min]	Type	Width [min]	Area [mAU*s]	Height [mAU]	Area %
1	11.827	MM	0.4914	594.41797	20.16006	5.6513
2	14.502	MM	0.7088	9923.77539	233.34561	94.3487

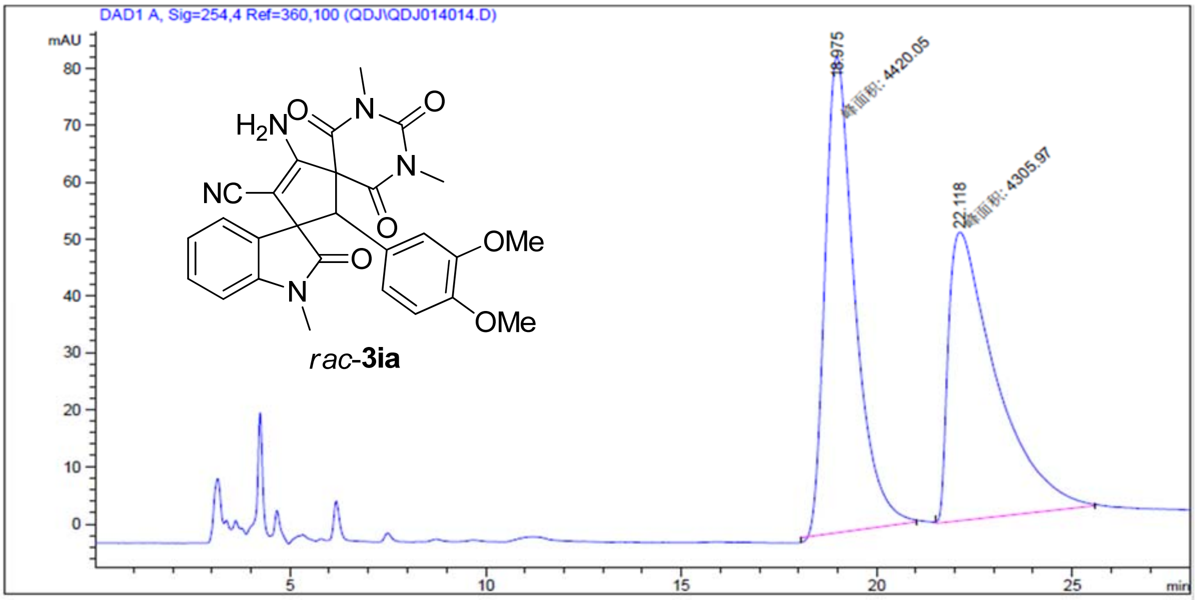

Peak #	RetTime [min]	Type	Width [min]	Area [mAU*s]	Height [mAU]	Area %
1	18.975	MM	0.8815	4420.04590	83.57407	50.6537
2	22.118	MM	1.4172	4305.96973	50.64063	49.3463

（峰面积：peak area）

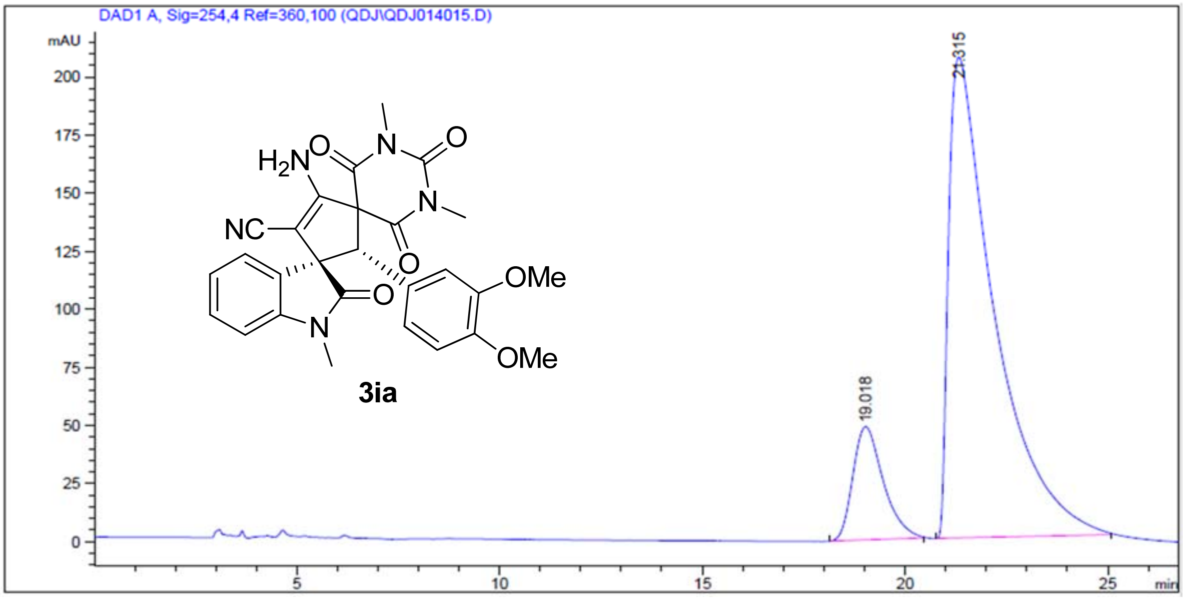

Peak #	RetTime [min]	Type	Width [min]	Area [mAU*s]	Height [mAU]	Area %
1	19.018	BB	0.7754	2555.64233	48.76661	13.5908
2	21.315	BB	1.0894	1.62486e4	207.09192	86.4092

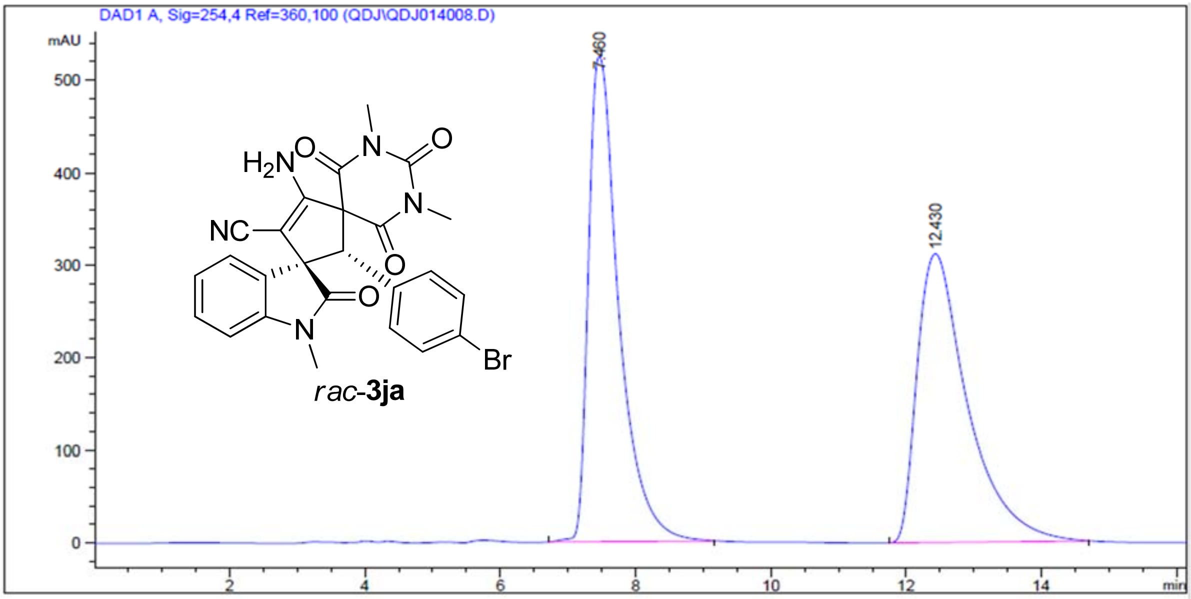

Peak #	RetTime [min]	Type	Width [min]	Area [mAU*s]	Height [mAU]	Area %
1	7.460	BB	0.4683	1.64594e4	524.27692	50.3802
2	12.430	BB	0.7778	1.62110e4	312.17899	49.6198

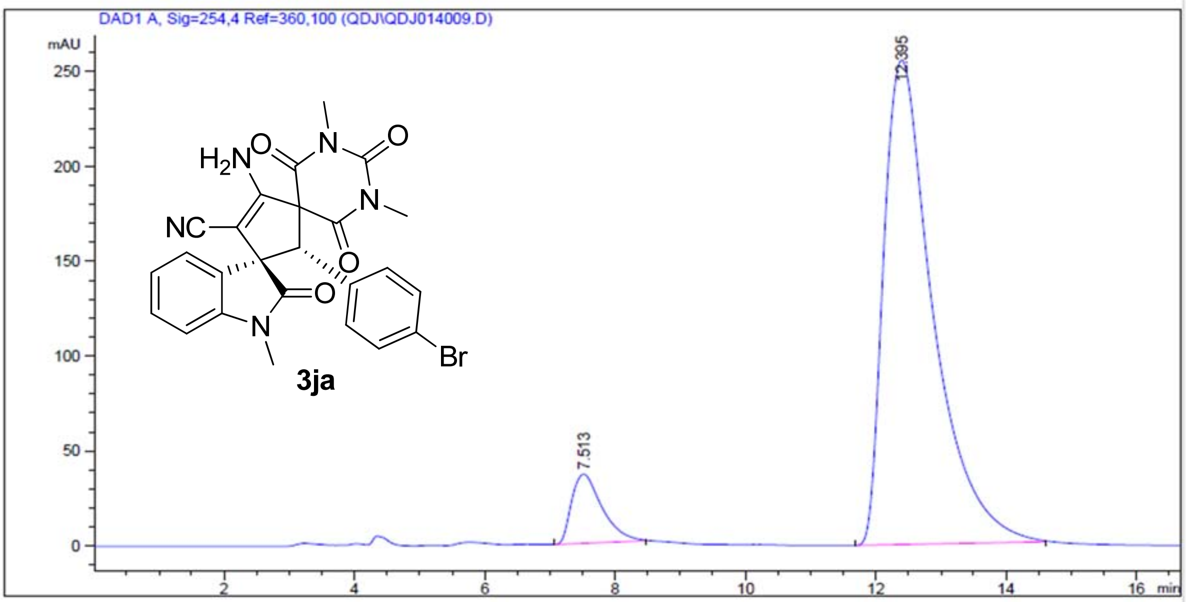

Peak #	RetTime [min]	Type	Width [min]	Area [mAU*s]	Height [mAU]	Area %
1	7.513	BB	0.4975	1191.26038	36.43427	8.1437
2	12.395	BB	0.7894	1.34368e4	255.57106	91.8563

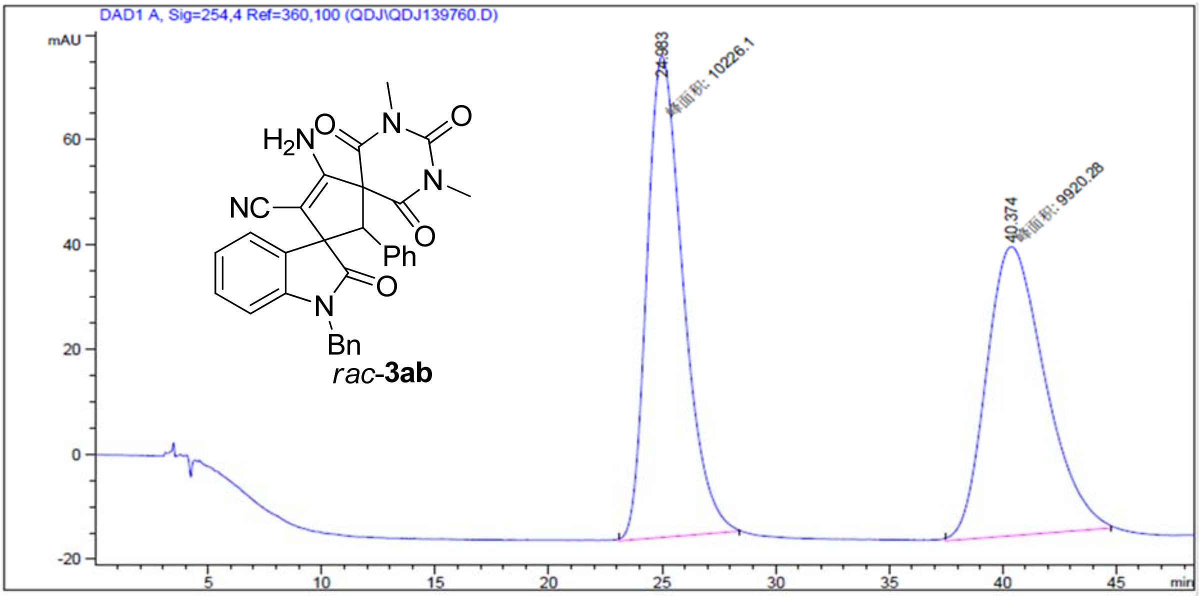

Peak #	RetTime [min]	Type	Width [min]	Area [mAU*s]	Height [mAU]	Area %
1	24.983	MM	1.8545	1.02261e4	91.90362	50.7590
2	40.374	MM	2.9952	9920.27930	55.20137	49.2410

（峰面积：peak area）

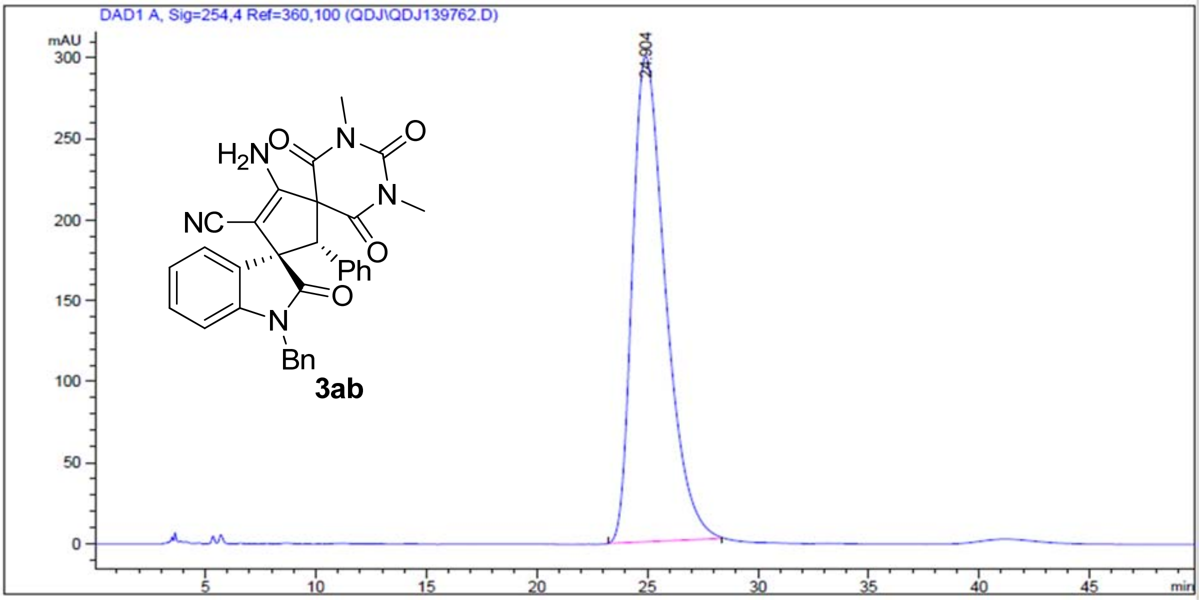

Peak #	RetTime [min]	Type	Width [min]	Area [mAU*s]	Height [mAU]	Area %
1	24.904	BB	1.5472	3.13476e4	299.96777	100.0000

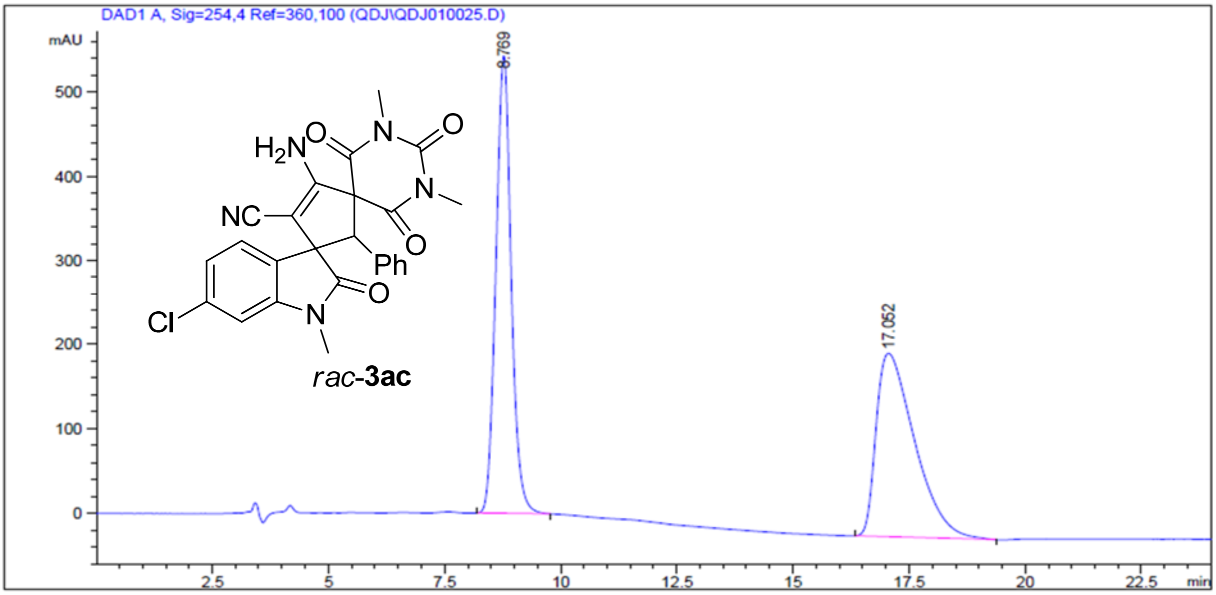

Peak #	RetTime [min]	Type	Width [min]	Area [mAU*s]	Height [mAU]	Area %
1	8.769	BB	0.3498	1.24712e4	543.22906	49.8799
2	17.052	BB	0.8575	1.25313e4	217.06197	50.1201

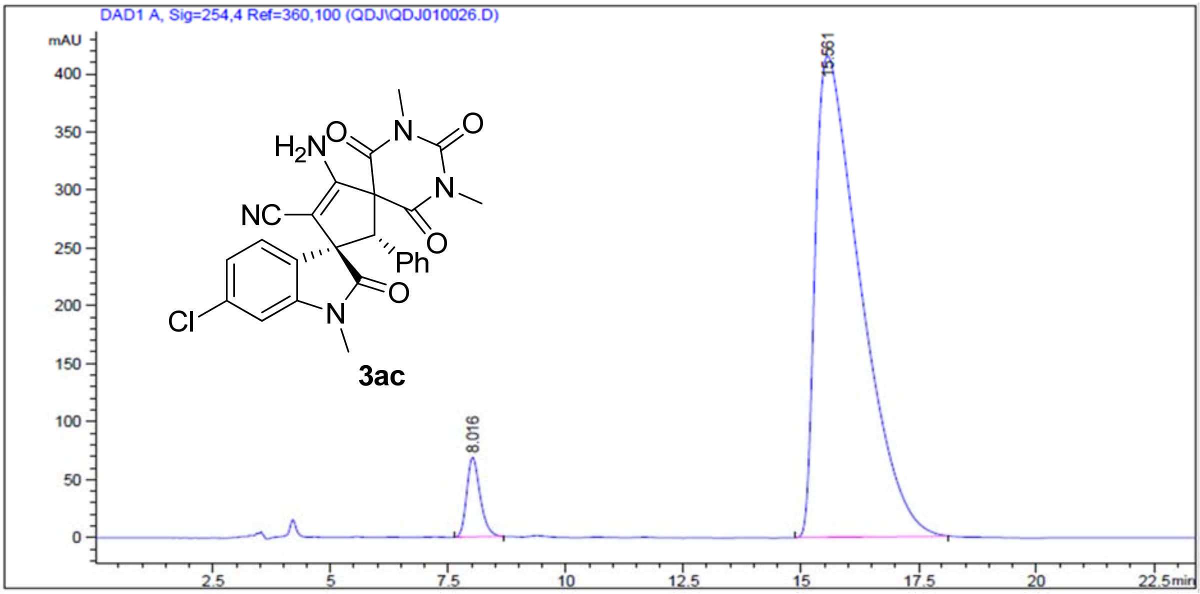

Peak #	RetTime [min]	Type	Width [min]	Area [mAU*s]	Height [mAU]	Area %
1	8.016	BB	0.3022	1366.07776	68.63458	4.7117
2	15.561	BB	0.9721	2.76274e4	415.32861	95.2883

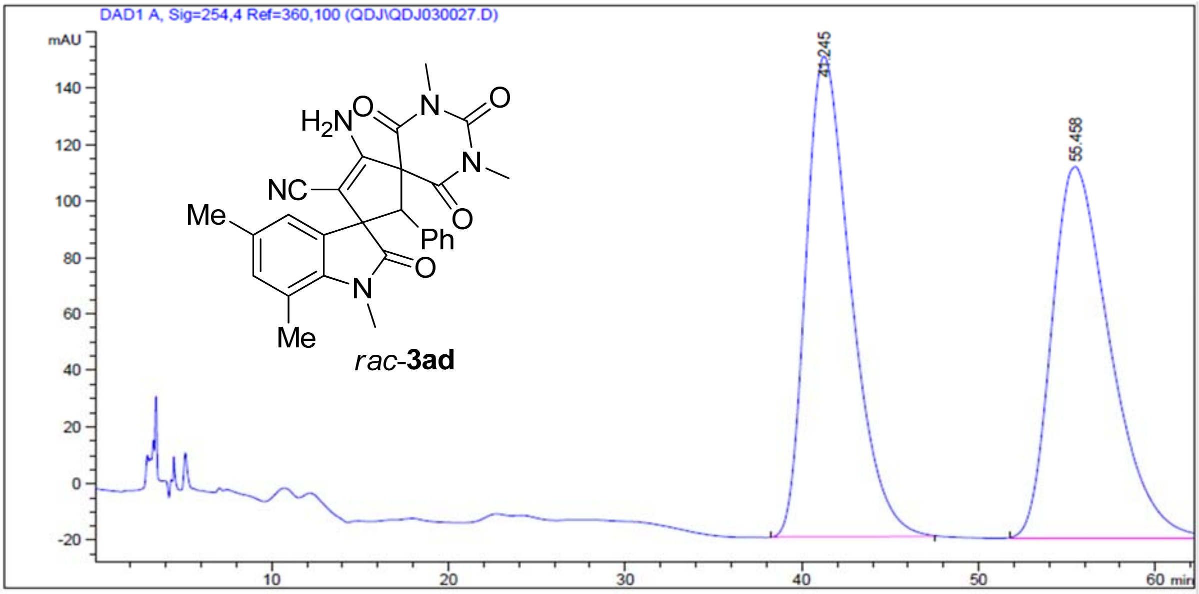

Peak #	RetTime [min]	Type	Width [min]	Area [mAU*s]	Height [mAU]	Area %
1	41.245	BB	2.6767	3.01995e4	170.31615	50.0102
2	55.458	BBA	3.2760	3.01871e4	131.60959	49.9898

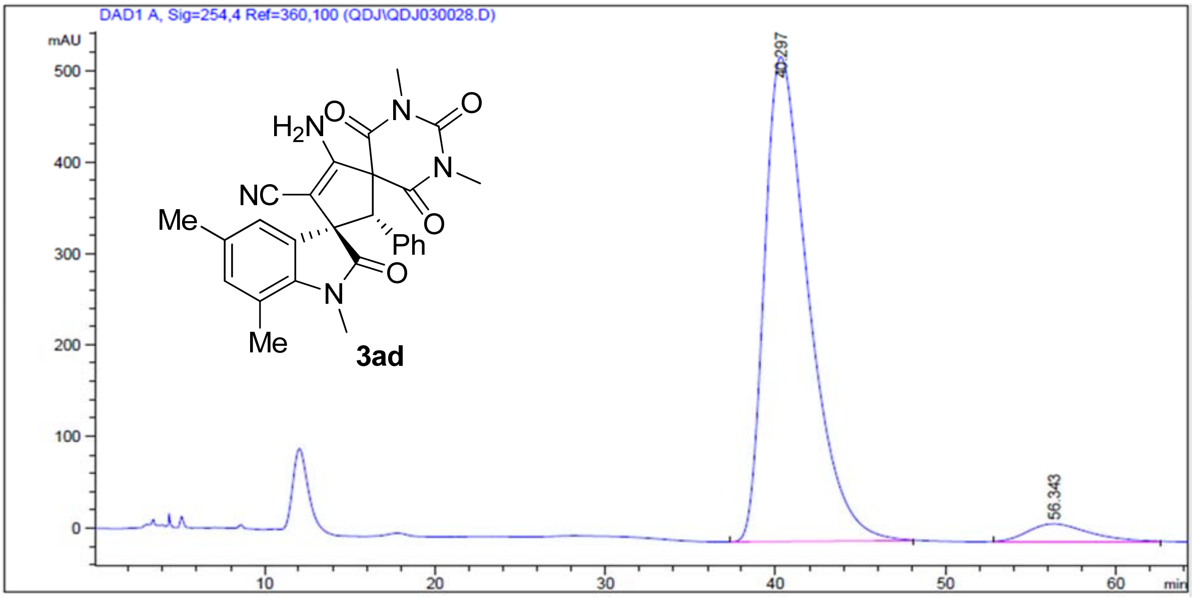

Peak #	RetTime [min]	Type	Width [min]	Area [mAU*s]	Height [mAU]	Area %
1	40.297	BB	2.5981	9.52733e4	529.76904	95.1467
2	56.343	BB	2.9537	4859.77344	19.40564	4.8533

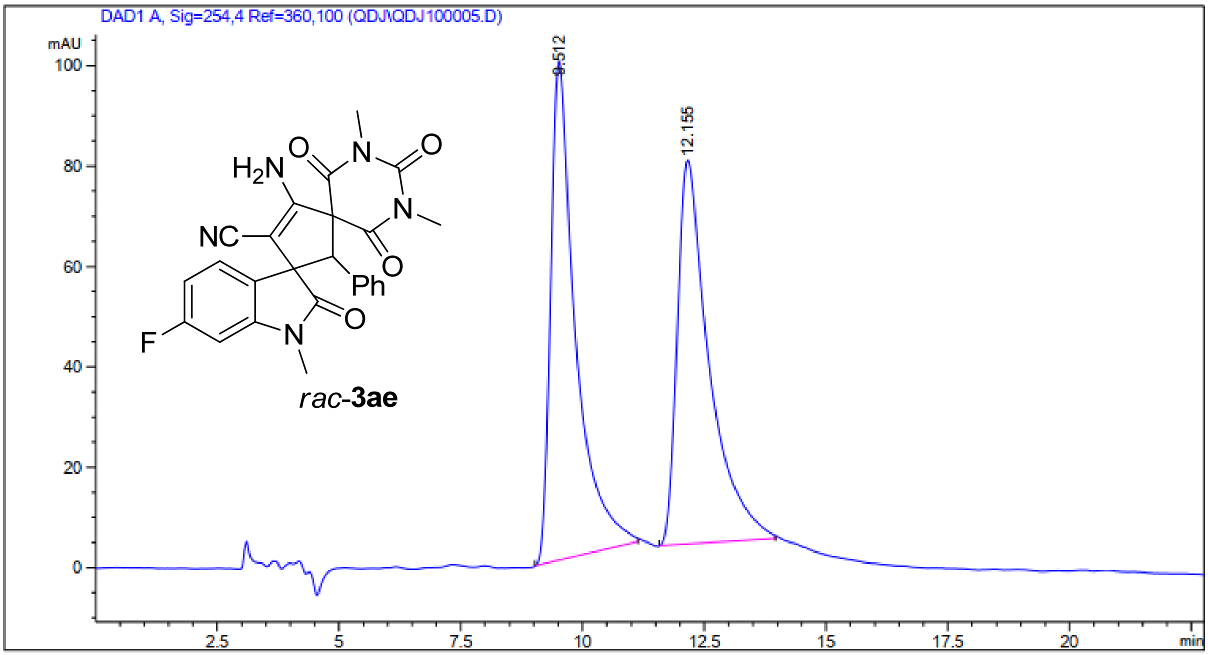

Peak #	RetTime [min]	Type	Width [min]	Area [mAU*s]	Height [mAU]	Area %
1	9.512	BB	0.5082	3548.33496	99.45790	50.4480
2	12.155	BB	0.6417	3485.31934	76.47534	49.5520

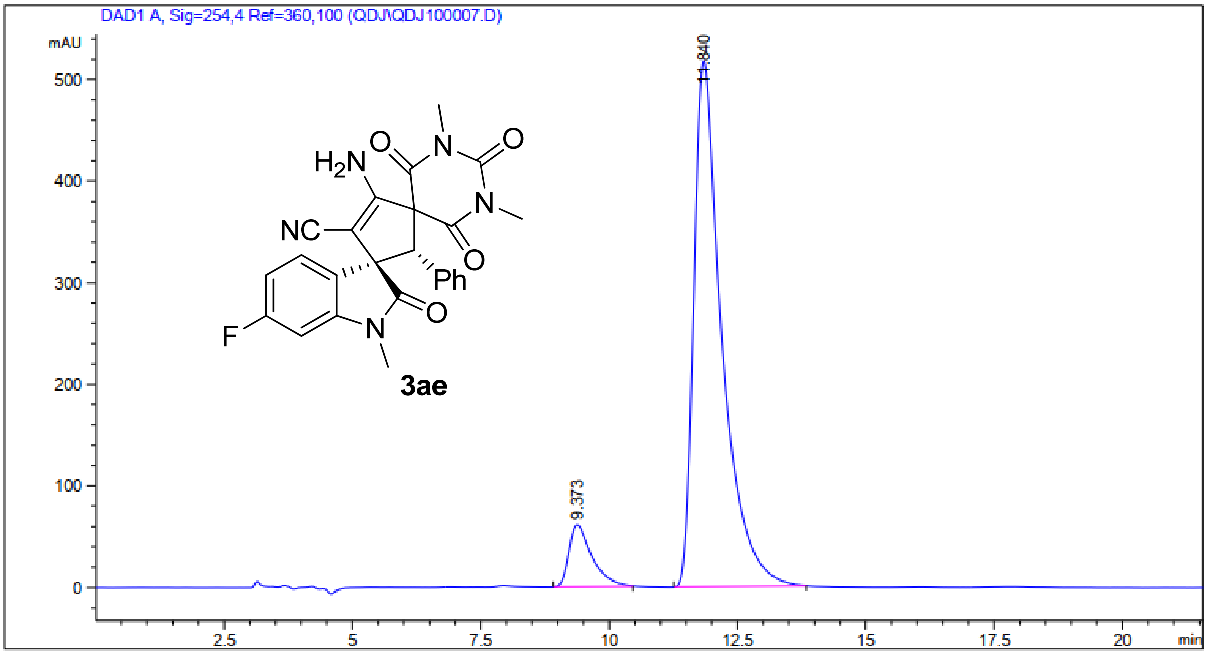

Peak #	RetTime [min]	Type	Width [min]	Area [mAU*s]	Height [mAU]	Area %
1	9.373	BB	0.4560	1905.36243	61.07478	8.8196
2	11.840	BB	0.5538	1.96984e4	517.87146	91.1804

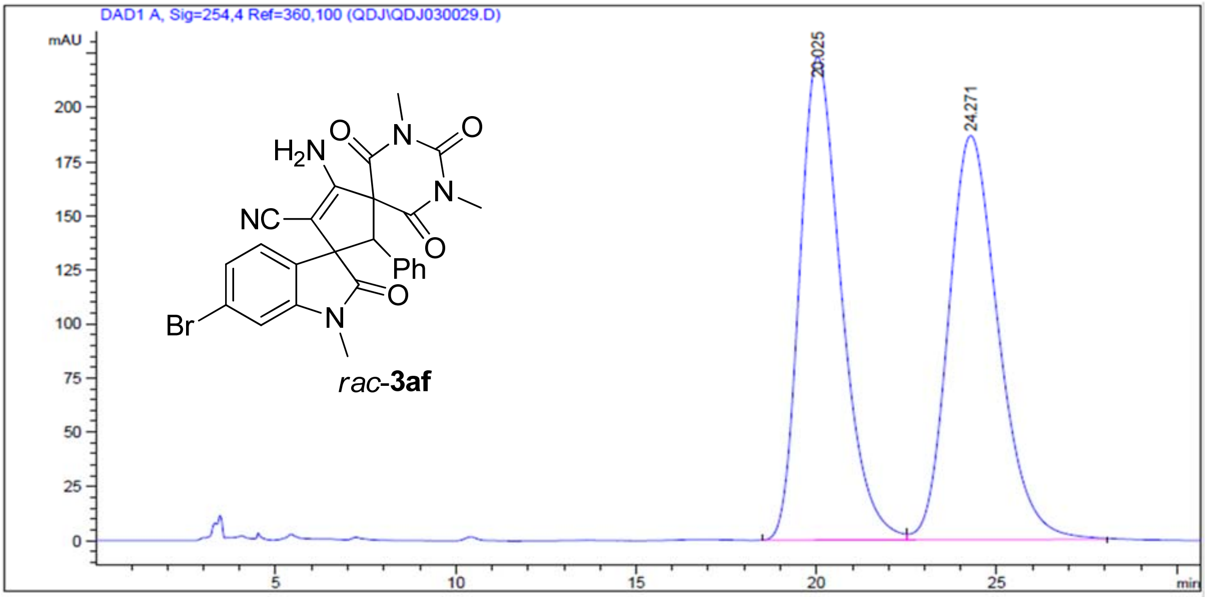

Peak #	RetTime [min]	Type	Width [min]	Area [mAU*s]	Height [mAU]	Area %
1	20.025	BV	1.2337	1.77821e4	223.15535	49.8288
2	24.271	VB	1.4969	1.79042e4	186.35692	50.1712

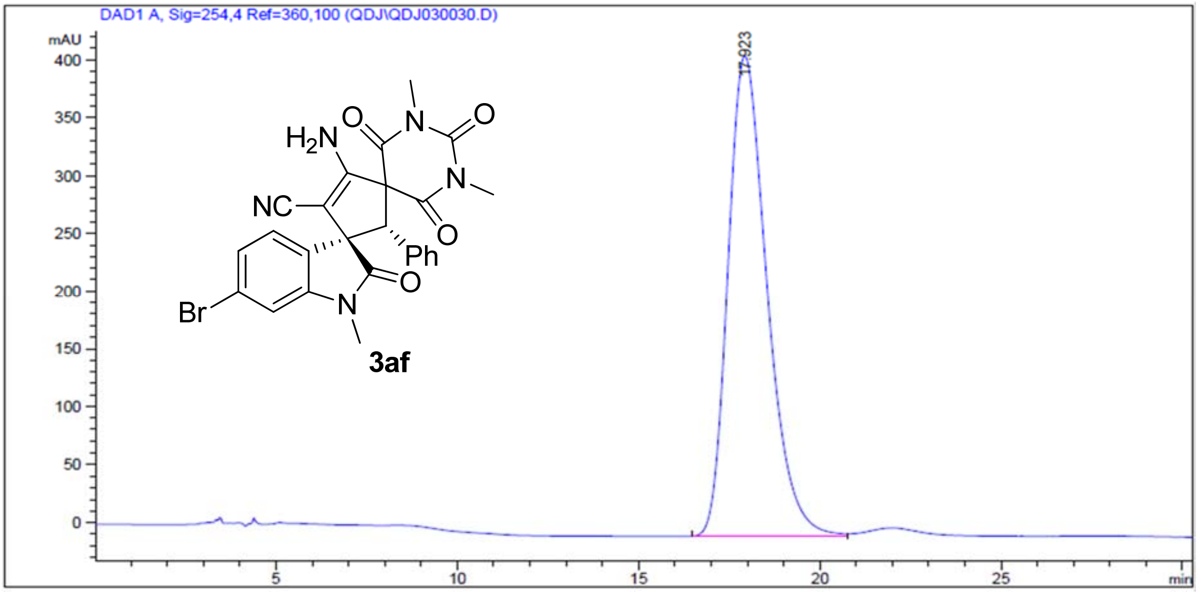

Peak #	RetTime [min]	Type	Width [min]	Area [mAU*s]	Height [mAU]	Area %
1	17.923	BV	1.1583	3.11083e4	415.12875	100.0000

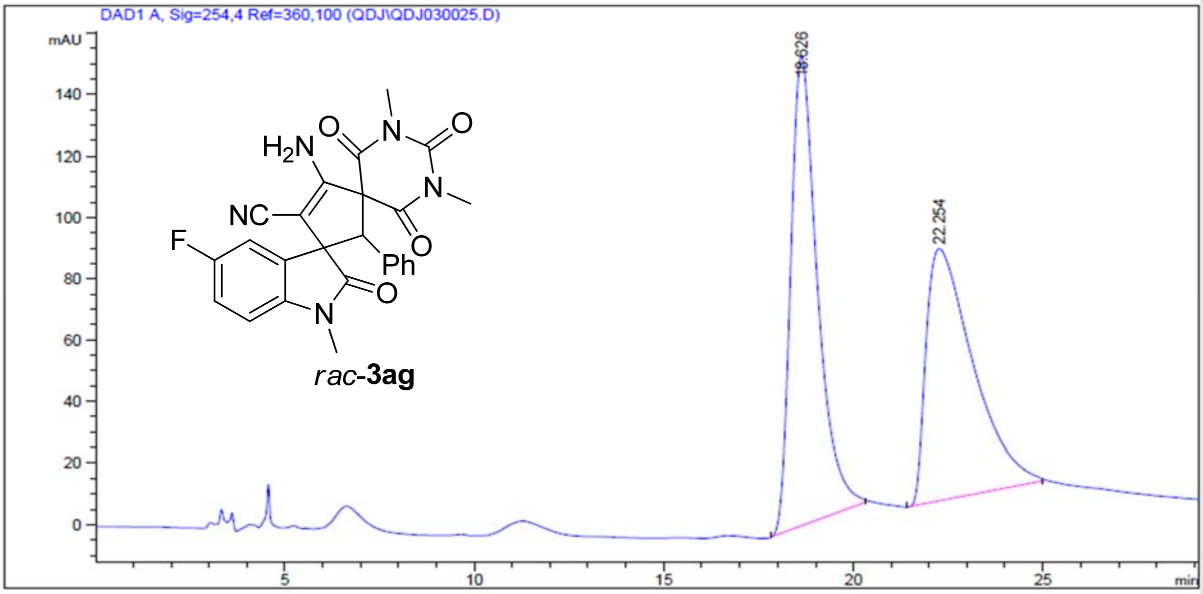

Peak #	RetTime [min]	Type	Width [min]	Area [mAU*s]	Height [mAU]	Area %
1	18.626	BB	0.7333	7511.52148	152.84779	51.5522
2	22.254	BB	1.1707	7059.18018	82.13405	48.4478

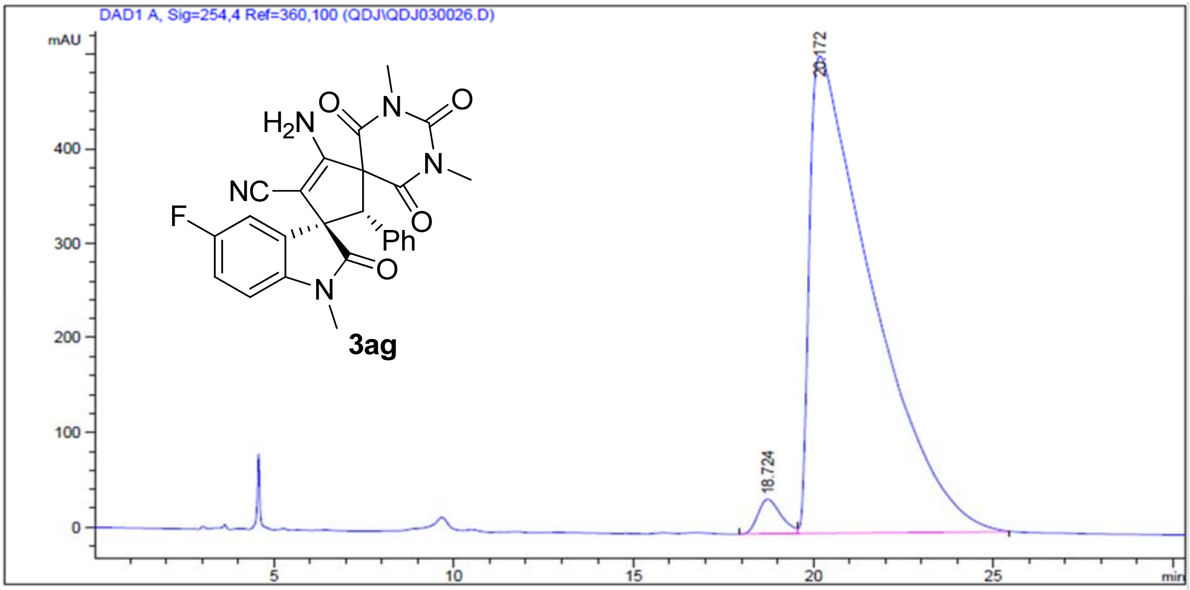

Peak #	RetTime [min]	Type	Width [min]	Area [mAU*s]	Height [mAU]	Area %
1	18.724	BV	0.6600	1654.62842	36.70240	2.6251
2	20.172	VB	1.6145	6.13763e4	504.79556	97.3749

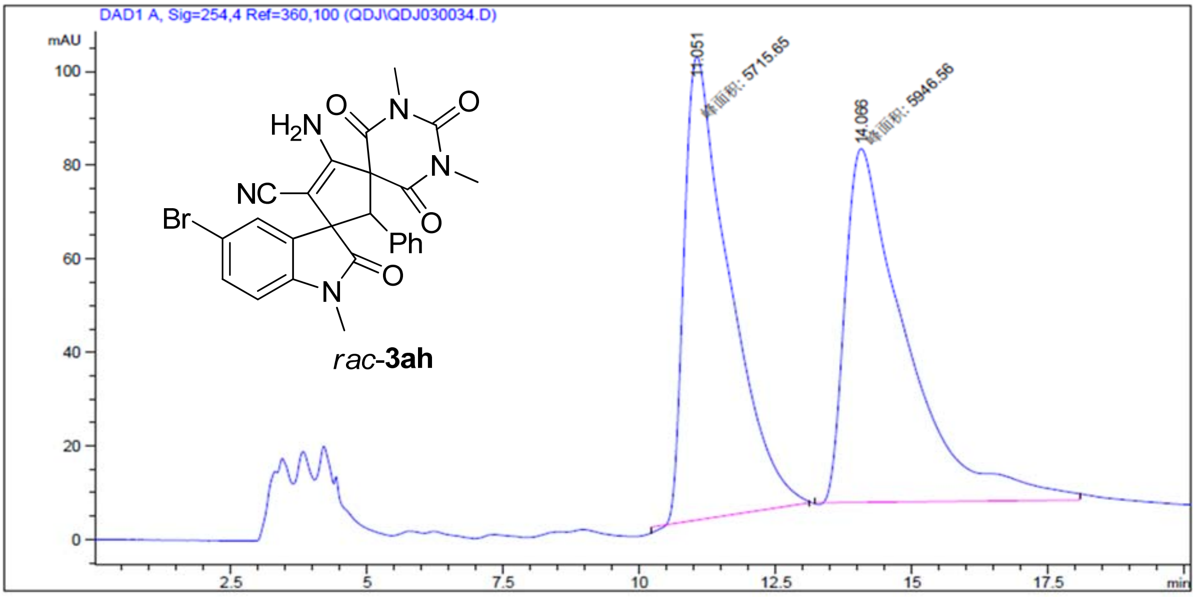

Peak	RetTime	Type	Width	Area	Height	Area
#	[min]		[min]	[mAU*s]	[mAU]	%
1	11.051	MM	0.9620	5715.64941	99.02480	49.0100
2	14.066	MM	1.3109	5946.55664	75.60430	50.9900

（峰面积：peak area）

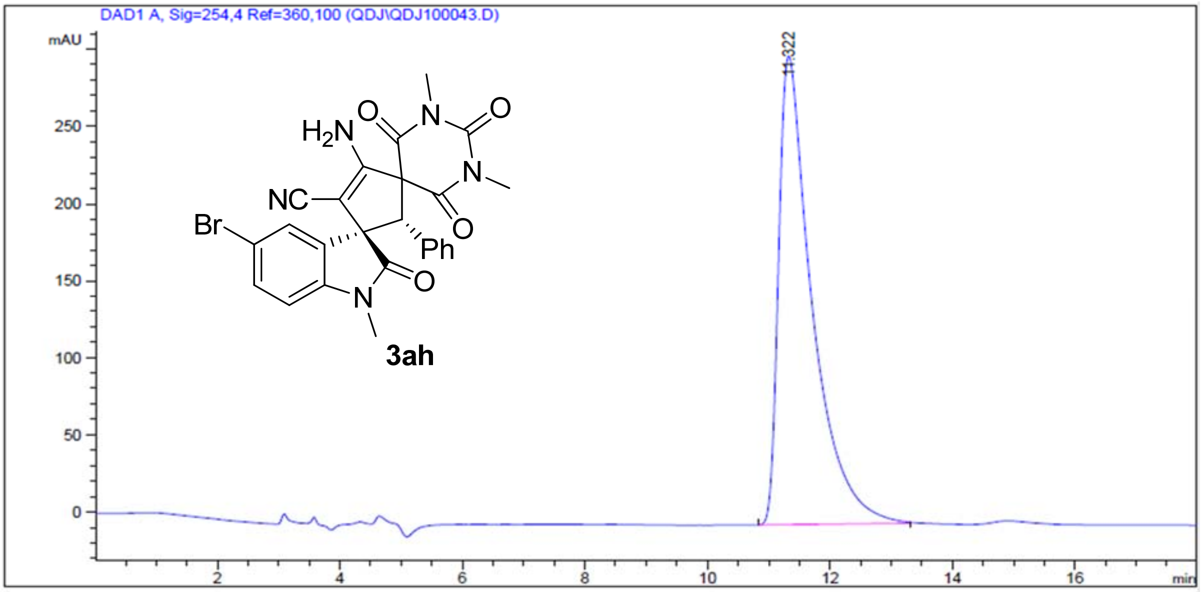

Peak	RetTime	Type	Width	Area	Height	Area
#	[min]		[min]	[mAU*s]	[mAU]	%
1	11.322	BB	0.5471	1.15330e4	303.65527	100.0000

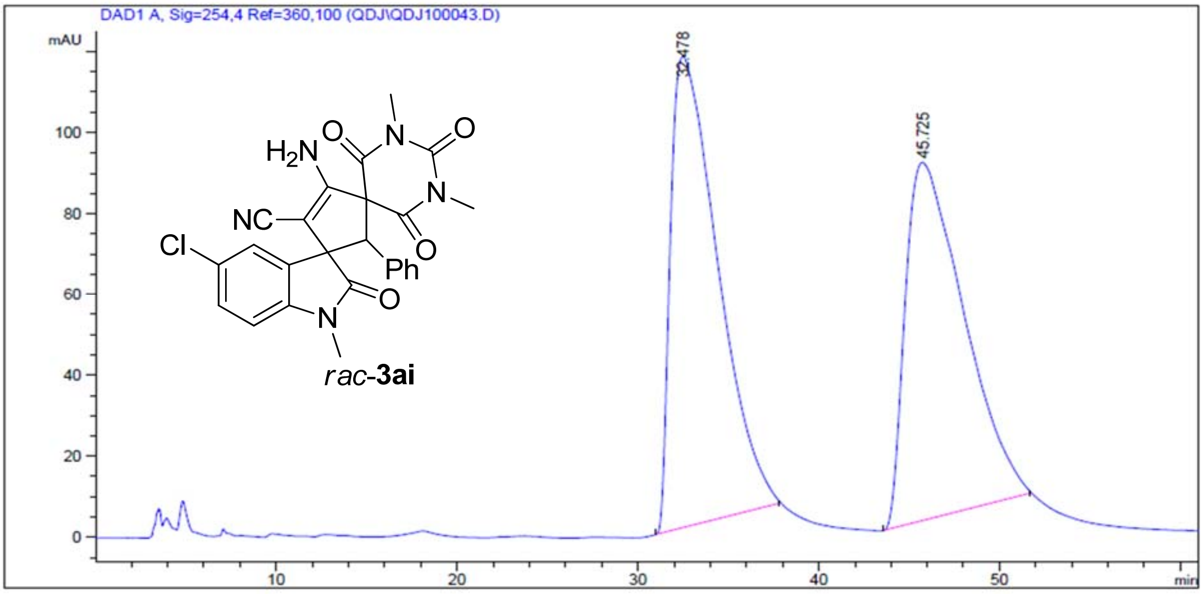

Peak #	RetTime [min]	Type	Width [min]	Area [mAU*s]	Height [mAU]	Area %
1	32.478	BB	2.3825	2.10775e4	116.40337	51.4196
2	45.725	BB	2.6385	1.99137e4	88.59107	48.5804

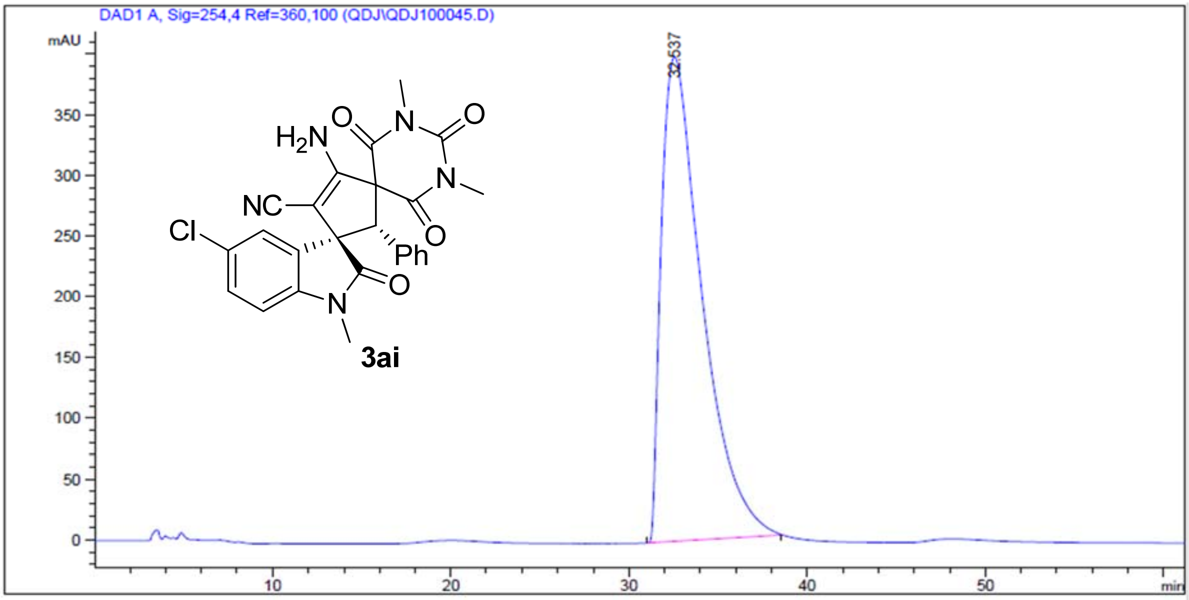

Peak #	RetTime [min]	Type	Width [min]	Area [mAU*s]	Height [mAU]	Area %
1	32.537	BB	2.2505	6.34293e4	398.61462	100.0000

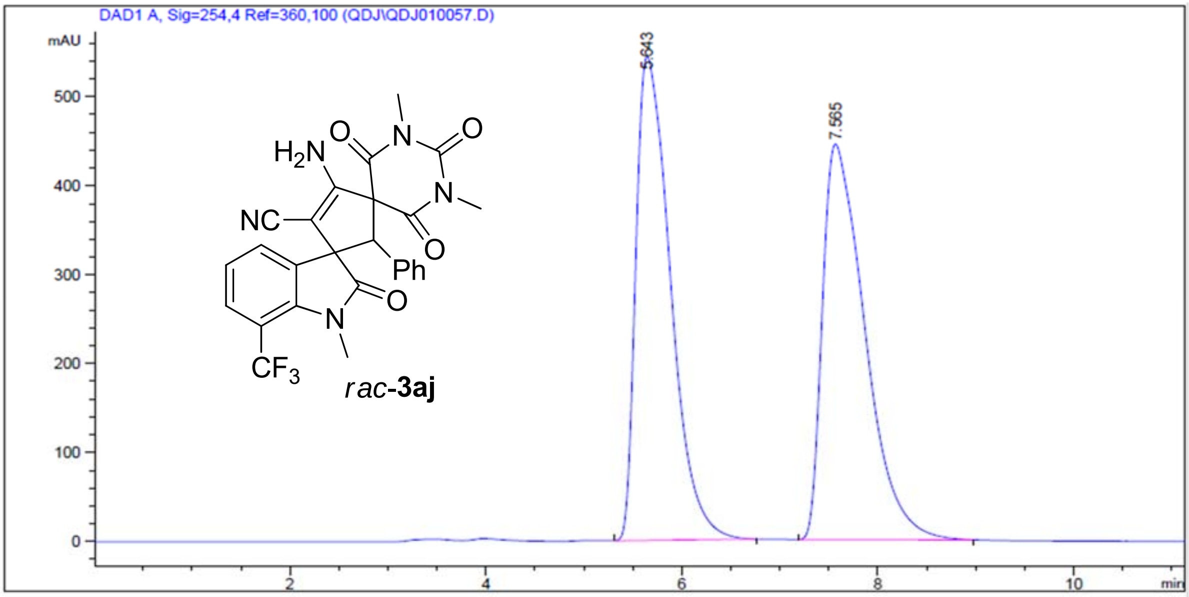

Peak #	RetTime [min]	Type	Width [min]	Area [mAU*s]	Height [mAU]	Area %
1	5.643	BB	0.3903	1.33540e4	544.08850	49.9590
2	7.565	BB	0.4788	1.33759e4	444.82416	50.0410

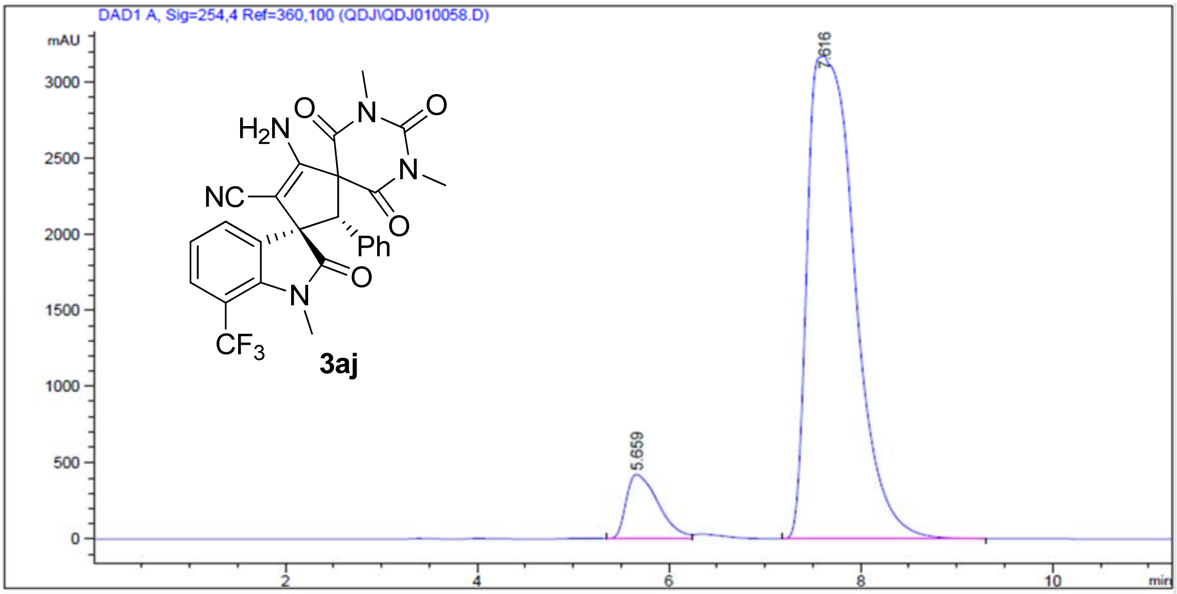

Peak #	RetTime [min]	Type	Width [min]	Area [mAU*s]	Height [mAU]	Area %
1	5.659	VV	0.3716	9878.32910	424.39972	8.3496
2	7.616	BB	0.4088	1.08430e5	3171.68530	91.6504

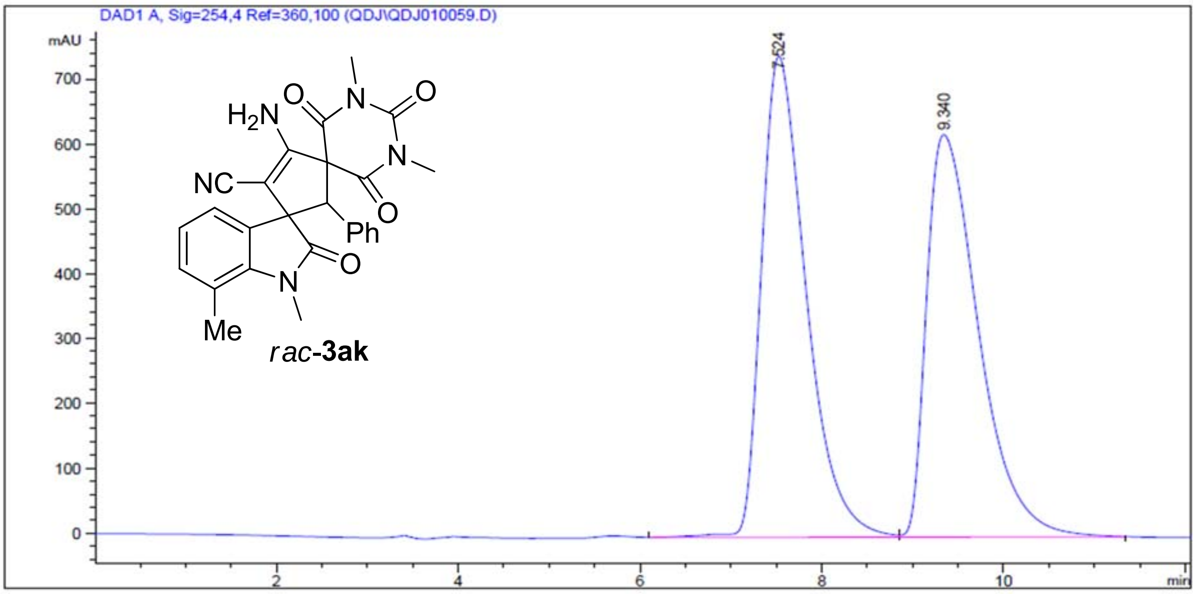

Peak #	RetTime [min]	Type	Width [min]	Area [mAU*s]	Height [mAU]	Area %
1	7.524	VV	0.5310	2.53685e4	741.74432	50.7140
2	9.340	VB	0.6177	2.46541e4	620.24689	49.2860

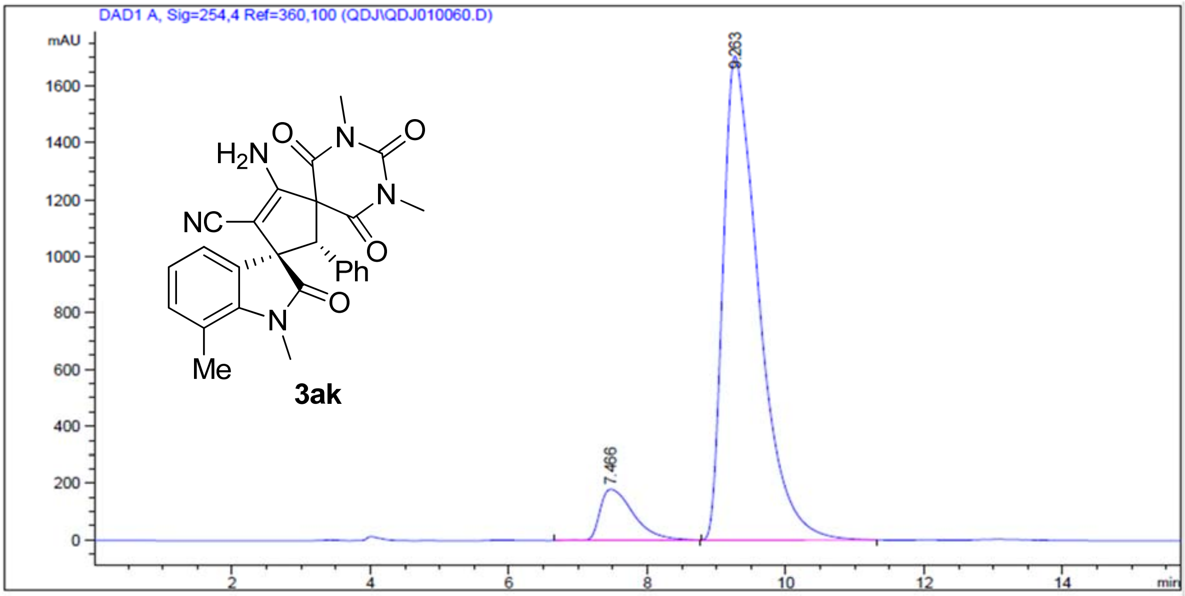

Peak #	RetTime [min]	Type	Width [min]	Area [mAU*s]	Height [mAU]	Area %
1	7.466	BB	0.5302	6140.44238	180.81670	9.1617
2	9.263	BB	0.5163	6.08822e4	1706.40686	90.8383
